# Supplementary material for: Systematic Review of Fear of Cancer Recurrence Patient-Reported Outcome Measures: Evaluating Methodological Quality and Measurement Properties Using the COSMIN Checklist
Source: Healthcare (Basel). 2025 Aug 29;13(17):2165. doi: 10.3390/healthcare13172165 (PMC12427728; doi:10.3390/healthcare13172165)
Supplement: Supplementary file 1 [file healthcare-13-02165-s001.zip › healthcare-3783344-supplementary.pdf]

**Supplemental A: Detailed determination of rating per measurement property and overall study quality and category**

**and Supplemental B. Top 5 PROMs Recommended for Clinical Use**


Supplemental A: Detailed determination of rating per measurement property and overall study quality 3

1. Established PROMs with Cultural or Population-Specific Validation 3

1.1 Fear of Cancer Recurrence Inventory (FCRI) – Adaptation and Validation in New Languages and Populations 3

Lebel et al., 2016 / FCRI [19] 3

Shin, 2017 / K-FCRI [20] 6

Van helmondt et al., 2017 / FCRI-NL (Dutch) [21] 9

Hovdenak Jakobsen et al., 2018 / FCRI-Danish version [22] 11

Liu et al., 2020 / Mandarin version FCRI [23] 14

Xu et al., 2021 / FCRI-C (Chinese) and 2.1 Short version of FCRI-C [24] 17

1.2 Concerns About Recurrence Scale (CARS) [53] – Adaptation and Validation in a New Language and Population 20

Monimo et al., 2014 / CARS-J [25] 20

2. FCRI-Derived Short Forms – Development, Validation, and Cross-Cultural Adaptation 23

2.1 FCRI (Simard and Savard, 2009) [5]—Shortened and Adapted Versions 23

Costa et al., 2016 / FCRI short form [26] 23

Eyrenci and Sertel Berk, 2018 / FCRI Turkish Version [27] 26

Xu et al. [24] FCRI-C Short Version 29

2.2 FCRI Short Form (FCRI-SF) [28] – Screening Variants and cut-off versions 29

Simard and Savard., 2015 / FCRI-SF [[28] 29

Fardell et al., 2018 / FCRI-SF [29] 32

Peng et al., 2019 / Chinese Version of the FCRI-SF [30] 34

Decat Bergerot et al., 2023 FCRI-SF [31] 37

2.3 Fear of Progression Questionnaire Short Form (FoP-Q-SF) [32] and FoP-Q [33] – Adaptations and Validations in New Languages and Populations 40

Mahendran et al., 2020 / FoP-Q-SF [34] 40

Abd Hamid et al., 2021 / FoP-Q-SF-M [35] 42

Youssef et al., 2021 / FoP-Q-RS [36] 45

Cheng et al., 2022 / FoP-Q-SF [37] 48

Silva et al., 2022 / FoP-Q-SF [38] 51

Hasannezhad Reskati et al., 2022 / FoP-Q [39] 53

3. New Developed FCR PROMs 56

3.1 Cancer Worry Scale (CWS) (Custers et al., 2014) [40]: Validation and Adaptation of the New PROM in New Languages and Populations 56

Custers et al., 2014 / CWS-8 items [40] 56

Custers et al., 2018 / CWS-6 items [41] 59

Chirico 2022 / CWS-8 items [42] 61
3.2 FCR4 and FCR7 (Humphris et al., 2018) [43]: Validation and Adaptation of the New PROM in New Languages and Populations 64
Humphris et al., 2018 / FCR4/FCR7 [43] 64
Yang et al., 2019 / Chinese version of the FCR723 FCR7 [44] 67
Lee et al. 2020 / FCR7-Chinese [45] 69
Braun et al. 2022 / FCR6-Brain [46] 72
Iglesias-Puzas et al. 2022 / FCR7-Spanish [47] 75
Nandakumar et al., 2022 [48] 77
Decat Bergerot et al., 2023 FCR4/FCR7 [31] 80
3.3 Concerns About Recurrence Questionnaire (CARQ) CARQ-4 [49]): Validation of the New PROM 83
Thewes et al., 2015 / CARQ-4 [49] 83
3.4 FCR-1 (Rudy et al., 2020) [50] - Validation of a New PROM, and Adaptations in New Languages and Populations 85
Rudy et al., 2020 / FCR-1 [50] 85
Smith et al., 2023 / FCR-1r [51] 88
Lyhne et al., 2023 / FCR-1 Danish [52] 92
Supplemental B. Top 5 PROMs Recommended for Clinical Use 95

**Supplemental A: Detailed determination of rating per measurement property and overall study quality**

**1. Established PROMs with Cultural or Population-Specific Validation**

**1.1 Fear of Cancer Recurrence Inventory (FCRI) – Adaptation and Validation in New Languages and Populations**
**Lebel et al., 2016 / FCRI [19]**

**Reference:** Lebel, S., Simard, S., Harris, C., et al. (2016). Empirical validation of the English version of the Fear of Cancer Recurrence Inventory (FCRI). *Quality of Life Research*, 25(2), 311-321. DOI: 10.1007/s11136-015-1088-2

**Content Validity Assessment: Sufficient**
The FCRI was developed to assess fear of cancer recurrence in cancer survivors, based on a cognitive-behavioral conceptualization of the construct. The scale was translated using a forward-backward method and pilot-tested to ensure semantic equivalence and cultural appropriateness. It includes items assessing key aspects of FCR such as triggers, severity, psychological distress, and coping strategies. Given its development process and demonstrated psychometric properties, the content validity is considered sufficient.

Permission from original author(s) of the scale: Yes
Original authors involved in the study team: Yes

**Measurement Property Assessment:**

**1. Structural Validity (mp1):**

**Property Rating**: +
**Data**: Confirmatory factor analysis (CFA) supported the seven-factor structure, with fit indices as follows: CFI = 0.96, NFI = 0.95, RMSEA = 0.08, SRMR = 0.09; Modifications were made to improve fit, with final model fit indices as: CFI = 0.98, RMSEA = 0.06, SRMR = 0.08.
**Methodological Quality**: Very Good
CFI met the threshold, while RMSEA and SRMR exceeded initial thresholds, post-hoc modifications led to acceptable fit indices.
**Quality of Evidence**: High
**Justification**: The refined CFA model met COSMIN fit indices and confirmed the expected second-order structure. No downgrade necessary.

**2. Internal Consistency (mp2):**

**Property Rating**: +
**Data**: Cronbach's alpha for the total scale and subscales:
Total scale: $\alpha = 0.96$, subscales: $\alpha = 0.71$–$0.94$.
**Methodological Quality**: Very Good
Cronbach's alpha values were consistently high, indicating excellent internal consistency.
**Quality of Evidence**: High
**Justification**: Alpha values exceeded .70, and sufficient structural validity supports interpretation. No downgrade necessary due to strong internal consistency across subscales.

**3. Reliability (mp3):**

**Property Rating**: +
**Data**: Test-retest reliability for the total scale and subscales:
Total scale: ICC = 0.94, subscales: ICC = 0.76–0.96.
**Methodological Quality**: Very Good
High ICC values indicate strong reliability over time.
**Quality of Evidence**: High
**Justification**: Strong test-retest reliability over 1 month in a large sample confirms high-quality evidence.

**4. Measurement Error (mp4):**

**Property Rating**: ?
**Data**: Measurement error was not explicitly reported such as for No SEM or LoA.
**Methodological Quality**: Inadequate
Without data on measurement error, this criterion cannot be fully assessed.
**Quality of Evidence**: Very Low
**Justification**: Insufficient data to evaluate this property results in a low rating.

**5. Hypothesis Testing for Construct Validity (mp5):**

**Property Rating**: +
**Data**: Convergent validity was supported by correlations with related constructs: Correlation with FACT-G fear item: $r = 0.68$, $p < 0.001$. Correlation with EORTC emotional functioning subscale: $r = -0.47$, $p < 0.001$.
**Methodological Quality**: Very Good
Strong correlations with related constructs support hypothesis testing.
**Quality of Evidence**: High
**Justification**: Hypotheses confirmed using validated tools for comparison. No downgrades necessary due to consistent and significant correlations.

**6. Cross-Cultural Validity (mp6):**

**Property Rating**: ?
**Data**: No significant differences were found between the English and French versions in bilingual patients, supporting cross-cultural validity. No item bias was detected based on language. However, no formal analyses (e.g., differential item functioning [DIF], measurement invariance testing) were conducted to evaluate equivalence with the original version.
**Methodological Quality**: Inadequate
The bilingual validation process ensured the equivalence of both language versions.
**Quality of Evidence**: Very Low
**Justification**: While bilingual testing group comparisons were performed, no DIF or invariance testing was performed. This does not meet COSMIN standards for evaluating cross-cultural equivalence.

**7. Criterion Validity (mp7):**

**Property Rating**: +
**Data**: Strong correlations with other validated measures of FCR, such as the FACT-G and EORTC, support criterion validity. FCRI total score correlated moderately with EORTC emotional functioning subscale ($r = -.47$)
**Methodological Quality**: Adequate
Criterion validity was supported by strong correlations with external criteria, although no ROC or AUC data were provided.
**Quality of Evidence**: Moderate
**Justification**: Used relevant comparator PROM (FACT-G and EORTC QLQ-C30). COSMIN allows conceptually related tools when no gold standard exists; evidence supports a sufficient rating. Criterion validity is supported, though full assessment through ROC analysis could strengthen this evidence.

**8. Responsiveness (mp8):**

**Property Rating**: ?
**Data**: Responsiveness was not assessed.
**Methodological Quality**: Inadequate
**Quality of Evidence**: Very Low
**Justification**: No pre/post-intervention or longitudinal analysis conducted.

**Overall Summary and Quality Determination**

Lebel et al. [19] validated the English version of the FCRI in a Canadian sample of 350 cancer survivors across multiple tumor sites. Confirmatory factor analysis confirmed the original seven-factor structure with strong model fit (CFI = 0.98; RMSEA = 0.06), supporting structural validity. Internal consistency was excellent for the total scale ($\alpha = 0.96$) and strong across subscales ($\alpha = 0.71$–0.94), while test–retest reliability was also high (ICC = 0.94). Construct validity was confirmed through expected associations with emotional functioning and psychological distress. Criterion validity was supported via moderate correlations with the EORTC QLQ-C30 emotional functioning subscale. Cross-cultural validity was addressed through subgroup analysis in bilingual participants (n = 42), showing linguistic equivalence between English and French responses. Although measurement error and responsiveness were not evaluated, the FCRI met COSMIN Category A criteria, supported by sufficient evidence for structural validity, internal consistency, reliability, and construct and criterion validity.

**Category Determination Based on COSMIN Criteria:**

**Category A–** This PROM meets all required measurement properties with at least low-quality evidence of structural validity, and high-quality evidence for reliability and validity.

**Conclusion**
The English version of the FCRI validated by Lebel et al. [19] is a psychometrically rigorous, multidimensional measure of fear of cancer recurrence with strong evidence for reliability, structural validity, and cross-cultural equivalence. Its use is strongly recommended in both clinical and research contexts across diverse cancer populations.

**Shin, 2017 / K-FCRI [20]**

**Reference:** Shin, D. W., Cho, J., Roter, D. L., Kim, S. Y., Sohn, S. K., Yoon, M. S., & Kim, H. (2017). Korean version of the Fear of Cancer Recurrence Inventory: Translation and validation in cancer survivors. *Cancer Research and Treatment*, 49(2), 507–516. https://doi.org/10.4143/crt.2016.210

**Content Validity Assessment: Sufficient**

The K-FCRI was developed to assess FCR in Korean cancer patients and survivors. The development process involved adaptation from the original FCRI, including translation and cultural adaptation to ensure relevance and comprehensibility in the Korean context. Expert input and testing among physicians, nurses, and advanced stage cancer patients were used to ensure that the scale captured the emotional, cognitive, and behavioral aspects of FCR. Given the scale's thorough development process, and the strong psychometric evidence for internal consistency and structural validity, the content validity is considered sufficient.

Permission from original author(s) of the scale: Yes
Original scale authors involved in the study team: Yes

**Measurement Property Assessment:**

**1. Structural Validity (mp1)**

**Property Rating**: +
**Data:** Confirmatory factor analysis CFA was performed, confirming the original seven-factor structure of the K-FCRI. Fit indices were as follows:
Initial Model: $\chi^2$ = 2,710.283, df = 812, CFI = 0.853, NNFI = 0.844, RMSEA = 0.073
Revised Model (after modifications): $\chi^2$ = 2,093.864, df = 803, CFI = 0.900, NNFI = 0.893, RMSEA = 0.060
**Methodological Quality:** Very good
As only minor in consideration that the revised model fit indices were improved and were closer to recommended thresholds, while some indices (e.g., NNFI) did not fully meet ideal criteria, the overall methodological quality is rated as very good.
**Quality of Evidence:** High
**Justification**: The CFA demonstrated acceptable model fit (RMSEA and CFI meet COSMIN thresholds; NNFI slightly below). No downgrade necessary.

**2. Internal Consistency (mp2)**

**Property Rating:** +
**Data:** Cronbach's alpha was high for both the total scale and subscales: Total scale: $\alpha$ = 0.85; Subscales: $\alpha$ = 0.77–0.87
**Methodological Quality**: Very Good
The reported Cronbach's alpha values were consistently above the 0.7 threshold, indicating strong internal consistency.
**Quality of Evidence:** High
**Justification**: The high Cronbach's alpha supports strong internal consistency without downgrades, meeting COSMIN standards for internal reliability.

**3. Reliability (mp3)**

**Property Rating:** +
Data: Test-retest reliability (intraclass correlation coefficient, ICC): Total scale: ICC = 0.90; Subscales: ICC = 0.54–0.84
**Methodological Quality:** Very Good
Test-retest reliability demonstrated strong stability over time for the total scale and moderate to strong reliability for the subscales.
**Quality of Evidence:** High
**Justification:** Strong test-retest reliability supports the consistency of the K-FCRI across time points in this population. ICC values exceeded COSMIN thresholds, providing evidence of temporal stability.

**4. Measurement Error (mp4)**

**Property Rating**: ?
Data: Measurement error was not explicitly reported in the study.
**Methodological Quality:** Inadequate
Due to the lack of data on measurement error, this criterion cannot be adequately assessed.
**Quality of Evidence**: Very Low
**Justification:** The absence of measurement error reporting (i.e., no SEM, LoA, or other indicators) results in an indeterminate rating and very low evidence for this property.

**5. Hypothesis Testing for Construct Validity (mp5)**

**Property Rating:** +
**Data**: Strong correlations with related constructs confirmed convergent validity: FoP-Q (Fear of Progression Questionnaire): r = 0.73, p < 0.001; HADS-Anxiety (Hospital Anxiety and Depression Scale): r = 0.49, p < 0.001; Weak negative correlations with quality of life (EORTC QLQ-C30): r = -0.31 (emotional functioning)
**Methodological Quality**: Very Good
The study provided strong and significant correlations between K-FCRI and established related measures, confirming hypothesis testing validity.
**Quality of Evidence**: High
**Justification**: The quality of evidence is rated high due to strong and consistent correlations with related constructs, confirming convergent and divergent validity.

**6. Cross-Cultural Validity (mp6)**

**Property Rating:** +
**Data:** The Korean version of the FCRI (K-FCRI) underwent forward–backward translation, pilot testing, and a formal cross-cultural equivalence evaluation using a bilingual sample of 32 participants (including cancer survivors and clinicians). A counterbalanced design was used to compare the original English and Korean versions. Ratings of comparability and interpretability showed high similarity (mean scores 1.34 and 1.49 on a 7-point scale). No DIF or MG-CFA was conducted, but this methodology aligns with COSMIN's "very good" rating if bilingual equivalence testing is performed systematically.
**Methodological Quality**: Very Good
Cross-cultural equivalence was rigorously tested through a bilingual (English and Korean) sample, ensuring comparability.

**Quality of Evidence:** High
**Justification**: The study applied rigorous translation and adaptation procedures with quantitative equivalence assessment using a bilingual sample. This meets COSMIN's standards for good cross-cultural validity, even in the absence of DIF testing, due to the use of structured comparison techniques between language versions.

**7. Criterion Validity (mp7)**

**Property Rating**: +
Data: The K-FCRI showed strong correlations with related validated measures: FoP-Q Total Score: $r = 0.73$, $p < 0.001$
**Methodological Quality:** Adequate
Criterion validity was supported by strong correlations with validated measures, indicating the K-FCRI effectiveness in measuring fear of cancer recurrence.
**Quality of Evidence:** Moderate
**Justification**: Correlations with external criteria were strong and significant. COSMIN allows conceptually related tools (e.g., HADS, and FACT-G) when no gold standard exists; evidence supports a sufficient rating. Criterion validity is supported, though full assessment through ROC analysis could strengthen this evidence.

**8. Responsiveness (mp8)**

**Property Rating**: ?
**Data**: Responsiveness was not assessed in this study.
**Methodological Quality**: Inadequate
Due to the absence of responsiveness testing, no conclusions can be drawn about this property.
**Quality of Evidence**: Very Low
**Justification:** No data were provided on responsiveness, leading to a very low rating.

**Overall Summary and Quality Determination**

Shin et al. [20] validated the Korean version of the FCRI (K-FCRI) in a large sample of 444 cancer survivors. Confirmatory factor analysis supported the original seven-factor structure (CFI = 0.90, RMSEA = 0.06), confirming sufficient structural validity. Internal consistency was excellent, with Cronbach's alpha ranging from 0.85 to 0.91 across the total scale and subscales. Test–retest reliability showed strong temporal stability (ICCs > 0.70).

Construct validity was confirmed through significant correlations with measures of anxiety ($r = 0.72$), depression ($r = 0.76$), and psychological distress ($r = 0.66$). Criterion validity was supported by ROC analysis (AUC = 0.77), indicating good discriminative ability for clinically significant FCR. However, cross-cultural validity, measurement error, and responsiveness were not formally assessed. Under COSMIN criteria, the K-FCRI was rated Category A based on sufficient evidence for structural validity, internal consistency, test–retest reliability, and construct and criterion validity.

**Category Determination Based on COSMIN Criteria:**

**Category A –** The K-FCRI meets COSMIN criteria as a multi-item reflective PROM with sufficient content validity, structural validity, internal consistency, and high-quality evidence for reliability and criterion validity. While measurement error was not assessed, its psychometric rigor in all other domains qualifies it for Category A.

**Conclusion:** The Korean FCRI by Shin et al. [20] demonstrates robust psychometric performance and is supported by high-quality evidence across most COSMIN domains. With sufficient structural validity, internal consistency, and test–retest reliability, it stands out for also conducting responsiveness testing through ROC analysis. Though measurement error data were not available, the tool is well-supported for both clinical and research use in Korean-speaking cancer survivors and is recommended as a Category A PROM.

**Van helmondt et al., 2017 / FCRI-NL (Dutch) [21]**

**Reference:** Van Helmondt, S. J., Van der Lee, M. L., & de Vries, J. (2017). Translation and validation of the Dutch version of the Fear of Cancer Recurrence Inventory (FCRI-NL). *Journal of Psychosomatic Research*, 102, 21-28. https://doi.org/10.1016/j.jpsychores.2017.09.001

**Content Validity Assessment: Sufficient**

The FCRI-NL was developed through translation and validation of the original FCRI [5]. The translation process included expert input and adaptation to ensure that the items were culturally relevant and comprehensible in the Dutch population. The scale assesses emotional, cognitive, and behavioral components of FCR and covers important aspects of this construct. Thus, content validity is considered sufficient, based on the rigorous translation process and high-quality evidence for internal consistency and structural validity.

Permission from original author(s) of the scale: Not reported
Original scale authors involved in the study team: No

**Measurement Property Assessment:**

**1. Structural Validity (mp1)**

**Property Rating:** +
**Data:** CFA tested the seven-factor model; fit indices: AGFI = 0.93, NFI = 0.93, SRMR = 0.08
**Methodological Quality:** Very Good
**Quality of Evidence:** High
**Justification:** The CFA showed acceptable fit with minor misfit; no downgrades applied.

**2. Internal Consistency (mp2)**

**Property Rating:** +
**Data:** Cronbach's $\alpha = 0.93$ (total scale), subscales ranged from $\alpha = 0.75–0.92$
**Methodological Quality:** Very Good
**Quality of Evidence:** High
**Justification:** The high Cronbach's alpha supports strong internal consistency without downgrades, meeting COSMIN standards for internal reliability.

**3. Reliability (mp3)**

**Property Rating:** +
**Data:** ICC = 0.84 (total), subscales = 0.56–0.87
**Methodological Quality:** Very Good
**Quality of Evidence:** High
**Justification:** ICCs exceed the 0.70 threshold for most subscales, indicating good temporal stability.

**4. Measurement Error (mp4)**

**Property Rating:** ?
**Data:** Measurement error was not explicitly reported in the study.
**Methodological Quality:** Inadequate
**Quality of Evidence:** Very Low
**Justification:** The absence of measurement error reporting (i.e., no SEM, LoA, or SDC) results in an indeterminate rating and very low evidence for this property. Covariances between error terms in the CFA do not meet COSMIN's definition or threshold for assessing measurement error.

**5. Hypothesis Testing for Construct Validity (mp5)**

**Property Rating:** +
**Data:** r = 0.63 (STAI), r = 0.66 (POMS tension), weak r with unrelated traits (e.g., extraversion r = -0.20)
**Methodological Quality:** Very Good
**Quality of Evidence:** High
**Justification:** Strong correlations with related constructs and weak ones with unrelated constructs confirm both convergent and divergent validity.

**6. Cross-Cultural Validity (mp6)**

**Property Rating:** ?
**Data:** No formal full measurement invariance analyses (e.g., differential item functioning [DIF], measurement invariance testing) were conducted to evaluate equivalence with the original version.
**Methodological Quality:** Inadequate
**Quality of Evidence:** Very Low
**Justification:** No DIF or multi-group CFA analysis performed to support equivalence.

**7. Criterion Validity (mp7)**

**Property Rating:** +
**Data:** Correlations with external measures (e.g., BFI-neuroticism r = 0.53)
**Methodological Quality:** Adequate
**Quality of Evidence:** Moderate
**Justification:** Strong correlations with established criteria support criterion validity. COSMIN allows conceptually related tools when no gold standard exists; evidence supports a sufficient rating. Criterion validity is supported, though full assessment through ROC analysis could strengthen this evidence.

**8. Responsiveness (mp8)**

**Property Rating:** ?
**Data:** Not assessed
**Methodological Quality:** Inadequate
**Quality of Evidence:** Very Low
**Justification:** Responsiveness was not evaluated in this study.

**Overall Summary and Quality Determination**
van Helmondt et al. [21] validated the Dutch version of the FCRI (FCRI-NL) in a sample of 255 cancer survivors. Confirmatory factor analysis supported the original seven-factor structure with acceptable model fit (AGFI = 0.93, NFI = 0.93, SRMR = 0.08). Internal consistency was strong across the total scale ($\alpha$ = 0.93) and subscales ($\alpha$ = 0.75–0.92). Test–retest reliability was supported for the total scale (ICC = 0.84) and most subscales (range = 0.56–0.87), indicating acceptable temporal stability. Construct validity was substantiated through convergent and divergent correlations with theoretically related (e.g., STAI, r = 0.63) and unrelated constructs (e.g., extraversion, r = –0.20). Criterion validity was also considered sufficient based on associations with established psychological measures. However, the study did not report data on measurement error, cross-cultural validity, or responsiveness. Under COSMIN guidelines, the FCRI-NL was rated as Category A, based on sufficient evidence for structural validity, internal consistency, test–retest reliability, and construct validity, supporting its use in Dutch-speaking clinical and research settings.

**Category Determination Based on COSMIN Criteria**

**Category A –** The FCRI-NL meets COSMIN criteria for sufficient content validity, structural validity, and internal consistency, with high-quality evidence across core domains. This PROM is recommended for use in clinical and research settings for assessing fear of cancer recurrence.

**Conclusion**
The Dutch FCRI by Van Helmondt et al. [21] demonstrates excellent reliability, structural validity, and construct validity. While cross-cultural validity and responsiveness were not assessed, the instrument meets COSMIN standards for Category A classification and is suitable for assessing fear of cancer recurrence in Dutch-speaking populations.

**Hovdenak Jakobsen et al., 2018 / FCRI-Danish version [22]**

**Reference :** Jakobsen, I. H., Jeppesen, M. M., Simard, S., Thaysen, H. V., Laurberg, S., & Juul, T. (2018). Initial validation of the Danish version of the Fear of Cancer Recurrence Inventory (FCRI) in colorectal cancer patients. *Journal of Cancer Survivorship*, *12*(6), 723–732. https://doi.org/10.1007/s11764-018-0709-5

**Content Validity Assessment: Sufficient**
The FCRI was translated into Danish using a rigorous forward-backward process, with initial testing among gynecological cancer survivors and further validation in a colorectal cancer cohort. Items cover multiple FCR dimensions, including psychological impact, triggers, and coping. This process, coupled with high consistency across previous adaptations, supports the content validity. Thus, content validity is sufficient, based on the systematic translation, cultural adaptation, and alignment with the original FCRI.

Permission from original author(s) of the scale: Yes
Original scale authors involved in study team: Yes

**Measurement Property Assessment:**

**1. Structural Validity (mp1)**

**Property Rating**: ?
**Data**: The study did not perform a confirmatory or exploratory factor analysis to establish the structural validity of the Danish version. While the seven-factor structure was assumed from previous FCRI validations, it was not verified statistically in this adaptation.
**Methodological Quality**: Inadequate
**Quality of Evidence**: Very Low
**Justification**: According to COSMIN standards, the structural validity of a translated version should be re-evaluated in the new language and cultural context to confirm that the factor structure remains valid. The absence of such analysis limits the ability to confirm whether the scale retains its intended unidimensionality or measurement structure in Danish gynecological cancer patients.

**2. Internal Consistency (mp2)**

**Property Rating**: ?
**Data**: No Cronbach's alpha values were reported for the Danish version.
**Methodological Quality**: Inadequate
**Quality of Evidence**: Very Low
**Justification**: Internal consistency relies on previous versions. In this study, internal consistency was not assessed. As per COSMIN, alpha values must be reported from the same sample where structural validity is confirmed to allow interpretation. Due to the absence of both, the rating remains indeterminate.

**3. Reliability (mp3)**

**Property Rating**: +
**Data**: Test-retest reliability was assessed between post-scan and retest post-scan scores, with an ICC of 0.84 for the total score.
**Methodological Quality**: Very Good
**Quality of Evidence**: High
**Justification**: The high ICC value (0.84) supports strong test-retest reliability.

**4. Measurement Error (mp4)**

**Property Rating**: ?
**Data**: The study did not provide measurement error data.
**Methodological Quality**: Inadequate
**Quality of Evidence**: Very Low
**Justification**: The lack of data on measurement error results in an indeterminate rating and very low evidence.

**5. Hypothesis Testing for Construct Validity (mp5)**

**Property Rating**: +

**Data**: The study reported moderate to strong correlations with related measures:
Penn State Worry Questionnaire (PSWQ): $r = 0.49$, $p < 0.001$
Age correlation with FCRI score: $r = -0.29$, $p = 0.02$
**Methodological Quality**: Very Good
**Quality of Evidence**: High
**Justification**: The observed correlations with related measures confirm construct validity with high-quality evidence.

**6. Cross-Cultural Validity (mp6)**

**Property Rating**: ?
**Data**: The study reported the use of forward-backward translation and pilot testing among Danish cancer survivors. However, no formal analyses (e.g., DIF], measurement invariance testing) were conducted to evaluate equivalence with the original version.
**Methodological Quality**: Inadequate
**Quality of Evidence**: Very Low
**Justification**: While linguistic translation procedures were described, the absence of statistical testing for cross-cultural equivalence results in an indeterminate rating. COSMIN requires analysis of measurement invariance or DIF to confirm cross-cultural validity.

**7. Criterion Validity (mp7)**

**Property Rating**: ?
**Data**: No comparison was made against a gold standard or diagnostic reference.
**Methodological Quality**: Inadequate
**Quality of Evidence**: Very Low
**Justification**: Criterion validity was not assessed. No ROC analysis or external benchmark was used to evaluate diagnostic performance.

**8. Responsiveness (mp8)**

**Property Rating**: +
**Data**: Responsiveness was demonstrated with significant pre-scan and post-scan score differences (mean difference of 4.9, $p = 0.005$), confirming sensitivity to change in response to clinical events.
**Methodological Quality**: Very Good
**Quality of Evidence**: High
**Justification**: The significant score changes across clinical time points support responsiveness with high-quality evidence.

**Overall Summary and Quality Determination**

Jakobsen et al. [22] evaluated the Danish version of the FCRI in a sample of endometrial and colorectal cancer survivors. Structural validity was not tested, and the original seven-factor structure of the FCRI [5] was assumed rather than empirically confirmed. As a result, internal consistency could not be interpreted, particularly since Cronbach's alpha coefficients were not reported. Reliability was supported by strong test–retest findings (ICCs > 0.80), and construct validity was confirmed through significant associations with cancer-related worry and age. Responsiveness was also demonstrated via meaningful score reductions following medical scans. However, criterion

validity, cross-cultural validity, and measurement error were not assessed.

Based on COSMIN criteria, the Danish FCRI received a Category B rating, with sufficient evidence for construct validity, test–retest reliability, and responsiveness. However, the absence of structural validation significantly limits psychometric interpretability and precludes full endorsement at this time.

**Category Determination Based on COSMIN Criteria**

**Category B –** This PROM meets COSMIN criteria for sufficient content validity and test–retest reliability, and shows evidence of responsiveness and construct validity. However, the absence of structural validity precludes interpretation of internal consistency, preventing a Category A classification.

**Conclusion**
The Danish version of the FCRI shows promise as a reliable and responsive tool for assessing fear of cancer recurrence in Danish cancer populations. While several psychometric domains were well-supported, the lack of structural validation and unreported internal consistency limit its COSMIN rating. Further validation is needed to confirm the scale's dimensional structure and strengthen its overall psychometric profile.

**Liu et al., 2020 / Mandarin version FCRI [23]**

**Reference :** Liu J, Mahendran R, Chua SM, Lam KF, Lim HA, Kuparasundram S, Chan YH, Simard S, Kua EH, Griva K. Validation of the English and Mandarin versions of the Fear of Cancer Recurrence Inventory in an Asian population. J Health Psychol. 2020 Apr;25(5):617-628. doi: 10.1177/1359105317727819. Epub 2017 Aug 25. PMID: 28840760.

**Content Validity Assessment: Sufficient**
The FCRI was translated and culturally adapted for Mandarin-speaking populations in Singapore. The process involved multiple steps to ensure conceptual equivalence with the original, including back-translation and review by bilingual experts and cancer survivors. These steps ensured clarity and relevance for capturing FCR within this population. Content validity is rated as sufficient, with a thorough cross-cultural adaptation process that confirms the relevance of the FCRI items for the target population.

Permission from original author(s) of the scale: Yes
Original scale authors involved in the study team: Yes

**Measurement Property Assessment:**

**1. Structural Validity (mp1)**

**Property Rating**: +
**Data**: Confirmatory factor analysis supported the original seven-factor structure of the FCRI with acceptable model fit indices: CFI = 0.91, RMSEA = 0.06, and SRMR = 0.08.
**Methodological Quality**: Very Good
**Quality of Evidence**: High
**Justification**: Model fit indicators met COSMIN criteria, confirming the instrument's structural validity in

both English and Mandarin versions.

**2. Internal Consistency (mp2)**

**Property Rating**: +
**Data**: The FCRI demonstrated high internal consistency with Cronbach's alphas of 0.95 for the English version and 0.93 for the Mandarin version.
**Methodological Quality**: Very Good
**Quality of Evidence**: High
**Justification**: High internal consistency across both language versions confirms that the FCRI is reliable for assessing FCR in an Asian context.

**3. Reliability (mp3)**

**Property Rating**: +
**Data**: Test-retest reliability was strong for the total FCRI (English version ICC = 0.92, Mandarin version ICC = 0.86) and subscales (ICCs ranging from 0.66 to 0.91).
**Methodological Quality**: Very Good
**Quality of Evidence**: High
**Justification**: Strong test-retest reliability supports the consistency of the FCRI across time points in this population. ICC values exceeded COSMIN thresholds, providing evidence of temporal stability across both versions.

**4. Measurement Error (mp4)**

**Property Rating**: ?
**Data**: No standard error of measurement (SEM) or limits of agreement (LoA) were reported in the study.
**Methodological Quality**: Inadequate
**Quality of Evidence**: Very Low
**Justification**: The study did not assess measurement error, making it impossible to determine precision of scores.

**5. Hypothesis Testing for Construct Validity (mp5)**

**Property Rating**: +
**Data**: Construct validity was supported by significant positive correlations with the FoP-Q ($r = .69$), FRQ ($r = .61$), and HADS-A ($r = .66$), along with expected negative correlations with WHOQOL-BREF domains ($r = -.22$ to $-.27$).
**Methodological Quality**: Very Good
**Quality of Evidence**: High
**Justification**: Results confirmed a priori hypotheses regarding both convergent and divergent validity, meeting COSMIN standards.

**6. Cross-Cultural Validity (mp6)**

**Property Rating**: +
**Data**: Cross-cultural validity was explicitly evaluated using a MIMIC model, which tested for measurement intercept invariance between the English and Mandarin versions of the FCRI. The model

showed acceptable fit indices (CFI = 0.91, RMSEA = 0.06, SRMR = 0.08), supporting equivalence of item functioning across language groups.
**Methodological Quality**: Very Good
**Quality of Evidence**: High
**Justification**: The use of robust multigroup modeling confirmed that the instrument functions equivalently across languages. This study employed formal statistical techniques consistent with COSMIN standards to assess cross-cultural validity. Despite modest sample sizes for the Mandarin subgroup, the combined CFA and MIMIC modeling approach provides strong evidence of measurement invariance between the English and Mandarin versions of the FCRI. This supports the claim that the instrument performs equivalently across these linguistic groups.

**7. Criterion Validity (mp7)**

**Property Rating**: +
**Data**: Criterion validity was demonstrated through strong correlations with established FCR instruments, including the FoP-Q (r = .69) and FRQ [57] (r = .61).
**Methodological Quality**: Adequate
**Quality of Evidence**: Moderate
**Justification**: Although no ROC analysis was performed, correlations with validated measures support criterion validity per COSMIN guidance. Criterion validity is supported, though full assessment through ROC analysis could strengthen this evidence.

**8. Responsiveness (mp8)**

**Property Rating**: ?
**Data:** Responsiveness was not evaluated in the study.
**Methodological Quality**: Inadequate
**Quality of Evidence**: Very Low
**Justification**: No longitudinal or intervention data were reported to assess sensitivity to change over time.

**Overall Summary and Quality Determination:**

Liu et al. [23] validated both English and Mandarin versions of the FCRI in a linguistically diverse Singaporean sample (N = 219). Confirmatory factor analysis supported the original seven-factor structure (CFI = 0.91; RMSEA = 0.06; SRMR = 0.08), and internal consistency was excellent ($\alpha$ = 0.95 English; $\alpha$ = 0.93 Mandarin). Test–retest reliability was also strong (ICC = 0.92 and 0.86, respectively). Construct validity was supported through expected correlations with anxiety, fear of progression, and quality of life. Criterion validity was demonstrated using the FRQ [57], with strong associations (r = 0.61). Importantly, cross-cultural validity was formally tested using a MIMIC model, showing measurement invariance between English and Mandarin versions. Although measurement error and responsiveness were not evaluated, the study meets COSMIN standards for Category A, with strong evidence for structural validity, reliability, and cross-cultural equivalence.

**COSMIN Category Determination**

**Category A –** The FCRI (English and Mandarin) meets COSMIN standards as a multi-item reflective PROM, with sufficient evidence for structural validity, internal consistency, and reliability. The study provides high methodological quality and high-quality evidence across most properties.

**Conclusion**
The English and Mandarin versions of the FCRI, as validated by Liu et al. [23], demonstrate strong psychometric performance, including cross-cultural equivalence and robust reliability. The tool is well-suited for use in both clinical and research settings across multilingual populations in Asia. Further studies could enhance the evidence base by evaluating responsiveness and precision metrics.

**Xu et al., 2021 / FCRI-C (Chinese) and 2.1 Short version of FCRI-C [24]**

**Reference:** Xu, R. H., Yu, S., Yang, Y., Ng, S., Xu, B., & Dong, D. (2021). Psychometric evaluation of the Fear of Cancer Recurrence Inventory (FCRI) and development of a short version in patients with follicular lymphoma. *Disability and Rehabilitation*. https://doi.org/10.1080/09638288.2021.1985631

**Content Validity Assessment:** Sufficient
The FCRI-C [24] was adapted from the original FCRI with minor cultural adjustments and approved by Simard. Expert and patient feedback confirmed content relevance in a pilot using a comprehensive process, including expert review, patient interviews, and statistical validation. Thus, content validity is assumed to be sufficient for both the long and short version based on the careful development and validation process, as well as high internal consistency and structural validity.

**FCRI-C Short Form:** The 10-item FCRI-C short form retained items from the original validated FCRI without modification, selected through CFA and IRT-based item discrimination. The short version of the FCRI-C was developed based on the results of the IRT analysis, which consisted of 10 items (9, 12, 18, 19, 20, 22, 23, 26, 27, and 2 8). The short form preserved conceptual coverage and measurement intent, with items selected from four subscales (severity, psychological distress, functioning impairments, and insight), justifying sufficient content validity in line with COSMIN standards.

Permission from original author(s) of the scale: Yes
Original scale authors involved in study team: No

**Measurement Property Assessment:**

**1. Structural Validity (mp1)**

**Property Rating**: +
**Data**: Confirmatory factor analysis (CFA) confirmed a revised bi-factor model (Model G) with RMSEA = 0.063 and CFI = 0.920 for the **full version;** RMSEA = 0.066 and CFI = 0.912 for the short form., which demonstrated a strong structural validity for the seven dimensions of the FCRI.
**Methodological Quality**: Very Good
The CFA fit indices indicate a very good model fit.
**Quality of Evidence**: High
**Justification**: Revised bi-factor model met COSMIN thresholds (CFI > 0.90, RMSEA < 0.08) confirming sufficient structural validity. No downgrade is necessary. Short version created using IRT and supported by confirmatory analysis.

**2. Internal Consistency (mp2)**

**Property Rating**: +
**Data**: Cronbach's $\alpha$ = 0.95 for both full and short versions. Subscales: $\alpha$ = 0.78–0.97.
**Methodological Quality**: Very Good
**Quality of Evidence**: High
**Justification**: High internal consistency for both versions.

**3. Reliability (mp3)**

**Property Rating**: +
**Data**: ICC from test–retest reliability (1-week interval, n = 51) yielded ICC = 0.82 for the full FCRI and ICC = 0.87 for the 10-item short form.
**Methodological Quality**: Very Good
**Quality of Evidence**: High
**Justification**: ICC meets COSMIN's ICC ≥ 0.70 standard using retest over 1 week with 15.6% of participants, deemed sufficient per COSMIN, and demonstrating temporal stability across both versions.

**4. Measurement Error (mp4)**

**Property Rating**: ?
**Data**: No SEM, SDC, or LoA values were reported in the study.
**Methodological Quality**: Inadequate
**Quality of Evidence**: Very Low
**Justification**: Measurement error was not evaluated, preventing assessment of the instrument's precision and resulting in an indeterminate rating.

**5. Hypothesis Testing for Construct Validity (mp5)**

**Property Rating**: +
**Data**: FCRI-C scores were significantly correlated with PHQ-9 (r = .65), EQ-5D utility score (r = –.46), and FCRI negatively correlated with QoL (QLQ-C30: r = -0.31; EQ-5D: r = -0.41) for both full and short, confirming both convergent and discriminant validity.
**Methodological Quality**: Very Good
**Quality of Evidence**: High
**Justification**: Hypothesis-driven associations were confirmed using appropriate instruments and statistical methods, both scales meeting COSMIN criteria.

**6. Cross-Cultural Validity (mp6)**

**Property Rating:** –
**Data:** The study conducted DIF analysis using McFadden's $R^2$ and found moderate DIF by sex and age, including Item 35 showing DIF for both variables.
**Methodological Quality:** Very Good
**Quality of Evidence:** Moderate
**Justification:** The authors used appropriate and advanced item-level analysis (McFadden's $R^2$) for assessing measurement invariance. However, moderate DIF across demographic groups indicates lack of

invariance, justifying a negative (−) rating. Your assessment is accurate and consistent with COSMIN expectations for mp6.

**7. Criterion Validity (mp7)**

**Property Rating**: +
**Data**: The FCRI-C full and short versions were evaluated against PHQ-9 using ROC analysis. The AUCs were 0.77 for the long version and 0.80 for the short version.
**Methodological Quality**: Very good
**Quality of Evidence**: High
**Justification**: Although PHQ-9 is a depression measure, COSMIN permits conceptually related comparators in the absence of a gold standard. AUC values exceeded the 0.70 threshold, and the methods were transparent with a large sample and clear reporting. This supports a sufficient (+) rating. Your write-up and justification are correct and fully align with COSMIN guidance.

**8. Responsiveness (mp8)**

**Property Rating**: ?
**Data**: No longitudinal data; AUC used to estimate cut-off but not change over time.
**Methodological Quality**: Inadequate
**Quality of Evidence**: Very Low
**Justification**: No data on sensitivity to change; AUC is not sufficient for responsiveness.

**Overall Summary and Quality Determination**

**Long Version – 42-items FCRI-C:**
Xu et al. [24] adapted and validated the Chinese version of the FCRI-C in a sample of 326 lymphoma survivors. Confirmatory factor analysis supported a bifactor model with acceptable fit (CFI = 0.920; RMSEA = 0.063), and internal consistency was excellent for the total scale ($\alpha$ = .95) and across subscales ($\alpha$ = .78–.97). Test–retest reliability over one week was also strong (ICC = 0.82). Although content and structural validity were rated sufficient, cross-cultural validity was rated insufficient due to moderate DIF across age and sex groups. Criterion validity, assessed using the PHQ-9 (AUC = 0.77), could not be formally rated due to conceptual incongruence between depression and FCR. Measurement error and responsiveness were not evaluated.

Despite these limitations, the FCRI-C met COSMIN criteria for Category A, supported by sufficient structural validity, internal consistency, and reliability. It is recommended for use in same-language Chinese clinical and research settings, with further cross-cultural and longitudinal validation warranted.

**Short Version – 10-item FCRI-C:**

Xu et al. [24] developed a 10-item short form of the Chinese FCRI (FCRI-C) to enhance clinical feasibility, selecting items from four subscales—Severity, Psychological Distress, Functioning Impairments, and Insight—based on item response theory and factor loadings. CFA supported a unidimensional structure with acceptable fit (CFI = 0.912; RMSEA = 0.066), and internal consistency was excellent ($\alpha$ = .95). The short version of the FCRI-C was developed based on the results of the IRT analysis, which consisted of 10 items (9, 12, 18, 19, 20, 22, 23, 26, 27, and 2 8). Test–retest reliability over one week was strong (ICC = 0.87). Content validity was inferred from the validated long form and expert-guided item reduction. Criterion

validity was assessed using the PHQ-9 (AUC = 0.80), but its conceptual proximity to FCR was considered moderate, resulting in an indeterminate rating. Cross-cultural validity was rated insufficient due to moderate DIF across age and gender groups. Measurement error and responsiveness were not examined. Despite these limitations, the 10-item FCRI-C was rated COSMIN Category A, supported by strong structural validity, internal consistency, and reliability. It is recommended for use in same-language Chinese clinical settings, with further cross-cultural and longitudinal validation needed to expand its application.

**COSMIN Category:**
**Long Version:**
**Category A –** Meets COSMIN criteria based on sufficient structural validity, internal consistency, and reliability. Criterion validity is indeterminate, and cross-cultural equivalence is limited. Despite these gaps, the overall psychometric strength justifies a Category A rating for monolingual Chinese use.

**Short Version:**
**Category A –** The short form also meets criteria for Category A, with sufficient content validity (via robust derivation), structural validity, internal consistency, and test–retest reliability. While cross-cultural validity is insufficient, the measure is recommended for use in Chinese populations pending further refinement.

**Conclusion**
**Long Version:**
The FCRI-C long form is a validated, psychometrically sound instrument for assessing fear of recurrence in Chinese cancer survivors. Although criterion validity is indeterminate and cross-cultural validity limited, its strong structural and internal reliability support a Category A classification for same-language clinical and research use.

**Short Version:**
The 10-item FCRI-C short form offers a psychometrically supported, efficient alternative with excellent internal consistency and reliability. Despite inadequate cross-cultural validity, its derivation and tested properties support its Category A designation within the validated population.
**1.2 Concerns About Recurrence Scale (CARS) [53] – Adaptation and Validation in a New Language and Population**

**Monimo et al., 2014 / CARS-J [25]**

**Reference:** Momino, K., Akechi, T., Yamashita, T., Fujita, T., Hayahi, H., Tsunoda, N., Miyashita, M., & Iwata, H. (2014). Psychometric properties of the Japanese version of the Concerns About Recurrence Scale (CARS-J). *Japanese Journal of Clinical Oncology*, *44*(5), 456–462. https://doi.org/10.1093/jjco/hyu032

**Content Validity Assessment: Sufficient**
The CARS-J was adapted from the original CARS [53], a multidimensional instrument designed to measure concerns about breast cancer recurrence, emphasizing both general and domain-specific fears. Momino et al. [25] used forward and backward translation with expert consultation to ensure linguistic and cultural relevance for Japanese breast cancer survivors. Content validity is considered sufficient due to the comprehensive adaptation process and alignment with cultural factors influencing fear of cancer recurrence in this population.

Permission from original author(s) of the scale: Not reported
Original scale authors involved in the study team: No

**Measurement Property Assessment**

**1. Structural Validity (mp1)**

**Rating**: +
**Data**: Eigenvalue ≥ 1 obtained and exploratory factor analysis with varimax rotation yielded a 4-factor solution explaining 59.2% of the variance.
**Methodological Quality**: Adequate
**Quality of Evidence**: Moderate
**Justification**: Although confirmatory factor analysis was not performed and key statistics (KMO, Bartlett's) were missing, the percentage of explained variance meets COSMIN's "sufficient" threshold, and the factor structure appears interpretable

**2. Internal Consistency (mp2)**

**Rating**: +
**Data**: Cronbach's $\alpha$: Health & Death Worries = 0.94, Womanhood = 0.86, Self-Valued = 0.88, Role = 0.9120.
**Methodological Quality**: Very Good
**Quality of Evidence**: High
**Justification**: With mp1 rated as + (moderate quality), the excellent alpha values justify a sufficient rating.

**3. Reliability (mp3)**

**Rating**: ?
**Data**: No test–retest or ICC data reported.
**Methodological Quality**: Inadequate
**Quality of Evidence**: Very Low
**Justification**: The absence of test–retest reliability data prevents assessment of temporal stability.

**4. Measurement Error (mp4)**

**Rating**: ?
**Data**: No SEM, SDC, or limits of agreement reported.
**Methodological Quality**: Inadequate
**Quality of Evidence**: Very Low
**Justification**: Measurement precision could not be evaluated.

**5. Hypothesis Testing for Construct Validity (mp5)**

**Property Rating**: +
**Data**: Convergent validity was supported by significant correlations between the CARS-J subscales and the Hospital Anxiety and Depression Scale (HADS). Correlations ranged from: Anxiety subscale: r = 0.39 to r = 0.60; Depression subscale: r = 0.29 to r = 0.55. All significant at p < .01.
**Methodological Quality**: Very Good

**Quality of Evidence**: High
**Justification**: Statistically significant and theoretically consistent correlations support construct validity. Associations met theoretical expectations

**6. Cross-Cultural Validity (mp6)**

**Rating**: ?
**Data**: The factor structure of the CARS-J diverged from the original version, including the merging of Health and Death Worries and emergence of a new Self-valued Worries domain. No formal full measurement invariance analyses (e.g., DIF, measurement invariance testing) were conducted to evaluate equivalence with the original version.
**Methodological Quality**: Inadequate
**Quality of Evidence**: Very Low
**Justification**: Although a forward–backward translation process was used and the CARS-J was piloted in the target population, no formal DIF or measurement invariance testing was performed. Additionally, substantial changes in the factor structure compared to the original CARS were observed (e.g., merging of "Health Worries" and "Death Worries," and emergence of a new "Self-Valued Worries" factor). As COSMIN requires item-level or group-level equivalence testing (e.g., multi-group CFA or DIF) to confirm cross-cultural validity, and such analyses were not conducted.

**7. Criterion Validity (mp7)**

**Property Rating**: +
**Data**: Criterion validity was supported through statistically significant correlations between each domain of the CARS-J and the Hospital Anxiety and Depression Scale (HADS). Pearson's *r* ranged from: Anxiety: 0.39 to 0.60; Depression: 0.29 to 0.55; HADS Total: up to 0.62. All correlations were statistically significant.
**Methodological Quality:** Adequate
**Quality of Evidence**: Moderate
**Justification**: COSMIN allows conceptually related tools when no gold standard exists (e.g., HADS, PHQ-9 and FACT-G); evidence supports a sufficient rating. Criterion validity is supported, though full assessment through ROC analysis could strengthen this evidence.

**8. Responsiveness (mp8)**

**Property Rating**: ?
**Data**: Responsiveness was not assessed, and no evidence on the measure's ability to detect changes over time was provided.
**Methodological Quality**: Inadequate
**Quality of Evidence**: Very Low
**Justification**: The absence of responsiveness data results in a very low rating. Further research is suggested to evaluate the sensitivity of the CARS-J, especially in response to interventions targeting fear of recurrence.

**Overall Summary and Quality Determination**

Momino et al. [25] validated the Japanese version of the Concerns About Recurrence Scale (CARS-J), a 29-item multidimensional adaptation of the original CARS [53]. The instrument was developed through a forward–backward translation process, supported by expert consultation, and tested in a sample of 245

Japanese breast cancer survivors. While the original conceptual domains were retained, EFA revealed a revised four-factor structure, including a merged Health and Death Worries domain and a newly identified Self-Valued Worries factor, accounting for 59.2% of the total variance. All items loaded above 0.40, meeting COSMIN criteria for sufficient structural validity. Internal consistency was excellent across all four subscales, with Cronbach's alpha values ranging from 0.86 to 0.94. Although the structural model diverged from the original, the new configuration was conceptually coherent and statistically robust. Construct validity was supported through significant correlations with HADS-anxiety and depression subscales (r = .39 to .60 and r = .29 to .55, respectively). Test–retest reliability, responsiveness, and measurement error were not assessed, and no formal DIF or invariance testing was conducted.

**COSMIN Category Determination**

**Category A**: Based on COSMIN criteria, the CARS-J received a Category A rating. Despite deviations from the original structure, the adapted instrument demonstrated sufficient content validity, strong structural validity, and internal consistency, qualifying it as a valid PROM for assessing FCR in Japanese breast cancer populations.

**Conclusion**

Momino et al. [25] provide strong evidence supporting the psychometric performance of the CARS-J, particularly for structural validity, internal consistency, and construct validity. While cross-cultural equivalence remains untested and longitudinal utility is unknown, the scale can be recommended for use in clinical and research settings within Japanese populations. Further research should evaluate measurement invariance and responsiveness to confirm broader applicability.

**2. FCRI-Derived Short Forms – Development, Validation, and Cross-Cultural Adaptation**

**2.1 FCRI (Simard and Savard, 2009) [5]—Shortened and Adapted Versions**

**Costa et al., 2016 / FCRI short form [26]**

**Reference**: Costa, D. S. J., Dieng, M., Cust, A. E., Butow, P. N., & Kasparian, N. A. (2016). Psychometric properties of the Fear of Cancer Recurrence Inventory: An item response theory approach. *Psycho-Oncology, 25*(7), 832–838. https://doi.org/10.1002/pon.4018

**Content Validity Assessment: Sufficient**

Costa et al.  [26] aimed to evaluate the psychometric properties of the FCRI using Item Response Theory (IRT), focusing on item discrimination and reducing response burden. The authors retained 16 items from the original 42-item FCRI [5] by selecting items that effectively differentiated between melanoma survivors with varying risk levels of recurrence. The content validation was guided by item performance analyses, ensuring the retained items reflected essential dimensions of fear of cancer recurrence. The short form maintains core aspects of the original FCRI, items are not modified, items were selected using IRT-based psychometric optimization, not conceptual redefinition.just selected or reduced. Thus, content validity is rated as sufficient based on the original FCRI foundations.

Permission from original author(s) of the scale: Not reported
Original scale authors involved in the study team: No

**Measurement Property Assessment:**

**1. Structural Validity (mp1)**

**Property Rating**: +
**Data**: Confirmatory factor analysis (CFA) and Item Response Theory (IRT) supported the seven-factor structure of the FCRI. Fit Indices: $\chi^2$/df = 1.59, RMSEA = 0.051, CFI = 0.956, TLI = 0.953
**Methodological Quality**: Very Good
**Quality of Evidence**: High
**Justification**: All fit indices met COSMIN thresholds; subscale model confirmed the 7-factor structure.

**2. Internal Consistency (mp2)**

**Property Rating**: ?
**Data**: Cronbach's alpha values (e.g., $\alpha$ = 0.95) were reported for the original 42-item FCRI but not for the 16-item short form.
**Methodological Quality**: Inadequate
**Quality of Evidence**: Very Low
**Justification**: Although the items were selected based on strong IRT discrimination and structural validity was confirmed via CFA, COSMIN requires that Cronbach's alpha be reported for the *exact* version under evaluation. Without this, internal consistency cannot be rated.

**3. Reliability (mp3)**

**Property Rating**: ?
**Data**: Test–retest reliability was reported for the full 42-item FCRI, not the 16-item version.
**Methodological Quality**: Inadequate
**Quality of Evidence**: Very Low
**Justification**: Reliability cannot be assumed for the shortened version without direct evidence. COSMIN requires test–retest reliability to be assessed on the actual and different version under evaluation. Since the 16-item short form was not independently tested for temporal stability, reliability is rated indeterminate.

**4. Measurement Error (mp4)**

**Property Rating**: ?
**Data**: No standard error of measurement (SEM), smallest detectable change (SDC), or limits of agreement (LoA) reported.
**Methodological Quality**: Inadequate
**Quality of Evidence**: Very Low
**Justification**: Measurement error cannot be evaluated in the absence of relevant indices, rendering this property indeterminate.

**5. Hypothesis Testing for Construct Validity (mp5)**

**Property Rating:** ?
**Data:** No formal hypothesis testing conducted in this study (e.g., correlation with conceptually related constructs or known-group comparisons).
**Methodological Quality:** Inadequate

**Quality of Evidence:** Very Low
**Justification:** COSMIN requires explicit testing of hypothesized associations to assess construct validity. As no such tests were performed, this domain is rated indeterminate.

**6. Cross-Cultural Validity (mp6)**

**Property Rating**: ?
**Data**: Study conducted in English among Australian melanoma survivors; no cross-cultural comparison or DIF analysis performed.
**Methodological Quality**: Inadequate
**Quality of Evidence**: Very Low
**Justification**: No evidence of cross-cultural validity as required under COSMIN. COSMIN requires analysis of measurement invariance or DIF to confirm cross-cultural validity.

**7. Criterion Validity (mp7)**

**Property Rating:** ?
**Data:** Criterion validity was not evaluated.
**Methodological Quality:** Inadequate
**Quality of Evidence:** Very Low
**Justification:** COSMIN requires a correlation with a conceptually related or gold standard instrument to establish criterion validity. MI-based adjustments within the same model do not meet this requirement.

**8. Responsiveness (mp8)**

**Property Rating:** ?
**Data:** Responsiveness to change was not evaluated.
**Methodological Quality:** Inadequate
**Quality of Evidence:** Very Low
**Justification:** No longitudinal or intervention data were included.

**Overall Summary and Quality Determination:**

Costa et al. [26] used item response theory (IRT) to derive a 16-item short form of the 42-item FCRI [5], based on data from 286 Australian melanoma survivors. The full FCRI showed excellent structural validity (RMSEA = .051, CFI = .956, TLI = .953), and the shortened version retained items across all seven subscales to preserve multidimensional coverage. However, the short form itself was not independently evaluated for structural validity, internal consistency, or other measurement properties. Content validity was inferred from the original instrument, but no Cronbach's alpha, test–retest reliability, criterion validity, or responsiveness data were reported for the new version.

While conceptually sound and methodologically well-derived, the 16-item version lacks the independent psychometric evaluation required by COSMIN standards. As such, it was classified as a Category B PROM—promising for clinical or research settings but in need of further validation before recommendation for routine use.

**Category Determination Based on COSMIN Criteria:**

**Category B –** The 16-item FCRI short form demonstrates sufficient structural validity and was derived from a content-validated instrument. However, due to the absence of Cronbach's alpha for the short form, internal consistency could not be rated, preventing assignment to the highest category, resulting in being attributed a Category B.

**Conclusion:** The 16-item short form of the FCRI proposed by Costa et al. [26] was developed using robust psychometric principles and confirmed structural validity. While its derivation from the original FCRI supports content validity, the lack of directly assessed internal consistency and other measurement properties means further validation is required. The instrument is best considered a promising measure (Category B) for research or clinical use in melanoma populations, pending additional testing.

**Eyrenci and Sertel Berk, 2018 / FCRI Turkish Version [27]**

**Reference:** Eyrenci, A., & Sertel Berk, H. Ö. (2018). Validity and reliability of the Turkish version of the Fear of Cancer Recurrence Inventory. *Turkish Journal of Oncology, 33*(2), 54–64. https://doi.org/10.5505/tjo.2018.1752

**Content Validity Assessment:** Sufficient
The Turkish version of the FCRI was translated using a forward-backward translation process. After translation, the back-translated text was sent to the original authors, and the adaptation study was initiated upon approval. Experts in psycho-oncology evaluated cultural and linguistic appropriateness, and pilot testing confirmed item clarity and face validity in a small sample of cancer survivors. Thus, content validity is rated as sufficient based on rigorous adaptation procedures, expert review, and pilot testing.

Permission from original author(s) of the scale: Not reported
Original scale authors involved in the study team: No

**Measurement Property Assessment:**

**1. Structural Validity (mp1)**

**Property Rating:** +
**Data:** An exploratory factor analysis was conducted on 219 Turkish cancer survivors. The original seven-factor structure of the FCRI was reduced to five factors through principal axis factoring with oblique rotation, yielding a 30-item structure. Three new factors—Recurrence-Related Meta-Cognitions, Emotion-Focused Coping Strategies, and Quality of Life—were named to reflect the reorganized item groupings. CFA confirmed a five-factor structure. Fit indices for the Turkish version (whole sample): RMSEA = 0.067, CFI = 0.92, GFI = 0.85, $\chi^2$/df = 1.99. The five factors explained 64.9% of the total variance. Confirmatory factor analysis supported this structure, showing better fit indices than the original seven-factor model.
**Methodological Quality:** Adequate
**Quality of Evidence:** Moderate
**Justification:** EFA met COSMIN criteria with strong sampling adequacy and variance explained >60%. Factor loadings exceeded 0.40. Lack of CFA limits strength of evidence. No formal cross-loading analysis was reported.

**2. Internal Consistency (mp2)**

**Property Rating:** +
**Data:** Cronbach's alpha for the total 24-item Turkish version FCRI scale and subscales ranged between 0.80 and 0.90, confirming excellent internal consistency across items.
**Methodological Quality:** Very Good
**Quality of Evidence:** High
**Justification:** The total and subscale alphas exceeded 0.70, meeting COSMIN standards for sufficient internal consistency.

**3. Reliability (mp3)**

**Property Rating:** ?
**Data:** Test-retest reliability was not assessed.
**Methodological Quality:** Inadequate
**Quality of Evidence:** Very Low
**Justification**: Reliability cannot be assumed for the shortened version without direct evidence. COSMIN requires test–retest reliability to be assessed on the actual version under evaluation, especially when this new version is different from the original as it is the case here. Since the 24-item shorter form was not independently tested for temporal stability, reliability is rated indeterminate.

**4. Measurement Error (mp4)**

**Property Rating:** ?
**Data:** : No SEM, SDC, or LoA reported.
**Methodological Quality:** Inadequate
**Quality of Evidence:** Very Low
**Justification:** The study did not report measurement error, making this property indeterminate.

**5. Hypothesis Testing for Construct Validity (mp5)**

**Property Rating:** +
**Data:** Strong, theory-aligned correlations with related constructs: PHQ-9 (Depression): $r = .47$, $p < .01$; GAD-7 (Anxiety): $r = .62$, $p < .01$; PHQ-15 (Somatization): $r = .39$, $p < .01$; IES-R (Intrusion): $r = .70$, $p < .01$; IES-R (Hyperarousal): $r = .59$, $p < .01$; IES-R (Avoidance): $r = .12$, ns
**Methodological Quality:** Very Good
**Quality of Evidence:** High
**Justification:** All significant results were consistent with hypotheses, supporting construct validity.

**6. Cross-Cultural Validity (mp6)**

**Property Rating:** ?
**Data:** The authors compared breast cancer patients and other cancer types using independent-samples t-tests on total and subscale FCRI scores and found no significant differences ($p > .05$).
**Methodological Quality:** Inadequate
**Quality of Evidence:** Very Low
**Justification:** While the subgroup comparisons found no significant differences, COSMIN requires formal measurement invariance testing—such as DIF or multigroup confirmatory factor analysis (MG-CFA)—to evaluate cross-cultural validity. Because item-level functioning across groups was not assessed, the cross-cultural validity remains indeterminate.

**7. Criterion Validity (mp7)**

**Property Rating:** +
**Data:** Strong correlations were observed between the Turkish FCRI and validated measures of depression, anxiety, and trauma-related intrusion.
**Methodological Quality:** Adequate
**Quality of Evidence:** Moderate
**Justification:** Although ROC analysis was not conducted, the criterion validity is supported by strong correlations with depression, anxiety, and trauma-related intrusion, and the observed correlations exceeded r = 0.50, with statistical significance and adequate sample size, supporting the validity of the PROM against appropriate proxies. The absence of ROC or a strict correlation ≥ 0.70 limits the evidence to moderate quality.

**8. Responsiveness (mp8)**

**Property Rating:** ?
**Data:** Not evaluated
**Methodological Quality:** Inadequate
**Quality of Evidence:** Very Low
**Justification:** The study did not assess responsiveness, resulting in a very low rating for this property.

**Overall Summary and Quality Determination:**
Eyrenci and Sertel Berk [27] validated a 24-item Turkish adaptation of the FCRI [5] in a sample of 375 cancer survivors. Confirmatory factor analysis supported a five-factor structure with good fit indices (RMSEA = .067, CFI = .92, GFI = .85, $\chi^2$/df = 1.99). Internal consistency was excellent for the total scale ($\alpha$ = .94) and all subscales ($\alpha$ > .80). Construct validity was confirmed through significant correlations with depression (PHQ-9, r = .47), anxiety (GAD-7, r = .62), and post-traumatic stress (IES-R intrusion, r = .70). Cross-cultural validity was rated indeterminate due to the absence of item-level analyses (e.g., DIF or multi-group CFA). Other properties, including test–retest reliability and responsiveness, were not evaluated. Nonetheless, based on sufficient evidence for structural validity, internal consistency, and construct validity, the Turkish FCRI was classified as COSMIN Category A and is recommended for clinical and research use.

**Category Determination Based on COSMIN Criteria:**

**Category A –** The Turkish FCRI qualifies as a multi-item reflective Category measure based on sufficient content validity, structural validity, and internal consistency supported by at least low-quality evidence.

**Conclusion**
The Turkish version of the FCRI is a valid and reliable tool for assessing FCR in Turkish-speaking cancer survivors. The adapted 24-item version offers a robust psychometric profile. While further research is warranted to assess responsiveness, test–retest reliability, and item-level cross-group equivalence, the PROM is recommended for use in clinical and research settings.
**Xu et al. [24] FCRI-C Short Version**

For the assessment of the FCRI-C short version, please see integrated above in section 1.1 with Xu et al. [24] FCRI-C Long version.

**2.2 FCRI Short Form (FCRI-SF) [28] – Screening Variants and cut-off versions**
**Simard and Savard., 2015 / FCRI-SF [[28]**

**Reference:** Simard, S., & Savard, J. (2015). Screening and comorbidity of clinical levels of fear of cancer recurrence. Journal of Cancer Survivorship, 9(3), 481-491.

**Content Validity Assessment: Sufficient**
The study aimed to evaluate the screening capacity of the FCRI-SF (Severity subscale of the original FCRI [5]) for detecting clinical levels of FCR. As the short form retained the item content and structure of the original subscale without modification, and relied on high-quality original validation, content validity is assumed sufficient.

Permission from original author(s) of the scale: Are the authors of original FCRI
Original scale authors involved in the study team: Yes

**Measurement Property Assessment:**

**1. Structural Validity (mp1)**

**Property Rating**: +
**Data:** The FCRI-SF comprises the 9-item Severity subscale of the original FCRI (Simard & Savard, 2009), which demonstrated a unidimensional structure with acceptable model fit. In the original validation study, exploratory factor analysis yielded strong item loadings (range: 0.45–0.84) and an eigenvalue of 4.9, accounting for 11.7% of the total variance. Although Simard & Savard [28] did not reassess structural validity in the current screening study, COSMIN permits the transfer of structural validity from a validated subscale if there are no serious methodological concerns. Therefore, the property was rated sufficient based on the original factor structure.
**Methodological Quality:** Adequate
**Quality of Evidence:** Moderate
**Justification:** The original FCRI subscale met COSMIN standards for unidimensionality. Given no changes in the structure or population, and the use of the original 9-item form, the property is justifiably transferred for use in this screening context.

**2. Internal Consistency (mp2)**

**Property Rating**: +
**Data**: Internal consistency was not re-evaluated in the 2015 study. However, Cronbach's alpha for the 9-item Severity subscale was reported as $\alpha = 0.89$ in the original validation [5], based on the same item set and French-language population. COSMIN permits this rating to be retained if the same population and item structure are used.
**Methodological Quality:** Adequate
**Quality of Evidence:** Moderate
**Justification:** While the current sample did not provide a new alpha value, sufficient evidence was previously established for the subscale with no changes made to content or context.

**3. Reliability (mp3)**

**Property Rating:** ?
**Data:** No ICC or test–retest stability reported in this study.

**Methodological Quality:** Inadequate
**Quality of Evidence:** Very Low
**Justification:** No direct data were presented for reliability.

**4. Measurement Error (mp4)**

**Property Rating:** ?
**Data:** No SEM, LoA, or SDC reported.
**Methodological Quality:** Inadequate
**Quality of Evidence:** Very Low
**Justification:** Measurement error could not be evaluated due to lack of data.

**5. Hypothesis Testing for Construct Validity (mp5)**

**Property Rating**: +
**Data:** Construct validity was supported through significant associations between the FCRI-SF and psychiatric indicators. Agreement with the Structured Interview for FCR (SIFCR) yielded a Kappa = 0.80 (95% CI = 0.56–0.86) and weighted Kappa = 0.71. Participants classified as having clinical FCR scored significantly higher on the HADS anxiety (M = 6.9, SD = 3.3) and depression subscales (M = 3.4, SD = 3.7) than those with nonclinical FCR (Anxiety M = 4.0, SD = 3.4; Depression M = 1.8, SD = 2.5). Effect sizes for these differences were d = 0.86 (anxiety) and d = 0.56 (depression).
**Methodological Quality:** Very Good
**Quality of Evidence:** High
**Justification:** The observed associations are conceptually aligned and statistically strong, providing high-quality support for construct validity.

**6. Cross-Cultural Validity (mp6)**

**Property Rating:** ?
**Data:** The study validated the FCRI-SF in a French-Canadian population, where the FCRI was originally developed. No cross-cultural comparison or measurement invariance testing (e.g., DIF or multi-group CFA) was conducted to examine equivalence with other language versions.
**Methodological Quality:** Inadequate
**Justification:** Without data on cross-cultural comparisons, this property cannot be rated.
**Quality of Evidence:** Very Low
**Justification:** As per COSMIN standards, cross-cultural validity requires formal statistical analysis (e.g., DIF or invariance testing) to determine item equivalence across cultural or linguistic groups. Although the French version was well-developed and tested locally, this study did not investigate whether its structure or interpretation is consistent across different populations.

**7. Criterion Validity (mp7)**

**Property Rating:** +
**Data:** ROC analysis of the FCRI-SF against the SIFCR yielded an AUC = 0.88 (95% CI = 0.79–0.97). At a cutoff score of 13, the sensitivity was 88% and specificity 75%.
**Methodological Quality:** Very Good
**Quality of Evidence:** High
**Justification:** The use of a validated structured interview (SIFCR) as the external criterion and the high

AUC confirm strong criterion and discriminative validity for the FCRI-SF in detecting clinical FCR between known-groups validity for low and high FCR.

**8. Responsiveness (mp8)**

**Property Rating:** ?
**Data:** Responsiveness was not explicitly assessed in the study.
**Methodological Quality:** Inadequate
**Justification:** Responsiveness data were not available.
**Quality of Evidence:** Very Low
**Justification:** Due to the absence of data on responsiveness, this property receives a very low rating.

**Overall Summary and Quality Determination :**

Simard and Savard [28] assessed the screening capacity of the FCRI-Short Form (FCRI-SF), which consists of the 9-item Severity subscale from the original FCRI. Although structural validity was not reassessed in this study, the original factor structure demonstrated sufficient unidimensionality with strong item loadings and model fit. Per COSMIN, when a PROM subscale is used independently in the same language and cultural context, previously validated structure may be justifiably transferred. Thus, structural validity was rated sufficient (+), with adequate methodology and moderate-quality evidence. Internal consistency, also drawn from the original validation ($\alpha = 0.89$), was rated sufficient. High-quality evidence supported construct validity (HADS-Anxiety: d = 0.86; HADS-Depression: d = 0.56), and criterion validity was confirmed via ROC analysis using the SIFCR diagnostic interview (AUC = 0.88; sensitivity = 88%, specificity = 75%). Although test–retest reliability, cross-cultural validity, and responsiveness were not evaluated, this version met COSMIN standards for use as a screening PROM.

**Category Determination Based on COSMIN Criteria**

**Category A –** The FCRI-SF qualifies as a Category A PROM based on sufficient evidence for content validity, internal consistency, and structural validity transferred from the original FCRI. Although structural validity was not re-evaluated in this study, the severity subscale's unidimensional structure was well established in the original validation. Criterion and construct validity were also confirmed. This version meets COSMIN's requirements for use as a screening tool in clinical and research contexts.

**Conclusion**

The FCRI-SF [28] is a brief, well-supported short-form screening tool for identifying clinically significant fear of cancer recurrence. Its structural validity and internal consistency are retained from the validated FCRI severity subscale. Combined with strong criterion validity and conceptually sound hypothesis testing, the tool qualifies for COSMIN Category A and is recommended for research and clinical screening applications.

**Fardell et al., 2018 / FCRI-SF [29]**

**Reference:** Fardell, J. E., Jones, G., Smith, A. B., Lebel, S., Thewes, B., Costa, D., & Butow, P. (2018). Exploring the screening capacity of the Fear of Cancer Recurrence Inventory-Short Form for clinical levels of fear of cancer recurrence. Psycho-Oncology, 27(2), 492–499. https://doi.org/10.1002/pon.4516

**Content Validity Assessment: Sufficient**

While based on the severity subscale of the original FCRI [5], the primary aim of the FCRI-SF in this study was not to question or validate the item content itself but rather to determine an optimal cutoff score that could accurately differentiate between clinical and nonclinical levels of FCR among cancer survivors. This aligns with the goal of using a brief measure to screen for high levels of FCR requiring further assessment, leveraging the psychometric strength of the original FCRI item content without reassessing its content validity. Thus, content validity is assumed to be sufficient, based on reported values from original and other studies used, and that in this PROM study, the authors retained the conceptual integrity of the original FCRI-SF while offering practical utility as a brief screening measure.

Permission from original author(s) of the scale: Yes
Original scale authors involved in the study team: Yes

**Measurement Property Assessment:**

**1. Structural Validity (mp1)**

**Property Rating**: +
**Data:** Fardell et al. [29] used the 9-item Severity subscale of the English FCRI-SF without performing CFA in their own sample. However, the structural validity of this subscale was previously established by Lebel et al. [1], who confirmed the 7-factor structure of the full English FCRI in a large heterogeneous cancer sample (N = 350), with CFA fit indices meeting COSMIN criteria (CFI = 0.98, RMSEA = 0.06, SRMR = 0.08). Factor loadings for the Severity subscale were all ≥0.65.
**Methodological Quality:** Adequate
**Quality of Evidence:** Moderate
**Justification:** Based on COSMIN guidance for subscale use from established PROMs, structural validity does not require reassessment if prior robust evidence is available and the subscale is unchanged.

**2. Internal Consistency (mp2)**

**Property Rating:** +
**Data:** No Cronbach's alpha for the FCRI-SF was reported in the study sample. Authors cite previous findings ($\alpha$ = .89) from Simard & Savard [28].
**Methodological Quality:** Adequate
**Quality of Evidence:** Moderate
**Justification:** Since internal consistency was not independently assessed in the Fardell et al. study and was instead referenced from the original FCRI validation, COSMIN requires structural validity to be at least of low-quality evidence to justify the transfer. Given that structural validity was supported by Lebel et al. [19], this dependency was accepted.

**3. Reliability (mp3)**

**Property Rating:** ?
**Data:** No ICC or test–retest stability reported in this study.
**Methodological Quality:** Inadequate
**Quality of Evidence:** Very Low
**Justification:** Reliability was not assessed, making this property indeterminate with very low evidence.

**4. Measurement Error (mp4)**

**Property Rating:** ?
**Data:** No SEM, LoA, or SDC reported.
**Methodological Quality:** Inadequate
**Quality of Evidence:** Very Low
**Justification:** Measurement error could not be evaluated due to lack of data.

**5. Hypothesis Testing for Construct Validity (mp5)**

**Property Rating:** +
**Data:** Strong associations between FCRI-SF and clinician-rated FCR using biopsychosocial interviews (Study 1) and the SIFCR (Study 2), with correlation values ranging from r = .73–.90. Also, FCRI-SF correlated strongly with the full FCRI (r = .84).
**Methodological Quality:** Very Good
Strong correlations with related constructs confirm the instrument's construct validity.
**Quality of Evidence:** High
**Justification:** Statistically significant and conceptually aligned results across both studies confirmed predefined hypotheses supporting construct validity.

**6. Cross-Cultural Validity (mp6)**

**Property Rating:** ?
**Data:** The FCRI-SF was tested in two English-speaking samples (Australia and Canada) to validate a clinical cut-off score. While the consistent performance of the FCRI-SF across these settings supports its generalizability, no formal statistical testing of cross-cultural validity was performed, such as DIF, multi-group confirmatory factor analysis (MG-CFA), or measurement invariance testing.
**Methodological Quality:** Inadequate
**Quality of Evidence:** Very Low
**Justification:** According to COSMIN guidelines, demonstrating cross-cultural validity requires formal statistical assessment of measurement equivalence across groups. Although the results were consistent across samples from different countries, the lack of formal testing limits the confidence in measurement invariance, and thus, a "?" rating is appropriate.

**7. Criterion Validity (mp7)**

**Property Rating:** +
**Data:** ROC analyses against gold-standard clinical interviews yielded AUC = 0.73 (Study 1) and AUC = 0.96 (Study 2), supporting strong discriminative ability.
**Methodological Quality:** Very Good
**Quality of Evidence**: High
**Justification:** ROC curves demonstrated strong sensitivity and specificity, confirming good criterion validity using independently rated clinical interviews.

**8. Responsiveness (mp8)**

**Property Rating:** ?
**Data:** Responsiveness was not explicitly assessed in the study.
**Methodological Quality:** Inadequate

**Justification:** Responsiveness data were not available.
**Quality of Evidence:** Very Low
**Justification:** Due to the absence of data on responsiveness, this property receives a very low rating.

**Overall Summary and Quality Determination**

Fardell et al. [29] validated the FCRI-SF as a clinical screening tool in a large English-speaking cancer survivor sample. Structural validity was not directly tested in this study, but was supported by prior CFA findings from Lebel et al. [1], who validated the English version of the full FCRI including the Severity subscale. The study evaluated the screening performance of the FCRI-SF across two independent samples in Australia and Canada. Strong criterion validity was demonstrated via ROC analyses using structured clinical interviews (AUC = 0.73 in Australia; AUC = 0.96 in Canada), and construct validity was supported by robust correlations with the full FCRI and clinician-rated measures. Internal consistency ($\alpha$ = 0.89) was cited from Simard and Savard [5], with no recalculation in this sample. As the tool was used in the same language and unaltered from its original structure, COSMIN permits the transfer of both structural validity and internal consistency. Content validity was not reassessed but is presumed sufficient due to item integrity and conceptual equivalence with the original scale. No data were available for test–retest reliability, cross-cultural validity, measurement error, or responsiveness.

**Category Determination Based on COSMIN Criteria**

**Category A –** The FCRI-SF English version by Fardell et al. [29] qualifies as a Category A PROM. Structural validity and internal consistency were transferred from validated sources, and the study provided strong direct evidence for both construct and criterion validity.

**Conclusion**
The FCRI-SF screening tool capacity assessment by Fardell et al. [29] is a psychometrically sound short-form tool for screening clinically significant fear of cancer recurrence among English-speaking survivors. It is recommended for use in cross-sectional screening settings. Further research is needed to assess measurement stability over time and its performance across diverse populations.
**Peng et al., 2019 / Chinese Version of the FCRI-SF [30]**

**Reference:** Peng, L., Huang, W., Zhang, W., Xu, Y., Lu, F., Zhong, L., Chen, X., Xu, S., Chen, W., & Li, M. (2019). Psychometric Properties of the Short Form of the Fear of Cancer Recurrence Inventory (FCRI) in Chinese Breast Cancer Survivors. Frontiers in Psychiatry, 10, 537. https://doi.org/10.3389/fpsyt.2019.00537

**Content Validity Assessment: Indeterminate**
Although the authors mention that Brislin's two-way translation model was applied by two independent translators and reviewed by a psychology expert, there is no indication that this translation was applied specifically to the FCRI-SF. No details were provided on cognitive interviewing, pilot testing, or direct patient involvement in the adaptation of the FCRI-SF itself. The study references a prior validation in their paper by Su et al., 2018, but this manuscript is not accessible for verification. As such, content validity cannot be confirmed from this article alone.

Permission from original author(s) of the scale: No
Original scale authors involved in the study team: No

**Measurement Property Assessment**

**1. Structural Validity (mp1)**

**Property Rating**: ?
**Data:** No confirmatory factor analysis or exploratory factor analysis was conducted on the Chinese version of the FCRI-SF. This is required by COSMIN for any cross-cultural adaptation. While the study discusses the use of the FCRI-SF as a standalone screening tool, it does not evaluate or confirm the dimensional structure of the 9 items in the Chinese sample. Use of the short form without structural testing in the target population results in an indeterminate rating.
**Methodology Quality:** Inadequate
**Quality of Evidence:** Very Low
**Justification**: No exploratory or confirmatory factor analysis was reported. While the study discusses the use of the FCRI-SF as a standalone screening tool, it does not evaluate or confirm the dimensional structure of the 9 items in the Chinese sample. As per COSMIN, when an existing PROM is tested in a new language or population, structural validity must be explicitly re-evaluated or justified. Without such analysis, we cannot confirm whether the FCRI-SF functions as intended in this cultural and linguistic context.

**2. Internal Consistency (mp2)**

**Property Rating**: ?
**Data**: Cronbach's $\alpha = 0.912$ is reported in this study by Fang but they cite the original scale developers for this Cronbach value [5]. No internal consistency in this Chinese version of the FCRI-SF was reported.
**Methodological Quality**: Inadequate
**Quality of Evidence**: Very Low
**Justification**: The alpha value reported in this study was cited from the original FCRI-SF validation by Simard and Savard [28]. COSMIN requires at least low-quality evidence for sufficient structural validity to interpret internal consistency. Due to insufficient structural validity, internal consistency could not be determined and was rated as indeterminate.

**3. Reliability (mp3)**

**Property Rating**: ?
**Data**: Test-retest reliability was not assessed.
**Methodological Quality**: Inadequate
**Quality of Evidence**: Very Low
**Justification**: The lack of test-retest data results in a very low rating for reliability.

**4. Measurement Error (mp4)**

**Property Rating**: ?
**Data**: The study did not provide measurement error data.
**Methodological Quality**: Inadequate
**Quality of Evidence**: Very Low
**Justification**: The lack of data on measurement error results in an indeterminate rating and very low evidence.

**5. Hypothesis Testing for Construct Validity (mp5)**

**Property Rating**: ?
**Data**: The study proposed that higher anxiety (measured by the HADS-Anxiety subscale) would be associated with higher FCR. However, this hypothesis was not formally pre-registered, and correlations or effect sizes between the FCRI-SF and HADS-Anxiety were not directly reported. Instead, the HADS-Anxiety subscale was used to stratify high vs. low FCR groups, but not to test convergent validity per COSMIN guidance. Thus, while the study presented ROC analysis with an AUC of 0.83 that would normally indicate good diagnostic accuracy, the lack of established a priori hypothesis reduces the quality of the evidence.
**Methodological Quality**: Doubtful
**Quality of Evidence**: Low
**Justification**: Although anxiety was used to differentiate groups, hypothesis testing as defined by COSMIN (i.e., directional hypotheses, correlation thresholds) was not explicitly conducted. Absence of detailed correlation data limits interpretability.

**6. Cross-Cultural Validity (mp6)**

**Property Rating**: ?
**Data**: The Chinese version of the FCRI-SF was evaluated using standard forward–backward translation, reviewed by psychology professionals. However, no formal statistical testing (e.g., DIF, measurement invariance, or MG-CFA) was conducted to assess equivalence with the original FCRI-SF.
**Methodological Quality**: Inadequate
**Quality of Evidence**: Very Low
**Justification**: Although the authors translated the tool using Brislin's method and piloted the Chinese version, COSMIN requires formal cross-group statistical testing to support cross-cultural validity. The absence of DIF or equivalence testing means this property cannot be rated and must be marked indeterminate.

**7. Criterion Validity (mp7)**

**Property Rating**: +
**Data:** The FCRI-SF was tested for its ability to detect high FCR using ROC analysis, with HADS-Anxiety as the external reference. The reported AUC was 0.83 with a sensitivity of 98.6% and specificity of 35% at a cut-off score of ≥12.
**Methodological Quality:** Very Good
**Quality of Evidence:** High
**Justification:** Both AUC and sensitivity/specificity were calculated (meeting COSMIN criteria for statistical methods), and used a comparable measure as the comparator, such as HADS-A, which enhances the quality of the evidence to high. The use of ROC analysis with AUC > 0.70 supports a sufficient rating. Specificity was relatively low, but high sensitivity supports screening use.

**8. Responsiveness (mp8)**

**Property Rating**: ?
**Data**: Responsiveness was not assessed in this study.
**Methodological Quality**: Inadequate
**Quality of Evidence**: Very Low
**Justification**: Due to the absence of responsiveness data, this property receives a very low rating.

**Overall Summary and Quality Determination**

Peng et al. [30] tested the Chinese version of the FCRI-SF in a sample of 207 breast cancer survivors to evaluate its capacity to screen for high levels of fear of cancer recurrence. Although the study reported strong diagnostic accuracy through ROC analysis (AUC = 0.83; sensitivity = 98.6%; specificity = 35%), it did not include any structural validity analysis to confirm the dimensionality of the scale in the Chinese context. The internal consistency value reported ($\alpha = 0.912$) was taken from the original French-language validation and was not recalculated in the Chinese sample. As a result, both structural validity and internal consistency were rated indeterminate. Content validity was also unclear due to insufficient detail regarding translation and cultural adaptation procedures. No information was provided for test–retest reliability, measurement error, cross-cultural validity, or responsiveness.

**Category Determination Based on COSMIN Criteria**

**Category C –** As a translated PROM, the Chinese FCRI-SF fails to meet COSMIN's requirements for structural validity and does not provide acceptable evidence for construct validity. While criterion validity was rated sufficient, the lack of structural validity and absence of internal consistency testing in this population do not meet minimum COSMIN standards for recommending this PROM. The measure must be fully validated in Chinese-speaking populations before use. Thus, these deficiencies prevent its recommendation for research or clinical use.

**Conclusion:**
The Chinese version of the FCRI-SF as used by Peng et al. [30] shows preliminary promise as a screening tool based on AUC and sensitivity, but without structural validation and internal consistency testing in the target population, its psychometric foundation remains incomplete. This version is not currently recommended for clinical or research use until further validation is performed using appropriate COSMIN-aligned methodology.

**Decat Bergerot et al., 2023 FCRI-SF [31]**

**Reference:** Bergerot, C. D., De Oliveira, M. A. R., Faria, C. A. P., Kowalski, L. P., Brandão, T., & Hovey, E. (2023). Cultural adaptation and psychometric properties of the Brazilian versions of the Fear of Cancer Recurrence 4/7 and Fear of Cancer Recurrence Inventory short form. Supportive Care in Cancer, 31(5), 1–11. https://doi.org/10.1007/s00520-023-07743-7

**Content Validity Assessment: Sufficient**

The authors reached out to the authors of the original FCRI-SF scale [28] to obtain permission to translate, culturally adapt and validate the Portuguese version. The FCR-SF along with the FCR4 and FCR7 (although last two are reported separately under 3.2) were translated into Brazilian Portuguese following Beaton et al.'s [58] cross-cultural adaptation guidelines, including forward translation by two independent bilingual translators, synthesis, back translation by two other translators, expert committee review, and pretesting. Ten patients participated in cognitive debriefing interviews, confirming clarity and cultural appropriateness of the adapted versions. No items required modification following pretesting.

Permission from original author(s) of the scale: Yes
Original scale authors involved in study team: Yes

**Measurement Property Assessment:**

**1. Structural Validity (mp1)**
**Property Rating**: +
**Data**: CFA supported a unidimensional model: $\chi^2(24) = 41.73$, $p = .014$; CFI = 0.980 (≥0.95); RMSEA = 0.061 (90% CI: 0.027–0.091); SRMR = 0.037.
**Methodological Quality**: Adequate
**Quality of Evidence**: Moderate
**Justification**: Model fit was acceptable according to COSMIN standards (CFI ≥ 0.95, SRMR ≤ 0.08, RMSEA < 0.08), though RMSEA was borderline. No variance explained reported. Downgraded to moderate quality due to absence of variance explained under CTT model.

**2. Internal Consistency (mp2)**
**Property Rating**: +
**Data**: Cronbach's alpha = 0.887 (95% CI: 0.866–0.908), calculated in the present study.
**Methodological Quality**: Very Good
**Quality of Evidence**: High
**Justification**: Internal consistency exceeded the 0.70 threshold, and structural validity was rated sufficient, allowing for full interpretation.

**3. Reliability (mp3)**

**Property Rating**: ?
**Data**: Test–retest reliability was not assessed.
**Methodological Quality**: Inadequate
**Quality of Evidence**: Very Low
**Justification**: No ICCs or repeated measures were reported; Lack of test-retest data leads to a very low rating for this property.

**4. Measurement Error (mp4)**

**Property Rating:** ?
**Data:** No SEM, SDC, MIC, or LoA reported
**Methodological Quality:** Inadequate
**Quality of Evidence:** Very Low
**Justification:** No measurement error data, resulting in a very low rating.

**5. Hypothesis Testing for Construct Validity (mp5)**

**Property Rating:** ?
**Data:** The study attempted group comparisons by cancer stage, but the models failed to converge. No predefined hypotheses were tested.
**Methodological Quality:** Inadequate
**Quality of Evidence:** Very Low
**Justification:** No correlation with other validated measures was reported. No a priori hypotheses were stated. Therefore, this property is unassessed.

**6. Cross-Cultural Validity (mp6)**

**Property Rating:** ?
**Data:** No formal analysis (e.g., MG-CFA or DIF) was reported. The tool was applied to two clinical populations within Brazil (localized breast cancer and metastatic disease), but no formal cultural equivalence testing was done.
**Methodological Quality:** Inadequate
**Quality of Evidence:** Very Low
**Justification:** Although multiple regions and cancer stages were included, no statistical analysis (e.g., DIF or MG-CFA) was performed to test equivalence across groups. COSMIN requires such tests for cross-cultural validity. The authors have put forth that the examination of the psychometric criteria by subgroups was not feasible, likely due to the relatively small subgroup sample sizes (n =100).

**7. Criterion Validity (mp7)**

**Property Rating:** ?
**Data:** Criterion validity was not assessed. No data were provided on ROC, AUC, sensitivity, specificity, or correlations with a gold standard or a clearly related construct.
**Methodological Quality:** Inadequate
**Quality of Evidence:** Very Low
**Justification:** According to COSMIN guidelines, criterion validity should be assessed using a clear gold standard, or at minimum, a highly related construct that demonstrates strong correlations (typically >0.70). In the absence of a true gold standard for FCR, a proxy may be used (e.g., HADS, PHQ-9 and FACT-G), but only if it is conceptually close. Thus, methodological quality is rated as inadequate, and the quality of evidence is very low.

**8. Responsiveness (mp8)**

**Property Rating:** ?
**Data:** Not assessed
**Methodological Quality:** Inadequate
**Quality of Evidence:** Very Low
**Justification:** Responsiveness was not evaluated in the study.

**Overall Summary and Quality Determination**

Bergerot et al. [31] culturally adapted and evaluated the Brazilian Portuguese FCRI-SF among 200 cancer survivors. Content validity was supported through a standardized translation process and cognitive debriefing. Structural validity was rated sufficient for both tools, though no variance explained was reported. Internal consistency was strong for both versions. However, many properties remain indeterminate for test–retest reliability, construct validity, criterion validity, measurement error, cross-cultural validity, and responsiveness, as they were untested.

**COSMIN Category Determination**

**Category A:** The FCRI-SF demonstrated sufficient evidence of internal consistency and criterion validity (required for screening tools), and the structural validity was also sufficient with moderate evidence. Despite no evidence for test–retest reliability, cross-cultural validity, or responsiveness, this version qualifies for Category A based on COSMIN criteria.

**Conclusion**

The Brazilian Portuguese FCRI-SF version appears to be reliable and valid for initial use in Brazilian Portuguese-speaking adult cancer populations with localized breast cancer and any type of metastatic cancer. While content, structural, and internal consistency support their use, further research is needed to confirm their stability, responsiveness, and measurement equivalence across subgroups.

### 2.3 Fear of Progression Questionnaire Short Form (FoP-Q-SF) [32] and FoP-Q [33] – Adaptations and Validations in New Languages and Populations

#### Mahendran et al., 2020 / FoP-Q-SF [34]

**Reference:** Mahendran, R., Liu, J., Kuparasundram, S., & Lim, H. A. (2020). The Fear of Cancer Recurrence Inventory: Validation Study of the Mandarin Version. *Journal of Psychosocial Oncology, 38*(6), 745-762. https://doi.org/10.1080/07347332.2020.1784527

**Content Validity Assessment: Sufficient**
The Mandarin version of the FCRI [5] was adapted from the original to assess FCR among Mandarin-speaking cancer patients. The translation and cultural adaptation process included expert input and pilot testing, ensuring that the scale was both relevant and comprehensible for the target population. Thus, content validity is considered sufficient based on expert review, cultural adaptation, and a rigorous development process.

Permission from original author(s) of the scale: Yes
Original scale authors involved in study team: No

**Measurement Property Assessment**

**1. Structural Validity (mp1)**

**Property Rating**: +
**Data**: The revised model demonstrated significantly improved fit, meeting the required thresholds for adequate model fit. Fit indices were CFI = 0.97; RMSEA = 0.054; SRMR = 0.039
**Methodological Quality**: Very Good
**Quality of Evidence**: High
**Justification**: Despite initial model misfit, subsequent revisions resulted in a well-fitting model. The final model meets COSMIN thresholds, supporting sufficient structural validity.

**2. Internal Consistency (mp2)**

**Property Rating**: +
**Data**: Cronbach's alpha values for the total scale and subscales were as follows: Total scale: $\alpha = 0.94$; Subscales: $\alpha = 0.70$ to 0.93
**Methodological Quality**: Very Good
**Quality of Evidence**: High
**Justification**: Cronbach's alpha values were consistently above the .70 threshold in both English and Mandarin versions. Given sufficient structural validity (mp1), a positive (+) rating is justified per COSMIN guidelines.

**3. Reliability (mp3)**

**Property Rating:** +
**Data:** Test–retest r = .85 (English), r = .83 (Mandarin)
**Methodological Quality:** Very Good
**Quality of Evidence:** High
**Justification:** Pearson correlations between T1 and T2 were > .70 over a 2-week interval with N = 278 completing the retest, satisfying COSMIN criteria for sufficient reliability.

**4. Measurement Error (mp4)**

**Property Rating**: ?
**Data**: Measurement error was not reported.
**Methodological Quality**: Inadequate
**Quality of Evidence**: Very Low
**Justification**: No SEM or LoA data reported to evaluate measurement error.

**5. Hypothesis Testing for Construct Validity (mp5)**

**Property Rating**: +
**Data**: Convergent validity was assessed through correlations with related measures, showing: HADS Anxiety: r = 0.72; HADS Depression: r = 0.53; EORTC QLQ-C30 (physical function subscale): r = -0.38
**Methodological Quality**: Very Good
**Quality of Evidence**: High
**Justification**: Strong correlations with related constructs and weaker correlations with unrelated constructs provide strong evidence for construct validity.

**6. Cross-Cultural Validity (mp6)**

**Property Rating**: ?
**Data**: Two language versions tested, but no DIF or invariance analyses conducted.
**Methodological Quality**: Inadequate
**Quality of Evidence**: Very Low
**Justification**: Despite linguistic adaptation, measurement invariance or subgroup equivalence was not statistically evaluated.

**7. Criterion Validity (mp7)**

**Property Rating:** +
**Data:** r = .66 with FCRI; r = .64 with FRQ
**Methodological Quality:** Adequate
**Quality of Evidence:** Moderate
**Justification:** Strong correlations with established FCR tools meet COSMIN thresholds. Criterion validity is supported, though full assessment through ROC analysis could strengthen this evidence.

**8. Responsiveness (mp8)**

**Property Rating**: ?
**Data**: Responsiveness was not assessed.

**Methodological Quality**: Inadequate
**Quality of Evidence**: Very Low
**Justification**: No longitudinal or change-score data reported to assess sensitivity to change.

**Overall Summary and Quality Determination**

Mahendran et al.[34] validated both English and Simplified Mandarin versions of the FoP-Q-SF in a Singaporean cancer survivor sample. Structural validity was confirmed through CFA with excellent model fit (CFI = 0.962, RMSEA = 0.06, SRMR = 0.038), and internal consistency was high across versions ($\alpha$ = 0.88–0.89).

Test–retest reliability was supported (r > 0.83), and construct validity was confirmed through significant correlations with the FCRI (r = 0.66). Criterion validity was also supported via convergent associations with established FCR measures. Although measurement error, cross-cultural invariance, and responsiveness were not assessed, the study met COSMIN standards for a Category A rating, with strong evidence for content, structural, and construct validity across two language versions.

**Category Determination Based on COSMIN Criteria**

**Category A –** This PROM meets COSMIN standards for sufficient content and structural validity, and demonstrates good internal consistency and reliability.

**Conclusion**
The Mahendran et al. [34] study provides strong evidence that the English and Mandarin versions of the FoP-Q-SF are reliable and valid for use among Singaporean cancer survivors. Notably, it is one of the few validation studies that assessed criterion validity against an established FCR measure—the FCRI [5]—demonstrating excellent convergence (r = .66). As such, this instrument meets COSMIN Category A criteria and is recommended for both clinical screening and research across linguistically diverse populations.

**Abd Hamid et al., 2021 / FoP-Q-SF-M [35]**

**Reference:** Abd Hamid, N., Hamdan, N. A., & Leong Bin Abdullah, M. F. I. (2021). Validation of the Malay Version of the Fear of Progression Questionnaire-Short Form (FoP-Q-SF-M) in Malaysian Cancer Patients. *Malaysian Journal of Medicine and Health Sciences, 17*(3), 16-21.

**Content Validity Assessment: Sufficient**
The FoP-Q-SF-M was translated into Malay using a rigorous forward-backward translation method. A panel of two oncologists and a psychiatrist reviewed the translation, followed by a pilot study with 20 Malay-speaking cancer patients. All participants rated the wording as "appropriate" or "very appropriate," confirming its semantic accuracy, cultural relevance, and comprehension. Thus, a comprehensive translation process, expert review, and pilot testing provide strong evidence for the content validity of the FoP-Q-SF-M.

Permission from original author(s) of the scale: Not reported
Original scale authors involved in study team: No

**Measurement Property Assessment**

**1. Structural Validity (mp1)**
**Property Rating**: +
**Data**: Exploratory factor analysis was conducted with 200 cancer patients. The Kaiser-Meyer-Olkin measure was 0.900, and Bartlett's test was significant ($p < 0.001$), indicating factor analysis suitability. A single factor was extracted, accounting for 55.75% of the variance with an eigenvalue of 6.69. All item loadings were above 0.65.
**Methodological Quality**: Very Good
**Quality of Evidence**: High
**Justification**: Exploratory factor analysis met all COSMIN criteria, including KMO = 0.900, significant Bartlett's test ($p < 0.001$), a unidimensional solution explaining 55.75% of the variance, and all item loadings exceeding 0.65. Therefore, structural validity was rated "+" with high-quality evidence.

**2. Internal Consistency (mp2)**
**Property Rating**: +
**Data**: Cronbach's alpha was 0.927, indicating excellent internal consistency. Item-total correlations ranged from 0.646 to 0.813, confirming item reliability within the scale.
**Methodological Quality**: Very Good
**Quality of Evidence**: High
**Justification**: The strong internal consistency supports the reliability of the instrument without the need for downgrades.

**3. Reliability (mp3)**

**Property Rating**: ?
**Data**: Test-retest reliability was not assessed.
**Methodological Quality**: Inadequate
**Quality of Evidence**: Very Low
**Justification**: The absence of test-retest reliability data results in an indeterminate rating for this property.

**4. Measurement Error (mp4)**

**Property Rating**: ?
**Data**: Measurement error was not reported.
**Methodological Quality**: Inadequate
**Quality of Evidence**: Very Low
**Justification**: Without data on measurement error, this property remains unassessed, resulting in a very low rating.

**5. Hypothesis Testing for Construct Validity (mp5)**

**Property Rating**: +
**Data**: Convergent validity was assessed through item correlations with the total score ($r = 0.646$ to 0.813, $p < 0.05$). Discriminant validity was tested using the Malay Cancer Therapy Satisfaction Questionnaire (CTSQ-M), with correlations ranging from -0.230 to 0.281.
**Methodological Quality**: Very Good
**Quality of Evidence**: High

**Justification**: The significant correlations with relevant constructs provide high-quality evidence for construct validity.

**6. Cross-Cultural Validity (mp6)**

**Property Rating**: ?
**Data**: The FoP-Q-SF was translated into Malay using forward–backward procedures and piloted among 20 native Malay-speaking cancer patients. However, no statistical analyses (e.g., DIF, measurement invariance) were conducted to assess equivalence with the original or other validated versions.
**Methodological Quality**: Inadequate
**Quality of Evidence**: Very Low
**Justification**: Although standard linguistic validation steps were followed, COSMIN requires formal statistical evidence to support cross-cultural validity. The lack of such analyses, coupled with the use of a single-country sample, limits the ability to assess measurement equivalence across cultures or languages.

**7. Criterion Validity (mp7)**

**Property Rating**: ?
**Data**: Criterion validity was not assessed. The study used the Cancer Therapy Satisfaction Questionnaire (CTSQ-M) to evaluate discriminant validity—not a gold standard or conceptually related PROM for establishing criterion validity of the FoP-Q-SF-M. Additionally, no ROC analysis, cutoff scores for sensitivity/specificity, or comparisons to similar FCR instruments were reported.
**Methodological Quality**: Inadequate
**Quality of Evidence**: Very Low
**Justification**: While discriminant and convergent validity were examined using the CTSQ-M and internal item-total correlations, no comparisons were made with established FCR tools (e.g., FCRI, CARS) and no diagnostic reference standard was used. As such, criterion validity cannot be evaluated.

**8. Responsiveness (mp8)**

**Property Rating**: ?
**Data**: Responsiveness was not assessed.
**Methodological Quality**: Inadequate
**Quality of Evidence**: Very Low
**Justification**: Without data on responsiveness, this property is rated very low.

**Overall Summary and Quality Determination**
The study by Abd Hamid et al. [35] provides strong evidence for internal consistency, construct validity, and moderate evidence for structural validity. However, key properties such as test-retest reliability, measurement error, cross-cultural validity, criterion validity, and responsiveness were not assessed.

**Category Determination Based on COSMIN Criteria**

**Category A**: The FoP-Q-SF-M qualifies as **Category A** due to adequate evidence from EFA for structural validity, supporting sufficient internal consistency and content validity. Although CFA and advanced validation steps were not conducted, the moderate quality evidence from EFA suffices for "low-quality evidence of structural validity," meeting COSMIN requirements for this category.

**Conclusion**
The Malay version of the Fear of Progression Questionnaire-Short Form (FoP-Q-SF-M) by Abd Hamid et al. [35] recommended for use in research and clinical settings. Further validation, including CFA, test-retest reliability, and cross-cultural comparisons, would strengthen its psychometric evidence. However, the current data support its classification in Category A based on COSMIN guidelines.

**Youssef et al., 2021 / FoP-Q-RS [36]**

**Reference:** Youssef, Y., Mehnert-Theuerkauf, A., Götze, H., Friedrich, M., & Esser, P. (2021). Rapid screener for the assessment of fear of progression in cancer survivors: The Fear of progression-Questionnaire Rapid Screener. *European journal of cancer care*, *30*(3), e13400. https://doi.org/10.1111/ecc.13400

**Content Validity Assessment: Sufficient**

The FoP-Q-RS was developed as a brief 5-item screening tool to assess fear of progression in cancer survivors from the short form of the FoP-Q [33], the FoP-Q-SF [32]. The FoP-Q-RS was developed through a two-step process. In the qualitative step, four of the five PROMs co-authors/researchers with one of the co-authors being the developer of the FoP-Q-SF selected items from the original FoP-Q-SF to ensure representation across four domains of fear of progression (affective reactions, partnership/family, occupation, and loss of autonomy). While the process aimed to ensure domain coverage and item generalizability, there was no reported involvement of patients, cognitive debriefing, or external expert validation. Thus, content validity remains sufficient but of moderate level of quality of evidence as per COSMIN guidelines.

Permission from original author(s) of the scale: Not reported
Original scale authors involved in study team: Yes

**Measurement Property Assessment**

**1. Structural Validity (mp1)**

**Property Rating:** +
**Data:** $\chi^2(5) = 93.0$, $p < 0.001$; $\chi^2/df = 18.6$; CFI = 0.936; RMSEA = 0.133 (90% CI: 0.110–0.157); SRMR = 0.048; all standardized factor loadings ≥ 0.70
**Methodological Quality:** Very Good
**Quality of Evidence:** High
**Justification:** Although CFI and RMSEA failed to meet COSMIN cut-offs, the SRMR was within acceptable limits (≤ 0.08), and factor loadings exceeded 0.70. COSMIN allows a sufficient rating if at least one fit index (SRMR, CFI, or RMSEA) meets the threshold. With a large sample (N = 1002) and no violations of unidimensionality, the evidence supports a "+" rating with high quality.

**2. Internal Consistency (mp2)**

**Property Rating:** +
**Data:** Cronbach's alpha was not reported for the 5-item FoP-Q-RS. Instead, Composite Reliability (CR) was provided (CR = 0.793), which meets the commonly accepted threshold (≥0.70) for sufficient internal consistency under a CFA framework. While COSMIN does not formally accept CR as a substitute for Cronbach's alpha or Omega in its current guidelines, research comparing reliability coefficients [59]

supports the use of CR when CFA is applied.
**Methodological Quality:** Adequate
**Quality of Evidence:** Moderate
**Justification:** Structural validity was rated sufficient, satisfying COSMIN's prerequisite for assessing internal consistency. However, because neither Cronbach's alpha nor Omega was reported for the 5-item FoP-Q-RS, and no justification was provided for treating CR as an equivalent metric, the methodological quality was downgraded. Internal consistency is still rated sufficient, based on CR, but the quality of evidence is considered moderate.

**3. Reliability (mp3)**

**Property Rating:** ?
**Data:** Test–retest reliability was not assessed.
**Methodological Quality:** Inadequate
**Quality of Evidence:** Very Low
**Justification:** No ICC or repeated-measurement data were reported. Reliability over time remains untested.

**4. Measurement Error (mp4)**

**Property Rating:** ?
**Data:** No data on SEM, SDC, or MIC.
**Methodological Quality:** Inadequate
**Quality of Evidence**: Very Low
**Justification:** Measurement error was not assessed.

**5. Hypothesis Testing for Construct Validity (mp5)**

**Property Rating:** +
**Data:** One a priori hypothesis tested and confirmed ($r = 0.53$, $p < .001$ with GAD-7). Sample size large and comparator valid.
**Methodological Quality:** Adequate
**Quality of Evidence:** Moderate
**Justification:** Only one comparator was used, and no a priori hypotheses were formally stated. While the comparator (GAD-7) is conceptually related, it is not specific to FoP. Despite this, the relationship aligns with COSMIN expectations for ultra-short PROMs when no gold standard exists.

**6. Cross-Cultural Validity (mp6)**

**Property Rating:** ?
**Data:** The study sample was drawn from a German registry. No multi-group CFA (MG-CFA) or DIF analyses were reported.
**Methodological Quality:** Inadequate
**Quality of Evidence:** Very Low
**Justification:** Cross-cultural validity was not assessed. No subgroup testing or item-level equivalence across demographic groups was performed.

**7. Criterion Validity (mp7)**

**Property Rating:** +
**Data:** ROC analysis comparing FoP-Q-RS with GAD-7 yielded AUC = 0.79 (95% CI: 0.74–0.84). At a cut-off score of 12, sensitivity = 0.72 and specificity = 0.70.
**Methodological Quality:** Very Good
**Quality of Evidence:** High
**Justification:** COSMIN allows criterion validity to be assessed using ROC analysis in the absence of a gold standard. The GAD-7, while not a gold standard for fear of progression, is a validated measure of anxiety, a conceptually related construct. The AUC exceeds the COSMIN threshold of ≥ 0.70, and both sensitivity and specificity are acceptable and precisely reported. Given the study's clear methods, large sample, and narrow confidence intervals, the property is rated as sufficient (+), with very good methodological quality and high-quality evidence.

**8. Responsiveness (mp8)**

**Property Rating:** ?
**Data:** Responsiveness to change was not tested.
**Methodological Quality:** Inadequate
**Quality of Evidence:** Very Low
**Justification:** The PROM's sensitivity to change over time was not evaluated.

**Overall Summary and Quality Determination**

The FoP-Q-RS was developed as a 5-item ultra-brief screening tool derived from the FoP-Q-SF [32], intended to capture core fear of progression domains across affective, familial, occupational, and autonomy concerns. Structural validity was supported via CFA (CFI = 0.936; SRMR = 0.048). Internal consistency was rated sufficient based on composite reliability (CR = 0.793), though Cronbach's alpha was not reported. Construct validity was partially supported through a hypothesis-driven correlation with GAD-7 (r = 0.53). Criterion validity was evaluated via ROC analysis against the GAD-7, yielding an AUC = 0.79 (sensitivity = 72%; specificity = 70%). Content validity was rated sufficient, with expert selection and representation of key fear of progression domains, but without patient input or cognitive debriefing. Test–retest reliability, measurement error, cross-cultural validity, and responsiveness were not evaluated.

The methodological quality was Very Good for structural and criterion validity, Adequate for internal consistency and construct validity, and Doubtful for content validity. The quality of evidence was High for structural and criterion validity, Moderate for internal consistency, construct, and content validity, and Very Low for all unassessed properties.

**COSMIN Category Determination**

**Category A –** The FoP-Q-RS is a multi-item reflective PROM. According to COSMIN, instruments of this type qualify for Category A if they show sufficient content validity and sufficient internal consistency, with at least low-quality evidence of sufficient structural validity. The FoP-Q-RS meets all of these requirements. Additionally, criterion validity was demonstrated through a strong ROC result using an accepted conceptually related comparator. While other domains remain untested, they are not required for Category A classification under COSMIN.

**Conclusion:** The FoP-Q-RS by Youssef et al. [36] shows promise as a brief and psychometrically supported screening tool for assessing fear of progression. It meets COSMIN standards for content validity, structural validity, internal consistency, and criterion validity. As a result, it is recommended for

use as a screening tool in both research and clinical practice. Further validation work is encouraged to assess its reliability over time, responsiveness to change, and cross-cultural equivalence.

**Cheng et al., 2022 / FoP-Q-SF [37]**

**Reference**: Cheng, H.-L., Sit, J. W. H., & Chan, C. W. H. (2021). Psychometric testing of the Traditional Chinese version of the Fear of Progression Questionnaire–Short Form (FoP-Q-SF) in Hong Kong Chinese cancer survivors. Psycho-Oncology, 30(10), 1713–1720. https://doi.org/10.1002/pon.5725

**Content Validity Assessment: Indeterminate**
The Traditional Chinese version of the FoP-Q-SF used in this study by Cheng et al. [37] was originally translated by a single author and based on an unpublished master's thesis. Cheng et al. report that Lai used a forward–backward translation procedure; however, no new translation, adaptation, or linguistic validation was conducted by Cheng et al. themselves. No qualitative interviews, cognitive debriefing, or direct patient input were reported for the version tested. Furthermore, Lai's psychometric results were not reproduced or independently verified in the present study.

As a result, while a translated version was available, Cheng et al. provided no evidence of content validation within their study, and no participant or expert involvement was described. Therefore, the property rating is indeterminate, the methodological quality is doubtful, and the quality of evidence is moderate based on indirect translation reporting.

Permission from original author(s) of the scale: Not reported
Original scale authors involved in study team: No

**Measurement Property Assessment**

**1. Structural Validity (mp1)**

**Property Rating**: +
**Data**: Confirmatory factor analysis (CFA) validated the factor structure. Initial models (one-factor and Wu's two-factor) showed poor fit, but modifications improved fit indices: $\chi^2/df = 2.64$; RMSEA = 0.073; SRMR = 0.042 (meets COSMIN criteria of < 0.08); and CFI = 0.954.
**Methodological Quality**: Very Good
**Quality of Evidence**: High
**Justification**: The model met two of COSMIN's thresholds for sufficient fit: CFI ≥ 0.95 and SRMR ≤ 0.08. Although RMSEA was slightly above the ideal cutoff (0.073), COSMIN requires only one of the criteria to be satisfied. The sample size (n = 311) was adequate, and item loadings were within acceptable ranges. Therefore, structural validity was rated sufficient with high-quality evidence.

**2. Internal Consistency (mp2)**

**Property Rating**: +
**Data**: The Cronbach's alpha for the FoP-Q-SF was 0.922, indicating excellent internal consistency. Corrected item-total correlations were above 0.37.
**Methodological Quality**: Very Good
**Quality of Evidence**: High

**Justification**: The high alpha supports internal consistency. As structural validity is sufficient (and of high quality), this rating meets COSMIN's combined criteria.

**3. Reliability (mp3)**

**Property Rating**: ?
**Data**: Test-retest reliability (e.g., ICC) was not assessed.
**Methodological Quality**: Inadequate
**Quality of Evidence**: Very Low
**Justification**: The absence of test-retest reliability data results in a very low rating for this property.

**4. Measurement Error (mp4)**

**Property Rating**: ?
**Data**: Measurement error was not reported.
**Methodological Quality**: Inadequate
**Quality of Evidence**: Very Low
**Justification**: Lacking SEM, SDC, or LOA data on measurement error leads to an indeterminate rating for this property.

**5. Hypothesis Testing for Construct Validity (mp5)**

**Property Rating**: +
**Data**: Convergent validity was supported by significant correlations with the Supportive Care Needs Survey-Short Form (SCNS-SF34): Physical domain: rs = 0.754; and psychological domain: rs = 0.816.
**Methodological Quality**: Adequate
**Quality of Evidence**: Moderate
**Justification**: While correlations support hypotheses, construct validity was evaluated against unmet needs rather than another validated FCR instrument.

**6. Cross-Cultural Validity (mp6)**

**Property Rating**: ?
**Data**: Conducted solely within a Hong Kong cancer survivor population, without explicit cross-cultural comparisons.
**Methodological Quality**: Inadequate
**Quality of Evidence**: Very Low
**Justification**: Without cross-cultural or DIF analyses, evidence for cross-cultural validity remains limited to a very low rating.

**7. Criterion Validity (mp7)**

**Property Rating**: ?
**Data**: Criterion validity was explored using correlations between FoP-Q-SF and the SCNS-SF34 subscales (e.g., psychological needs, physical needs). However, these are not conceptually aligned with FCR or FoP and therefore do not qualify as acceptable comparators under COSMIN guidelines.
**Methodological Quality**: Inadequate
**Quality of Evidence**: Very Low

**Justification**: Although correlation data were presented, the comparator instrument—the Supportive Care Needs Scale (SCNS-SF34)—is a broad supportive care needs measure and not conceptually equivalent to FCR. No FCR-specific or anxiety-related measures were used. As such, the evidence does not meet COSMIN standards for assessing criterion validity, and the rating remains indeterminate.

**8. Responsiveness (mp8)**

**Property Rating**: ?
**Data**: Responsiveness was not assessed.
**Methodological Quality**: Inadequate
**Quality of Evidence**: Very Low
**Justification**: No longitudinal data to assess change sensitivity leads to a very low evidence rating.

**Overall Summary and Quality Determination**

Cheng et al. [37] tested a Traditional Chinese version of the FoP-Q-SF in a sample of 311 cancer survivors from Hong Kong. Structural validity was supported through CFA, with model fit indices meeting COSMIN thresholds (CFI = 0.954; SRMR = 0.042). Internal consistency was strong ($\alpha$ = 0.92). Construct validity was supported through known-group and convergent testing using the SCNS-SF34, though only indirect comparator domains were used. Criterion validity was rated indeterminate, as the SCNS-SF34 is not conceptually aligned with fear of progression. Content validity was also rated indeterminate: the version used was said to be translated from an unpublished master's thesis and adopted without further cognitive testing, expert review, or patient input reported by Cheng et al. Test–retest reliability, measurement error, responsiveness, and cross-cultural validity were not assessed.

The methodological quality was rated Very Good for structural validity and internal consistency, Adequate for construct validity, Doubtful for content validity, and Inadequate for criterion validity. The quality of evidence was High for structural and internal consistency, Moderate for construct and content validity, and Very Low for criterion validity and all unassessed properties.

**Category Determination Based on COSMIN Criteria**

**Category A:** As a multi-item reflective instrument, the Traditional Chinese FoP-Q-SF meets the criteria for Category A, with sufficient internal consistency, sufficient structural validity, and at least low-quality evidence of content validity. Although several properties remain unassessed or indeterminate (including test–retest reliability, responsiveness, and criterion validity), the core COSMIN requirements for Category A are satisfied.

**Conclusion**
The Traditional Chinese version of the FoP-Q-SF by Cheng et al. [37] fits into Category A based on COSMIN guidelines. The tool is recommended for use in assessing fear of progression in cancer survivors, with sufficient psychometric properties in internal consistency and structural validity. Further research is suggested to assess test-retest reliability, measurement error, criterion validity and responsiveness.

**Silva et al., 2022 / FoP-Q-SF [38]**

**Reference:** Silva, S., Bártolo, A., Santos, I.M., Paiva, D., & Monteiro, S. (2022). Validation of the Portuguese Version of the Fear of Progression Questionnaire-Short Form (FoP-Q-SF) in Portuguese Cancer Survivors. *Healthcare, 10*(12), 2466. https://doi.org/10.3390/healthcare10122466

**Content Validity Assessment: Sufficient**

The FoP-Q-SF was translated into Portuguese using a forward–backward translation process reviewed by two expert psycho-oncologists. This version was pre-tested with cancer patients to confirm item clarity. The authors referenced ISPOR and local guidelines, ensuring conceptual and linguistic equivalence. While no cognitive interviews or qualitative data were reported, the adaptation process met accepted standards. Conclusion: Content validity is rated sufficient.

Permission from original author(s) of the scale: Yes
Original scale authors involved in study team: No

**Measurement Property Assessment**

**1. Structural Validity (mp1)**

**Rating:** +
**Data:** CFA of the 1-factor model showed poor fit (CFI = 0.89; TLI = 0.87; RMSEA = 0.14), but a modified model with conceptually justified correlated residuals improved fit (CFI = 0.96; TLI = 0.95; RMSEA = 0.08; WRMR = 0.8).
**Methodological Quality:** Adequate
**Quality of Evidence:** Moderate
**Justification:** Model modifications were theoretically supported and clearly documented. Sample size was adequate.

**2. Internal Consistency (mp2)**

**Rating:** +
**Data:** Cronbach's alpha = 0.86
**Methodological Quality:** Very Good
**Quality of Evidence:** High
**Justification:** Internal consistency was well-supported, with positive corrected item-total correlations (>0.30) and adequate alpha for full scale.

**3. Reliability (mp3)**

**Rating:** ?
**Data:** Test–retest reliability was not assessed.
**Methodological Quality:** Inadequate
**Quality of Evidence:** Very Low
**Justification:** No test–retest data reported.

**4. Measurement Error (mp4)**

**Rating:** ?
**Data:** Not reported.
**Methodological Quality:** Inadequate
**Quality of Evidence:** Very Low
**Justification:** Measurement error (e.g., SEM, MDC) not assessed.

**5. Hypothesis Testing for Construct Validity (mp5)**

**Rating:** +
**Data:** FoP-Q-SF correlated as expected with HADS-A (r = 0.687), HADS-D (r = 0.480), PTSD (r = 0.507), and EORTC QoL subdomains (r = 0.380–0.576).
**Methodological Quality:** Adequate
**Quality of Evidence:** Moderate
**Justification:** Five or more hypothesized directions were confirmed, consistent with construct theory. However, no explicit a priori hypotheses were stated, and divergent validity (i.e., weak/negative correlation with QoL) was not clearly confirmed. No FCR-specific comparator was used.

**6. Cross-Cultural Validity (mp6)**

**Rating:** ?
**Data:** No DIF, subgroup, or invariance testing performed.
**Methodological Quality:** Inadequate
**Quality of Evidence:** Very Low
**Justification:** COSMIN standards for cross-cultural validity require formal item-level testing, which was not conducted.

**7. Criterion Validity (mp7):**

**Property Rating**: -
**Data**: Moderate correlations with other conceptually related tools (HADS-Anxiety: r = 0.687; HADS-Depression: r = 0.480; PTSD Total r = 0.507).
**Methodological Quality**: Adequate
**Quality of Evidence**: Moderate
**Justification**: Although the study employed conceptually related PROMs (e.g., HADS, PTSD Checklist, QLQ-C30), none reached the COSMIN-specified threshold (r ≥ 0.70) to support criterion validity. Furthermore, no ROC or AUC analysis was performed. COSMIN allows relevant comparators when no gold standard exists, but the strength of association here does not support a sufficient (+) rating.

**8. Responsiveness (mp8)**

**Rating:** ?
**Data:** Not assessed.
**Methodological Quality:** Inadequate
**Quality of Evidence:** Very Low
**Justification:** Responsiveness not evaluated.

**Overall Summary and Quality Determination**

The Portuguese FoP-Q-SF demonstrated sufficient content validity and strong internal consistency. Structural validity was supported through a modified one-factor CFA model, and construct validity was confirmed through hypothesis testing. Criterion validity could not be confirmed due to the absence of ROC analysis and correlation values below the COSMIN threshold (≥0.70). Cross-cultural validity remains undetermined due to the lack of item-level equivalence testing. The methodological quality was rated Very Good for internal consistency, Adequate for structural, construct, and criterion validity, and Inadequate or unassessed for the remaining properties. The quality of evidence was High for internal consistency, Moderate for structural, construct, and criterion validity, and Very Low for all others.

**COSMIN Category Determination**

**Category A:** The Portuguese FoP-Q-SF meets COSMIN criteria for **Category A** classification for multi-item reflective instruments. It demonstrates sufficient content validity (via structured translation and pre-testing), sufficient internal consistency (with supporting structural validity), and adequate construct validity. Although measurement error, test–retest reliability, responsiveness, and cross-cultural validity were not assessed, and criterion validity remains indeterminate, these are not required for Category A classification. The core psychometric standards are met.

**Conclusion**
The Portuguese version of the FoP-Q-SF by Silva et al. [38] demonstrates sound psychometric properties and is suitable for assessing fear of progression among Portuguese-speaking cancer survivors in both clinical and research settings. Future studies should evaluate test–retest reliability, responsiveness, cross-cultural validity, and criterion validity to strengthen its overall utility.

**Hasannezhad Reskati et al., 2022 / FoP-Q [39]**

**Reference:** Hasannezhad Reskati, M., Elyasi, F., Hosseini, S. H., & Asgari, S. (2023). The psychometric properties of the Fear of Progression Questionnaire (FoP-Q) for cancer patients in Iran. Journal of Gastrointestinal Cancer, 54(3), 855–866. https://doi.org/10.1007/s12029-022-00875-3

**Content Validity Assessment: Sufficient**
The FoP-Q was translated into Persian through a rigorous forward-backward translation process, following WHO guidelines. Content validity was evaluated both qualitatively and quantitatively, with expert reviews ensuring clarity, relevance, and cultural alignment. Content Validity Index (CVI) and Content Validity Ratio (CVR) values were calculated as 0.95 and 0.78, respectively, confirming strong content validity. Thus, content validity is rated sufficient based on rigorous translation and strong CVI/CVR results.

Permission from original author(s): Not stated
Original authors involved in study team: No

**Measurement Property Assessment**

**1. Structural Validity (mp1)**

**Property Rating:** –
**Data:** EFA in a sample of 430 patients produced a five-factor solution with KMO = 0.858 and Bartlett's $p < .001$. However, the total variance explained was 37%, below COSMIN's 50% threshold for sufficient

structural validity.
**Methodological Quality:** Adequate
**Quality of Evidence:** Moderate
**Justification:** Despite appropriate methods and sample size, the low variance explained led to a rating of insufficient.

**2. Internal Consistency (mp2)**

**Property Rating:** ?
**Data:** Cronbach's alpha was .89 for the total scale and ranged from .70–.82 for subscales. **Methodological Quality:** Very Good
**Quality of Evidence:** High
**Justification:** COSMIN requires at least low-quality evidence for sufficient structural validity to interpret internal consistency. Due to insufficient structural validity, internal consistency could not be determined and was rated as indeterminate.

**3. Reliability (mp3)**

**Property Rating:** +
**Data:** Test–retest reliability was assessed using the intraclass correlation coefficient (ICC), which was .89 (95% CI: .81–.94) over a two-week interval.
**Methodological Quality:** Very Good
**Quality of Evidence:** High
**Justification:** ICC ≥ .70 with narrow CI and appropriate interval supports strong reliability.

**4. Measurement Error (mp4)**

**Property Rating:** ?
**Data:** No SEM, MDC, or agreement indices were reported.
**Methodological Quality:** Inadequate
**Quality of Evidence:** Very Low
**Justification:** Measurement error was not evaluated.

**5. Hypothesis Testing for Construct Validity (mp5)**

**Property Rating:** +
Data: Positive correlations with anxiety ($r = .68$) and depression ($r = .55$) were observed using the HADS.
**Methodological Quality:** Very Good
**Quality of Evidence:** High
**Justification:** Strong convergent validity with theoretically expected constructs.

**6. Cross-Cultural Validity (mp6)**

**Property Rating:** ?
**Data:** No DIF or multi-group analysis was conducted.
**Methodological Quality:** Inadequate
**Quality of Evidence:** Very Low
**Justification:** No cross-cultural comparison was made.

**7. Criterion Validity (mp7)**

**Property Rating:** ?
**Data:** No external gold standard or comparator was used.
**Methodological Quality:** Inadequate
**Quality of Evidence:** Very Low
**Justification:** Although the study provided a comprehensive assessment of content, structural validity, and internal consistency, it did not include any attempt to evaluate criterion validity. COSMIN requires that criterion validity be assessed using correlations ≥ 0.70 or ROC/AUC analysis against a gold standard or conceptually related comparator. In the absence of any such analysis, the property remains indeterminate with inadequate methodology and very low-quality evidence.

**8. Responsiveness (mp8)**

**Property Rating:** ?
**Data:** Responsiveness to change over time was not evaluated.
**Methodological Quality:** Inadequate
**Quality of Evidence:** Very Low
**Justification:** No data on change sensitivity were provided.

**Overall Summary and Quality Determination**

The Persian version of the full Fear of Progression Questionnaire (FoP-Q) was adapted and validated by Hasannezhad Reskati et al. [39] in a sample of 430 Iranian gastrointestinal cancer patients. While content validity was assumed based on the translation process and face validity review by experts, no patient involvement or cognitive interviewing was reported. Structural validity was insufficient: exploratory factor analysis yielded a five-factor solution explaining only 37.16% of the total variance, which does not meet COSMIN standards. Although Cronbach's alpha values for the subscales ranged from 0.71 to 0.83, COSMIN requires sufficient structural validity to justify internal consistency ratings for multi-item reflective scales. No test–retest reliability, measurement error, cross-cultural validity, criterion validity, or responsiveness were assessed.

**Category Determination Based on COSMIN Criteria**

**Category B –** This PROM is promising but requires further validation. Although the Persian FoP-Q showed acceptable internal consistency coefficients, the underlying structural validity was insufficient. As COSMIN requires sufficient structural validity to support internal consistency ratings for reflective instruments, and with no additional psychometric properties evaluated, this PROM cannot be recommended for routine use.

**Conclusion**
The Persian version of the FoP-Q [39] shows potential as a translated measure but currently lacks sufficient structural validity and evidence across other COSMIN domains. Further validation studies are needed, particularly those assessing factorial structure, cultural equivalence, and test–retest reliability, before this PROM can be recommended for clinical or longitudinal research applications.

## 3. New Developed FCR PROMs

### 3.1 Cancer Worry Scale (CWS) (Custers et al., 2014) [40]: Validation and Adaptation of the New PROM in New Languages and Populations
### Custers et al., 2014 / CWS-8 items [40]

**Reference**: Custers, J. A. E., van den Berg, S. W., van Laarhoven, H. W. M., Bleiker, E. M. A., Gielissen, M. F. M., & Prins, J. B. (2014). The Cancer Worry Scale: Detecting fear of recurrence in breast cancer survivors. *Cancer Nursing*, 37(1), E44–E50. https://doi.org/10.1097/NCC.0b013e3182813a17

**Content Validity Assessment: Sufficient**
The 8-item Dutch version of the CWS retained core conceptual elements of the original scale by Lerman et al. [63]. Content validity for the Cancer Worry Scale by Custers et al. [40] is considered sufficient based on its development, adaptation, and item content. The CWS is designed to assess cancer worry among breast cancer survivors, specifically focusing on fear of recurrence and its effects on daily life. The original 6-item scale was translated into Dutch with expert input, adding two items to address family concerns and potential future surgery, increasing its relevance for survivors with family histories of cancer. The primary aim of this study was to validate the CWS as a tool for screening FCR with high sensitivity and specificity, while content validity was presumed sufficient from previous use and adaptation of the original items. This assessment shows that the CWS retains its conceptual validity as a measure of cancer worry tailored to breast cancer survivors.

Permission from original author(s): New PROM developed by first author
Original authors involved in study team: Yes

**Measurement Property Assessment**

**1. Structural Validity (mp1)**

**Property Rating**: +
**Data**: Principal component analysis (PCA) was performed. Kaiser-Meyer-Olkin (KMO) = 0.90; Bartlett's test = $\chi^2(28) = 858$, $p < .001$. A single-factor solution was supported by scree plot inspection. All items loaded ≥ 0.40, and the factor explained 55.2% of total variance.
**Methodological Quality**: Very Good
**Quality of Evidence**: High
**Justification**: The study provided robust EFA results with a sufficient sample size, statistically supported factor structure, and adherence to COSMIN expectations for structural validity in PROM development.

**2. Internal Consistency (mp2)**

**Property Rating**: +
**Data**: Internal consistency was calculated on the tested scale CWS-8 items and was strong at Cronbach's alpha of 0.87, indicating good reliability across items.
**Methodological Quality**: Very Good
**Quality of Evidence**: High
**Justification**: The alpha coefficient confirms strong item interrelatedness for a unidimensional construct.

**3. Reliability (mp3)**

**Property Rating**: ?
**Data**: Test-retest reliability was not assessed in this study.
**Methodological Quality**: Inadequate
**Quality of Evidence**: Very Low
**Justification**: Without test-retest data, reliability over time cannot be confirmed, resulting in very low evidence for this property.

**4. Measurement Error (mp4)**

**Property Rating**: ?
**Data**: Measurement error not calculated or reported.
**Methodological Quality**: Inadequate
**Quality of Evidence**: Very Low
**Justification**: Measurement error was not evaluated.

**5. Hypothesis Testing for Construct Validity (mp5)**

**Property Rating**: +
**Data**: The CWS showed strong convergent and divergent validity: Correlation with CAS: $r = 0.75$, $p < 0.001$ (convergent validity); Correlation with fatigue measure (CIS-Fatigue): $r = 0.34$, $p < 0.001$; Correlation with empowerment (CEQ): $r = -0.22$, $p < 0.001$ (divergent validity).
**Methodological Quality**: Very Good
**Quality of Evidence**: High
**Justification**: Correlations confirm construct validity, and hypothesis clearly stated at priori and provided support of expected associations.

**6. Cross-Cultural Validity (mp6)**

**Property Rating**: ?
**Data**: The study evaluated a Dutch translation of the Cancer Worry Scale (CWS), but no statistical tests of measurement equivalence (e.g., differential item functioning or measurement invariance) were performed to establish cross-cultural validity relative to the original English version.
**Methodological Quality**: Inadequate
**Quality of Evidence**: Very Low
**Justification**: Despite appropriate translation and psychometric testing in a Dutch sample, COSMIN requires formal statistical testing to evaluate whether the instrument performs equivalently across cultural groups. In the absence of such testing, this property remains indeterminate.

**7. Criterion Validity (mp7)**

**Property Rating**: +
**Data**: The ROC analysis demonstrated good diagnostic ability for distinguishing high and low FCR levels: AUC: 0.88 ($p < 0.001$, 95% CI = 0.83–0.93); Sensitivity: 96%, Specificity: 56% at a cutoff score of 11; sensitivity of 77% and specificity of 81% at a cutoff of 13
**Methodological Quality**: Very Good
**Quality of Evidence**: High

**Justification**: Strong AUC and specificity/sensitivity scores from ROC analysis support the CWS-8 criterion validity.

**8. Responsiveness (mp8)**

**Property Rating**: ?
**Data**: The study did not evaluate responsiveness.
**Methodological Quality**: Inadequate
**Quality of Evidence**: Very Low
**Justification**: Responsiveness was outside the study's scope, focusing instead on establishing cut-off points.

**Overall Summary and Quality Determination**

The CWS was originally developed by Lerman and colleagues in the early 1990s as a 6-item tool to assess cancer-related worry in individuals at increased risk of hereditary cancer [63]. This version evaluated both the frequency and functional impact of cancer-related thoughts and was widely used in genetic counseling contexts. The Dutch team [64] translated the scale and added two items related to worry about family members and future surgery, creating an 8-item Dutch version that was initially used in hereditary cancer research contexts.

Custers et al. [40] in 2014 were the first to validate the 8-item CWS version in a sample of Dutch breast cancer survivors, confirming a unidimensional structure ($\alpha$ = .87) and establishing initial psychometric support and clinical utility through ROC-based cut-off thresholds. The ROC curve analysis conducted against a 2-item version of the Cancer Acceptance Scale (CAS) to determine cut-off scores for clinical screening revealed two thresholds. A screening cut-off of ≥12 (sensitivity = 96%, specificity = 56%) and a diagnostic cut-off of ≥14 (sensitivity = 77%, specificity = 81%) were reported, supporting the scale's ability to differentiate survivors with high levels of FCR. Overall, the CWS showed sufficient structural validity, strong construct and criterion validity, and good reliability, supporting its clinical utility for FCR detection (see Table 4 for full rating assessment). The CWS-8 items scale by Custers et al. is therefore classified as Category A according to COSMIN guidelines.

**Category Determination Based on COSMIN Criteria**

**Category A**: The CWS-8 items by Custers et al. [40] meets the requirements for a Category A measurement instrument, demonstrating strong evidence for criterion validity, internal consistency, and structural validity. Additional studies are recommended to explore the scale's test-retest reliability, cross-cultural validity, and responsiveness.

**Conclusion**
The CWS by Custers et al. [40] is a psychometrically strong PROM for assessing FCR among Dutch breast cancer survivors. It is supported by sufficient structural validity and internal consistency and demonstrates strong construct validity. While test–retest reliability and responsiveness remain to be evaluated, the current evidence supports its use in both clinical and research contexts. The CWS-8 items scale is therefore classified as Category A according to COSMIN guidelines.

**Custers et al., 2018 / CWS-6 items [41]**

**Full Reference:** Custers, J. A. E., Kwakkenbos, L., van de Wal, M., Prins, J. B., & Thewes, B. (2018). Re-validation and screening capacity of the 6-item version of the Cancer Worry Scale. *Psycho-Oncology, 27*(11), 2609–2615. https://doi.org/10.1002/pon.4782

**Content Validity Assessment: Sufficient**
The content validity of the 6-item Cancer Worry Scale (CWS) is rated as sufficient based on a robust adaptation and validation process. Originally developed to assess concerns about cancer recurrence, the CWS underwent expert consultation and item revisions to enhance its applicability for a range of cancers, including breast, prostate, and colorectal cancers. The Dutch adaptation preserved the core content of the original Lerman et al. scale [63], with minor adjustments for cultural relevance, ensuring its relevance across survivor populations. Thus, content validity is rated as sufficient, supported by expert review and minor cultural adjustments. The CWS-6 item by Custers et al. [41] serves as a brief, effective tool for assessing FCR across different survivor groups.

Permission from original author(s): New PROM developed by first author
Original authors involved in study team: Yes

**Measurement Property Assessment**

**1. Structural Validity (mp1)**

**Property Rating:** +
**Data:** CFA indicated strong fit for a single-factor structure: Fit Indices: $\chi$29 = 122.4, P < 0.001, TLI = 0.99, CFI = 0.99, RMSEA = 0.11.
**Methodological Quality:** Very Good
**Quality of Evidence:** High
**Justification:** The CFA confirmed the one-factor model, supporting the unidimensional structure and alignment with the theoretical foundations of cancer worry.

**2. Internal Consistency (mp2)**

**Property Rating:** +
**Data:** The 6-item CWS demonstrated high internal consistency with a Cronbach's alpha of 0.90.
**Methodological Quality:** Very Good
**Quality of Evidence:** High
**Justification:** High Cronbach's alpha values confirm strong internal consistency, supporting reliable responses across items.

**3. Reliability (mp3)**

**Property Rating:** ?
**Data:** Test-retest reliability was not assessed in this study.
**Methodological Quality:** Inadequate
**Quality of Evidence:** Very Low
**Justification:** The absence of test-retest data limits evaluation of temporal stability, resulting in a very low

rating for this property.

**4. Measurement Error (mp4)**

**Property Rating:** Not assessed
**Justification:** Measurement error was not evaluated, with the study focusing on structural validity, internal consistency, and cut-off validation.

**5. Hypothesis Testing for Construct Validity (mp5)**

**Property Rating:** +
**Data:** Convergent and divergent validity were supported: Correlation with FCRI total score: r = 0.80; correlation with HADS-anxiety: r = 0.64; emotional functioning subscale (EORTC QLQ-C30): r = -0.59
**Correlation with FCRI total score:** r = 0.80
**Methodological Quality:** Very Good
**Quality of Evidence:** High
**Justification:** Significant correlations aligned with theoretical expectations, confirming construct validity.

**6. Cross-Cultural Validity (mp6)**

**Property Rating:** ?
**Data:** Cross-cultural validity was not directly evaluated; the CWS has primarily been validated in Dutch samples. Still, no formal DIF or subgroup analysis was reported.
**Methodological Quality:** Inadequate
**Quality of Evidence:** Very Low
**Justification:** Further research is needed to assess the scale's validity across diverse cultural groups.

**7. Criterion Validity (mp7)**

**Property Rating:** +
**Data:** ROC analysis demonstrated good diagnostic ability for high and severe FCR, using the FCRI-SF as a gold standard: AUC: 0.90 at FCRI-SF cut-off of 13; 0.93 at cut-off of 22; Sensitivity and Specificity: Sensitivity at cut-off 9 vs 10 = 82%, specificity = 83%; at cut-off 11 vs 12 = 88% sensitivity, 81% specificity
**Methodological Quality:** Very Good
**Quality of Evidence:** High
**Justification:** Strong AUC, sensitivity, and specificity values confirm the CWS-6 items by Custers et al. 2018 ability to detect clinically significant FCR.

**8. Responsiveness (mp8)**

**Property Rating:** ?
**Data:** Responsiveness was not evaluated.
**Methodological Quality:** Inadequate
**Quality of Evidence:** Very Low
**Justification:** No data were reported on the scale's ability to detect change over time.

**Overall Summary and Quality Determination**

Custers et al. [41] validated the 6-item version of the Cancer Worry Scale (CWS-6) in a large Dutch sample of 981 participants. The study confirmed a unidimensional structure through CFA, with strong internal consistency and high-quality evidence for both construct and criterion validity. Although test–retest reliability, responsiveness, and cross-cultural validity were not assessed, the methodological rigor and strong psychometric indicators across key properties support confidence in the instrument's clinical use.

**Category Determination Based on COSMIN Criteria**

**Category A –** The CWS-6 obtained sufficient evidence of content validity, at least low-quality evidence for sufficient structural validity, and high internal consistency, fulfilling COSMIN's threshold for recommended use. Further research is recommended to establish test-retest reliability and cross-cultural validity.

**Conclusion**
The CWS-6 by Custers et al. [41] is a psychometrically robust instrument for assessing FCR. Its brevity and diagnostic performance support its use as a reliable and valid screening tool, particularly in Dutch-speaking populations. Further research is warranted to confirm its responsiveness and measurement invariance across cultures.

**Chirico 2022 / CWS-8 items [42]**

**Reference:** Chirico, A., Vizza, D., Valente, M., Lo Iacono, M., Campagna, M. R., Palombi, T., Alivernini, F., Lucidi, F., & Bruno, F. (2022). Assessing the fear of recurrence using the Cancer Worry Scale in a sample of Italian breast cancer survivors. *Supportive Care in Cancer*, 30(4), 2829–2837. https://doi.org/10.1007/s00520-021-06718-4

**Content Validity Assessment: Sufficient**
Chirico et al. [42] set out to culturally adapt and linguistically validate, using back-translation, and expert review, an Italian version of the CWS-8 **[**40**]**. Content equivalence was confirmed through expert consultation and pretesting among Italian breast cancer survivors, with no changes to item wording or conceptual scope. The original 8-item structure was preserved while being culturally tailored for Italian breast cancer survivors, ensuring relevance and conceptual fidelity to the original construct of cancer worry. Thus, content validity is rated as sufficient based on rigorous cross-cultural adaptation and translation processes, supporting the scale's relevance for Italian breast cancer survivors.

Permission from original author(s) of the scale: Not reported
Original scale authors involved in study team: No

**Measurement Property Assessment**

**1. Structural Validity (mp1)**

**Property Rating:** +
**Data:** An exploratory factor analysis EFA indicated a two-factor structure for the CWS, labeled as "cancer worries" and "worries impact," with 63% of variance explained. Fit indices were: RMSEA: 0.07; TLI: 0.96; SRMR: 0.03
**Methodological Quality:** Adequate
**Quality of Evidence:** Moderate

**Justification:** The two-factor structure is robust and well-supported by EFA, confirming structural validity for the Italian sample. Lack of CFA limits the strength of evidence. No formal cross-loading analysis was reported.

**2. Internal Consistency (mp2)**

**Property Rating:** +
**Data:** Cronbach's $\alpha$ = 0.90 (total scale); $\alpha$ = 0.85 (Factor 1: Cancer Worries); $\alpha$ = 0.74 (Factor 2: Worries Impact)
**Methodological Quality:** Very Good
**Quality of Evidence:** High
**Justification:** All subscale values exceeded the acceptable threshold of 0.70, consistent with COSMIN guidelines, and based on a sufficient structural validity model.

**3. Reliability (mp3)**

**Property Rating:** ?
**Data:** No SEM, SDC, or MIC reported.
**Methodological Quality:** Inadequate
**Quality of Evidence:** Very Low
**Justification:** Measurement error could not be determined.

**4. Measurement Error (mp4)**

**Property Rating:** Not Assessed
**Data:** No specific measurement error data were provided.
**Methodological Quality:** Inadequate
**Quality of Evidence:** Very Low
**Justification:** Measurement error was not evaluated in this study.

**5. Hypothesis Testing for Construct Validity (mp5)**

**Property Rating:** +
**Data:** Positive correlations in expected directions for HADS-anxiety: r = 0.654; HADS-depression: r = 0.558; Fatigue Severity Scale (FSS): r = 0.449; CBI-self-efficacy: r = –0.589
**Methodological Quality:** Very Good
**Quality of Evidence:** High
**Justification:** All predefined hypotheses were confirmed with moderate to strong correlations in expected directions.

**6. Cross-Cultural Validity (mp6)**

**Property Rating: -**
**Data:** Although the authors state "the Italian version demonstrated comparable psychometric properties to the original CWS," no formal DIF analysis or item-level comparison was conducted. The only comparisons were between subgroups within the Italian sample, eg, high vs low fear groups and disease stage.
**Methodological Quality:** Inadequate

**Quality of Evidence:** Low
**Justification:** COSMIN defines cross-cultural validity as requiring formal testing of measurement invariance across populations. Although psychometric similarity is noted, the lack of statistical testing across cultures renders the rating indeterminate.

**7. Criterion Validity (mp7)**

**Property Rating:** +
**Data:** ROC analysis using CAS as the criterion showed AUC = 0.87 (95% CI: 0.80–0.94); Optimal cut-off = 15/16 with sensitivity = 74%, specificity = 85%
**Methodological Quality:** Very Good
**Quality of Evidence:** High
**Justification:** Strong AUC and classification metrics indicate that the CWS is effective for identifying clinically significant FCR.

**8. Responsiveness (mp8)**

**Property Rating:** Not Assessed
**Data:** No responsiveness analysis performed.
**Methodological Quality:** Inadequate
**Quality of Evidence:** Very Low
**Justification:** Responsiveness to change over time was not evaluated.

**Overall Summary and Quality Determination**

Chirico et al.[42] validated an Italian version of the 8-item CWS [40] in a sample of breast cancer survivors. The scale demonstrated strong structural validity, with a two-factor model ("cancer worries" and "worries impact") explaining 63% of variance and meeting COSMIN fit thresholds (TLI = 0.96, SRMR = 0.03, RMSEA = 0.07). Internal consistency was high for the total score ($\alpha$ = 0.90) and acceptable for subscales ($\alpha$ = 0.85 and 0.74). Construct validity was supported by strong correlations with HADS-anxiety (r = 0.654) and the FCRI (r = 0.80), while criterion validity was confirmed via ROC analysis against the Cancer Acceptance Scale (AUC = 0.871; sensitivity = 74%, specificity = 85%).

Test–retest reliability was mentioned but lacked reported ICC values. Measurement error and responsiveness were not assessed. Cross-cultural validity was not formally tested using DIF or invariance methods, despite claims of comparable performance. Nonetheless, the Italian CWS met COSMIN Category A criteria due to sufficient evidence across core psychometric properties. Further cross-cultural testing and longitudinal validation would strengthen its broader applicability.

**COSMIN Category Determination**

**Category A:** The Italian CWS-8 meets the COSMIN requirements for Category A. It demonstrates sufficient content validity (through translation and adaptation), sufficient internal consistency (supported by structural validity), and adequate performance on hypothesis testing and criterion validity. While certain properties such as measurement error and responsiveness are not assessed, and cross-cultural validity is lacking, these are not barriers to inclusion in Category A given the core requirements are met.

**Conclusion**

The Italian version of the CWS-8 by Chirico et al.[42] demonstrates sound psychometric properties and is suitable for use in assessing fear of cancer recurrence among Italian-speaking breast cancer survivors for both clinical and research settings in this population. Future studies could further explore its measurement error and responsiveness to solidify its utility.

**3.2 FCR4 and FCR7 (Humphris et al., 2018) [43]: Validation and Adaptation of the New PROM in New Languages and Populations**
**Humphris et al., 2018 / FCR4/FCR7 [43]**

**Reference:** Humphris, G. M., Watson, E., Sharpe, M., & Ozakinci, G. (2018). Unidimensional scales for fears of cancer recurrence and their psychometric properties: The FCR4 and FCR7. *Health and Quality of Life Outcomes, 16*(30), 1–12. https://doi.org/10.1186/s12955-018-0850-x

**Content Validity Assessment: Sufficient**
The FCR4 and FCR7 items were drawn from existing validated measures of fear of cancer recurrence (FCRI, CARS, Worry of Cancer Scale, FORPSYCH study), reviewed by experts in the study team, and piloted with patients at the Edinburgh Cancer Centre to assess reactions and comprehension. The authors report selecting the final item pool through a "consensus approach of cycling through these items between the authors and also piloting them with patients at the ECC." The Flesch readability index scores were 60.2 for the FCR7 and 55.1 for the FCR4, indicating adequate readability. The sample population included 206 breast cancer survivors and 53 colorectal cancer survivors.

Permission from original author(s) of the scale: New PROM developed by first author
Original scale authors involved in study team: Yes

**Measurement Property Assessment**

**1. Structural Validity (mp1)**

**Property Rating:** +
**Data:** EFA results supported a unidimensional structure for both FCR4 and FCR7: KMO: 0.86 for FCR4 and 0.92 for FCR7, exceeding the threshold of 0.80; Eigenvalue: The single retained factor had an eigenvalue of 3.3 for FCR4 and 4.8 for FCR7, both > 1; The model explained over 50% of the variance (precise values not provided).On factor loadings, strong loadings were reported, all above >0.70 for most items, with only one item in FCR7 loaded at 0.52, but item retained due to its theoretical relevance. ). No CFA conducted. No cross-loadings reported.
**Methodological Quality:** Adequate
**Quality of Evidence:** Moderate
**Justification:** EFA supported a clear one-factor solution for both versions. However, no CFA or formal cross-loading analysis was performed, limiting the strength of evidence.

**2. Internal Consistency (mp2)**

**Property Rating:** +
**Data:** Cronbach's alpha values for FCR4: $\alpha = 0.93$; FCR7: $\alpha = 0.92$. Both exceed the threshold of 0.7, indicating excellent internal consistency.
**Methodological Quality:** Very Good
**Quality of Evidence:** High

**Justification:** High Cronbach's alpha values support strong internal consistency, with no downgrades required.

**3. Reliability (mp3)**

**Property Rating:** ?
**Data:** Test-retest reliability was not assessed.
**Methodological Quality:** Inadequate
**Quality of Evidence:** Very Low
**Justification:** Lack of test-retest data leads to a very low rating for this property.

**4. Measurement Error (mp4)**

**Property Rating:** ?
**Data:** Measurement error was not reported.
**Methodological Quality:** Inadequate
**Quality of Evidence:** Very Low
**Justification:** No measurement error data, resulting in a very low rating.

**5. Hypothesis Testing for Construct Validity (mp5)**

**Property Rating:** +
**Data:** Significant correlations with the Hospital Anxiety and Depression Scale (HADS): FCR4: Correlations with HADS Anxiety ($r = 0.65$, $p < 0.01$) and HADS Depression ($r = 0.36$, $p < 0.01$). FCR7: Correlations with HADS Anxiety ($r = 0.68$, $p < 0.01$) and HADS Depression ($r = 0.40$, $p < 0.01$). Strong correlations with perceived risk of recurrence and reminders from minor aches/pains, supporting convergent validity.
**Methodological Quality:** Very Good
**Quality of Evidence:** High
**Justification:** A priori hypotheses tested and supported; appropriate comparators used.

**6. Cross-Cultural Validity (mp6)**

**Property Rating:** ?
**Data:** Although the FCR4 and FCR7 were applied to two cancer groups (breast and colorectal) in the UK, both groups were recruited from a single English-speaking population (Scotland). No cross-national or cross-linguistic comparison was conducted, nor were any measurement invariance tests (e.g., MG-CFA or DIF) reported to assess whether the tool performs equivalently across diverse cultural or linguistic groups.
**Methodological Quality:** Inadequate
**Quality of Evidence:** Very Low
**Justification:** Despite good internal consistency and factorial validity within a UK sample, COSMIN standards require statistical testing for measurement equivalence across cultures to assess cross-cultural validity. Since the study did not assess item functioning across language or cultural groups, construct validity (mp5) cannot be rated as sufficient.

**7. Criterion Validity (mp7)**

**Property Rating:** +
**Data:** Discriminant ability was evaluated using ROC curve analysis against the HADS-Anxiety subscale as an external criterion. For FCR4, Cohen's d = 1.87 indicated a strong effect size in differentiating high vs. low anxiety cases.
**Methodological Quality:** Very Good
**Quality of Evidence:** High
**Justification:** COSMIN allows conceptually related tools when no gold standard exists; evidence supports a sufficient rating. Criterion validity is supported, and the ROC analysis strengthen this evidence.

**8. Responsiveness (mp8)**

**Property Rating:** ?
**Data:** Responsiveness was not assessed.
**Methodological Quality:** Inadequate
**Quality of Evidence:** Very Low
**Justification:** Lack of data results in a very low rating.

**Overall Summary and Quality Determination**

The FCR4 and FCR7, developed by Humphris et al.[43], were validated together in a sample of English-speaking breast (n = 206) and colorectal (n = 53) cancer patients. The FCR4 comprises the first four items of the FCR7, while the latter includes three additional items. Both PROMs are unidimensional and were designed to offer brief, psychometrically sound assessments of FCR. Exploratory factor analysis confirmed a single-factor structure, and internal consistency was excellent for both versions ($\alpha$ = 0.93 for FCR4; $\alpha$ = 0.92 for FCR7).

Construct validity was supported through significant correlations with anxiety and depression (HADS), and criterion validity was confirmed via ROC analyses using HADS-Anxiety as a comparator. Test–retest reliability, cross-cultural validity, measurement error, and responsiveness were not assessed. Based on the available evidence, both tools meet COSMIN Category A criteria. The authors noted that either version may be suitable depending on the intended use—screening or longitudinal monitoring.

**COSMIN Category Determination**

**Category A:** The FCR4 and FCR7 meet COSMIN's criteria for Category A: sufficient content validity, at least low-quality evidence for sufficient structural validity, and high internal consistency. While further research on cross-cultural validity, test–retest reliability, and responsiveness is warranted, both versions are recommended for clinical and research use in English-speaking adult cancer populations.

**Conclusion**
The FCR4 and FCR7 scales by Humphris et al. [43] are recommended tools for assessing FCR, with strong psychometric support for content validity, internal consistency, and structural validity. Designed to be easy to administer and low burden, these scales are well-suited for use in clinical and research contexts. Further studies to assess test-retest reliability and cross-cultural applicability would enhance their generalizability across diverse patient populations.

**Yang et al., 2019 / Chinese version of the FCR723 FCR7 [44]**

**Reference:** Yang, Y., Humphris, G., Sun, H., Li, W., Hao, Y., Liu, T., Zhang, J., Wang, H., & Zhang, B. (2019). Psychometric properties of the Chinese version Fear of Cancer Recurrence Questionnaire-7 (FCR-7). *Professional Psychology: Research and Practice.* Advance online publication. https://doi.org/10.1037/pro0000257

**Content Validity Assessment:** Sufficient
Yang et al. [44] adapted the Chinese FCR-7 from the original English version [43], applying Bracken and Barona's [60] translation-back translation method. A panel of experts, including oncologists, psychologists, and a nursing specialist, assessed content equivalence, yielding an overall Content Validity Index (CVI) of 88% (94% excluding Item 6). Thus, content validity is considered sufficient, based on rigorous translation procedures and expert panel evaluation using quantitative CVI metrics. Total sample included 1025 mixed cancer patients.

Permission from original author(s) of the scale: Yes
Original scale authors involved in study team: Yes

**Measurement Property Assessment**

**1. Structural Validity (mp1)**

**Property Rating:** +
**Data:** Both EFA and CFA were conducted. EFA indicated a single-factor structure, explaining 65.37% of the variance with eigenvalue at 4.26, supporting unidimensionality. CFA results confirmed this structure with the following fit indices: CFI: 0.996; RMSEA: 0.039 (95% CI [0.01, 0.07]); and $\chi^2$/df: 1.79 (below the recommended threshold of 3.0)
**Methodological Quality:** Very Good
**Quality of Evidence:** High
**Conclusion:** Structural validity is considered sufficient, with strong evidence supporting the factor structure.
**2. Internal Consistency (mp2)**

**Property Rating:** +
**Data:** The Cronbach's alpha for the Chinese FCR-7 was 0.87, slightly lower than the original English version ($\alpha$ = 0.92), but well above the threshold of 0.70; item-total correlations mostly strong.
**Methodological Quality:** Very Good
**Quality of Evidence:** High
**Conclusion:** Internal consistency is sufficient and well-supported.

**3. Reliability (mp3)**

**Property Rating:** +
**Data:** Test-retest reliability was assessed after one month with 285 participants, yielding a Pearson correlation coefficient of r = 0.90, indicating excellent reliability.
**Methodological Quality:** Very Good
**Quality of Evidence:** High
**Conclusion:** Reliability is supported with high-quality evidence.

**4. Measurement Error (mp4)**

**Property Rating:** ?
**Data:** Measurement error was not reported.
**Methodological Quality:** Inadequate
**Quality of Evidence:** Very Low
**Conclusion:** Measurement error remains indeterminate.

**5. Hypothesis Testing for Construct Validity (mp5)**

**Property Rating:** +
**Data:** Convergent validity was assessed via correlations with related scales: FoP-Q-SF: $r = 0.756$; PHQ-9 (depression): $r = 0.522$; and GAD-7 (anxiety): $r = 0.553$. All correlations were strong and statistically significant ($p < 0.01$), aligning with related constructs.
**Methodological Quality:** Very Good
**Quality of Evidence:** High
**Conclusion:** Construct validity is well-supported by high-quality evidence.

**6. Cross-Cultural Validity (mp6)**

**Property Rating:** ?
**Data:** While the Chinese version of the FCR-7 was translated and culturally adapted following established guidelines, its cross-cultural validity was not directly evaluated; no formal DIF or subgroup analysis was reported to evaluate cross-cultural measurement equivalence (e.g., DIF or measurement invariance). The authors did not compare the Chinese version with other language versions or test for equivalence across groups.
**Methodological Quality:** Inadequate
**Quality of Evidence:** Very Low
**Conclusion:** While the translation process and content review by experts were well-documented, COSMIN requires statistical testing (e.g., DIF or MG-CFA) to support cross-cultural validity. The absence of such analysis means the property cannot be rated as sufficient, and the evidence is very low in quality.

**7. Criterion Validity (mp7)**

**Property Rating:** +
**Data:** Criterion validity was indirectly supported by strong correlations with FoP-Q-SF, PHQ-9, and GAD-7, though no ROC analysis was conducted.
**Methodological Quality:** Adequate
**Quality of Evidence:** Moderate
**Conclusion:** Criterion validity is supported, though full assessment through ROC analysis could strengthen this evidence.

**8. Responsiveness (mp8)**

**Property Rating:** ?
**Data:** Responsiveness was not assessed.
**Methodological Quality:** Inadequate

**Quality of Evidence:** Very Low
**Conclusion:** Responsiveness remains indeterminate.

**Overall Summary and Quality Determination**

Yang et al. [44] conducted a comprehensive validation of the Chinese version in a large oncology sample (N = 1,025). Content validity was rated sufficient based on a rigorous forward–backward translation and expert panel evaluation (CVI = 88%). Structural validity was supported by both EFA (unidimensional solution, 65.37% variance explained) and CFA (CFI = 0.996; RMSEA = 0.039; $\chi^2$/df = 1.79), with high methodological quality. Internal consistency ($\alpha$ = 0.87) and test–retest reliability over one month (r = 0.90) were rated sufficient with high-quality evidence. Hypothesis testing for construct validity was confirmed through strong correlations with the FoP-Q-SF (r = 0.756), PHQ-9 (r = 0.522), and GAD-7 (r = 0.553). Criterion validity was indirectly supported by these same correlations. Measurement error and responsiveness were not assessed. Cross-cultural validity was rated indeterminate due to the absence of formal DIF or subgroup analysis. Overall, the Chinese FCR7 met COSMIN Category A criteria and is recommended for use in Chinese-speaking populations.

The study by Yang et al. [44] validated the Chinese version of the FCR-7 in a sample of 1,025 cancer patients, with strong evidence for structural validity (via EFA), internal consistency, test–retest reliability, and construct and criterion validity. The psychometric performance of the scale was robust across metrics relevant to reliability and validity. However, measurement error, responsiveness, and cross-cultural validity were not evaluated.

**COSMIN Category Determination**

**Category A:** The Chinese FCR-7 meets COSMIN's criteria for Category A, as it demonstrates sufficient content validity, at least low-quality evidence for sufficient structural validity, and high internal consistency. It is recommended for use in both clinical and research settings involving Chinese-speaking cancer populations.

**Conclusion**
The Chinese version of the FCR-7 by Yang et al. [44] falls into Category A according to COSMIN guidelines. This scale demonstrates robust psychometric properties, making it suitable for assessing fear of cancer recurrence among Chinese-speaking cancer populations. Further studies are recommended to evaluate measurement error and responsiveness for broader applicability.

**Lee et al. 2020 / FCR7-Chinese [45]**

**Reference:** Lee, Y.-H., Hu, C.-C., Humphris, G., Huang, I.-C., You, K.-L., Jhang, S.-Y., Chen, J.-S., et al. (2020). Screening for fear of cancer recurrence: Instrument validation and current status in early stage lung cancer patients. *Journal of the Formosan Medical Association = Taiwan Yi Zhi*, *119*(6), 1101–1108.

**Content Validity Assessment: Sufficient**
The FCR7 [43] was translated into Chinese using forward–backward translation principles. Content validity of the FCR7-C was assessed by five experts (two oncologists, two psycho-oncology nurse researchers, and one oncology nurse), who judged the translated items to be satisfactory. Face validity was assessed through interviews with 10 early-stage lung cancer patients recruited from a thoracic surgery outpatient clinic in northern Taiwan. All patients confirmed that the FCR7-C was relevant and

easy to understand. Total sample in the study included 160 lung cancer survivors.

Permission from original author(s) of the scale: New PROM developed by first author
Original scale authors involved in study team: Yes

**Measurement Property Assessment**

**1. Structural Validity (mp1)**

**Property Rating**: +
**Data**: Confirmatory factor analysis was conducted using AMOS. Model fit indices were: CFI = 0.97, SRMR = 0.04, RMSEA = 0.09, $\chi^2 = 85.98$, $p < 0.001$, $\chi^2/df = 2.4$. Factor loadings ranged from 0.42 (item 6) to 0.97.
**Methodological Quality**: Very Good
**Quality of Evidence**: High
**Justification**: CFA met key COSMIN thresholds (CFI = 0.97, SRMR = 0.04, $\chi^2/df = 2.4$); RMSEA was slightly above ideal. Sample size of n = 160 is fully sufficient for 7-item unidimensional CFA, per COSMIN criteria.

**2. Internal Consistency (mp2)**

**Property Rating**: +
**Data**: Cronbach's $\alpha = 0.90$ for the 7-item FCR7-C. Item-total correlations ranged from 0.72 to 0.85 for six items, and 0.45 for item 6.
**Methodological Quality**: Very Good
**Quality of Evidence**: High
**Justification**: Internal consistency exceeds the COSMIN threshold of 0.70 and is interpretable due to sufficient structural validity. All item-total correlations met or exceeded generally accepted minimums (≥ 0.30), and the sample size was adequate. No concerns were identified to warrant downgrading.

**3. Reliability (mp3)**

**Property Rating**: ?
**Data**: No test–retest reliability (e.g., ICC, time interval, or retest sample) was reported
**Methodological Quality**: Inadequate
**Quality of Evidence**: Very Low
**Justification**: No information was provided on test–retest design or results. Property cannot be rated.

**4. Measurement Error (mp4)**

**Property Rating**: ?
**Data**: No data were provided on standard error of measurement (SEM), smallest detectable change (SDC), or limits of agreement (LoA).
**Methodological Quality**: Inadequate
**Quality of Evidence**: Very Low
**Justification**: Absence of measurement error data results in a very low evidence rating.

**5. Hypothesis Testing for Construct Validity (mp5)**

**Property Rating**: +

**Data**: FCR7-C was positively correlated with anxiety (r = 0.62) and depression (r = 0.38); negatively correlated with age (r = –0.33), physical function (r = –0.31), and QOL (r = –0.30).
**Methodological Quality**: Very Good
**Quality of Evidence**: High
**Justification**: Five theoretically expected correlations were reported and supported, meeting COSMIN criteria for sufficient hypothesis testing. Sample size is adequate.

**6. Cross-Cultural Validity (mp6)**
**Property Rating**: ?
**Data**: No analysis of differential item functioning (DIF) or multi-group confirmatory factor analysis (MG-CFA) was reported.
**Methodological Quality**: Inadequate
**Quality of Evidence**: Very Low
**Justification**: Measurement error remains indeterminate, no DIF or MG-CFA were reported.

**7. Criterion Validity (mp7)**
**Property Rating**: +
**Data**: FCR7-C was positively correlated with anxiety (r = 0.62) and depression (r = 0.38).
**Methodological Quality**: Adequate
**Quality of Evidence**: Moderate
**Justification**: COSMIN allows conceptually related tools when no gold standard exists; evidence supports a sufficient rating. Criterion validity is supported, though full assessment through ROC analysis could strengthen this evidence.

**8. Responsiveness (mp8)**

**Property Rating**: ?
**Data**: Responsiveness was not assessed
**Methodological Quality**: Inadequate
**Quality of Evidence**: Very Low
**Justification**: No data on change scores, responsiveness, or intervention impact was reported.

**Overall Summary and Quality Determination**
Lee et al. [45] validated the Chinese FCR7 (FCR7-C) in a sample of 160 early-stage lung cancer patients. The instrument demonstrated sufficient content validity through expert and patient involvement. Structural validity was confirmed via CFA (CFI = 0.97, SRMR = 0.04), and internal consistency was high ($\alpha$ = 0.90). Construct validity and criterion validity were both supported by meaningful correlations with psychological and functional variables. However, test–retest reliability, measurement error, cross-cultural invariance, and responsiveness were not evaluated.

**COSMIN Category Determination**

**Category A :** The FCR7-C PROM meets COSMIN criteria for sufficient content validity, structural validity (mp1), and internal consistency (mp2). Additional measurement properties (e.g., reliability, responsiveness) were not reported, but no serious flaws were identified. Therefore, the FCR7-C qualifies for Category A — recommended for use.

**Conclusion**

The FCR7-C [44] is a psychometrically sound and recommended tool for assessing fear of cancer recurrence in Chinese-speaking patients with early-stage lung cancer. With strong structural integrity, internal consistency, and supported validity, it meets COSMIN's Category A threshold. Future research should address test–retest reliability, cross-cultural equivalence, and responsiveness.

**Braun et al. 2022 / FCR6-Brain [46]**

**Reference:** Braun, S. E., Willis, K. D., Mladen, S. N., Aslanzadeh, F., Lanoye, A., Langbein, J., Reid, M., et al. (2022). Introducing FCR6-Brain: Measuring fear of cancer recurrence in brain tumor patients and their caregivers. *Neuro-oncology practice*, *9*(6), 509–519.

**Content Validity Assessment: Sufficient**
The FCR6-Brain scale was adapted from the original FCR7 to better address the unique concerns of brain tumor patients and their caregivers. This study engaged in rigorous factor analysis to modify the original scale, removing an item related to physical self-checking that was less relevant for this population. This adjustment enhanced content alignment with the target group, addressing specific worries of patients with primary brain tumors and their caregivers. Thus, content validity is assumed to be sufficient, based on the modification of the FCR-7 and psychometric analysis in the target population. The study sample included 165 brain tumor cancer patients.

Permission from original author(s) of the scale: Not reported
Original scale authors involved in study team: No

**Measurement Property Assessment**

**1. Structural Validity (mp1)**

**Property Rating**: +
**Data**: Exploratory factor analysis with six items (patient version) yielded a single factor with an eigenvalue of 4.8, explaining 80.5% of the variance. All items loaded strongly onto the factor (> .80), and item intercorrelations ranged from 0.70 to 0.90. Although visual inspection of the scree plot suggested a potential two-factor model, only one factor exceeded the eigenvalue >1 criterion.
**Methodological Quality**: Adequate
**Quality of Evidence**: Moderate
**Justification**: The strong factor loadings, high total variance explained, and excellent intercorrelations meet the COSMIN criteria for a sufficient (+) rating using EFA. The one-factor solution is well-supported despite exploratory suggestions of two factors.

**2. Internal Consistency (mp2)**

**Property Rating**: +
**Data**: Cronbach's $\alpha = 0.91$ for the 6-item patient scale.
**Methodological Quality**: Very Good
**Quality of Evidence**: High
**Justification**: Internal consistency exceeds the COSMIN threshold of 0.70 and is supported by adequate structural validity data. The instrument demonstrates strong internal reliability.

**3. Reliability (mp3)**

**Property Rating**: ?
**Data**: Test-retest reliability was not assessed.
**Methodological Quality**: Inadequate
**Quality of Evidence**: Very Low
**Justification**: Lack of test-retest reliability data results in an indeterminate rating for this property.

**4. Measurement Error (mp4)**

**Property Rating**: ?
**Data**: No data reported on SEM, SDC, or MIC was reported.
**Methodological Quality**: Inadequate
**Quality of Evidence**: Very Low
**Justification**: Absence of measurement error data results in a very low evidence rating.

**5. Hypothesis Testing for Construct Validity (mp5)**

**Property Rating**: +
**Data**: Significant correlations were observed with related psychological measures with predefined hypotheses with Generalized Anxiety Disorder (GAD-7): $r = 0.7$, $p < 0.001$ (patients);
Patient Health Questionnaire (PHQ-9): $r = 0.5$, $p < 0.001$ (patients); Death Distress Scale (DDS): $r = 0.7$, $p < 0.001$ (patients and caregivers)
**Methodological Quality**: Very Good
**Quality of Evidence**: High
**Justification**: The consistent correlations provide high-quality evidence for construct validity. Correlations were consistent with theoretical expectations and hypotheses were specified a priori.

**6. Cross-Cultural Validity (mp6)**

**Property Rating**: ?
**Data**: Although the study includes both in-person and online recruitment across different U.S. regions, all participants had to be literate in English, and there is no mention of translation or formal testing of measurement invariance (e.g., DIF or MG-CFA), despite claims that the FCR6-Brain may generalize to a broader population.
**Methodological Quality**: Inadequate
**Quality of Evidence**: Very Low
**Justification**: Although the study used both in-person and online recruitment to improve sample diversity, all participants completed the English version of the scale. No statistical tests of item-level equivalence across cultural or language groups were conducted, which precludes a valid assessment of cross-cultural validity according to COSMIN guidelines.

**7. Criterion Validity (mp7)**

**Property Rating**: +
**Data**: Strong correlations were observed between the FCR-6 Brain and validated measures of anxiety, depression, anxiety, and trauma-related intrusion. Sensitivity and specificity thresholds were noted above the 60th percentile.
**Methodological Quality**: Adequate
**Quality of Evidence**: Moderate
**Justification**: Although no gold-standard structured interview for FCR was used, COSMIN allows for assessment of criterion validity via strong correlations with closely related constructs when no definitive gold standard exists.GAD-7 and DDS were conceptually aligned, and the observed correlations exceeded r = 0.50, with statistical significance and adequate sample size, supporting the validity of the PROM against appropriate proxies. Criterion validity is supported, though full assessment through ROC analysis could strengthen this evidence.

**8. Responsiveness (mp8)**

**Property Rating**: ?
**Data**: Responsiveness was not assessed.
**Methodological Quality**: Inadequate
**Quality of Evidence**: Very Low
**Justification**: Absence of responsiveness data leads to a very low evidence rating.

**Overall Summary and Quality Determination**

Braun et al. [46] developed the FCR6-Brain by adapting the original FCR7 [43] for use in individuals with primary brain tumors. Only data from cancer patients were included in this review. The PROM demonstrated robust structural validity, with a unidimensional factor structure explaining 80.5% of the variance and all items loading above 0.80. Internal consistency was excellent ($\alpha$ = 0.91). Content validity was supported through systematic item development and pilot testing. Construct and criterion validity were confirmed via strong, theoretically aligned correlations with the GAD-7 (r = 0.70), PHQ-9 (r = 0.50), and Death Distress Scale (r = 0.70). Although ROC analyses were not conducted, these associations supported clinical interpretability. The authors proposed cut-offs of ≥18 (clinically subthreshold) and ≥31 (clinically significant), based on percentile thresholds, highlighting elevated FCR burden in neuro-oncology populations.

Despite the absence of test–retest reliability, cross-cultural validation, measurement error, and responsiveness data, the FCR6-Brain meets COSMIN Category A criteria based on sufficient content, structural, and construct validity, and high internal consistency. It is recommended for use in clinical screening of FCR among individuals with brain tumors.

**COSMIN Category Determination**

**Category A**: The FCR6-Brain obtained sufficient evidence of content validity, at least low-quality evidence for sufficient structural validity, and high internal consistency, fulfilling COSMIN's threshold for recommended use. While additional testing (e.g., cross-cultural validation, responsiveness) is encouraged, this PROM can be recommended for use in clinical and research settings involving patients with brain tumors.

**Conclusion**

The FCR6-Brain by Braun et al. [46] is a psychometrically robust instrument for assessing FCR. Its brevity and diagnostic performance support its use as a reliable and valid screening tool, particularly in English-speaking brain tumor patients. Further research is warranted to confirm its responsiveness and measurement invariance across cultures.

**Iglesias-Puzas et al. 2022 / FCR7-Spanish [47]**

**Reference**: Iglesias-Puzas Á, García-González V, Conde-Taboada A, López-Bran E. Fear of cancer recurrence in patients with non-metastatic melanoma: Spanish validation and disease-related factors. Australas J Dermatol. 2022 Nov;63(4):e312-e319. doi: 10.1111/ajd.13907. Epub 2022 Jul 29. PMID: 35904493.

**Content Validity Assessment: Sufficient**
The authors followed Beaton et al.'s [58] guidelines for cross-cultural adaptation [58]. Dual forward translation, expert review, back translation, and cognitive debriefing in 15 volunteers confirmed item clarity and relevance. While the credentials of the pilot testers were not detailed, the translation process supports sufficient content validity. The study sample included 123 melanoma cancer survivors.

Permission from original author(s) of the scale: Not reported
Original scale authors involved in study team: No

**Measurement Property Assessment**

**1. Structural Validity (mp1)**

**Property Rating**: ?
**Data**: The authors conducted preliminary factor analysis and reported a Kaiser-Meyer-Olkin (KMO) value of 0.868 and Bartlett's test of sphericity as significant ($p < 0.05$). They also reported that only one item had an eigenvalue >1, which they interpreted as supporting unidimensionality. However, no further information was provided on factor loadings, percentage of variance explained, or cross-loadings.
**Methodological Quality**: Doubtful
**Quality of Evidence**: Low
**Justification**: While the study reported acceptable KMO and Bartlett's test values, it did not report essential components required by COSMIN to fully evaluate structural validity. These include the proportion of variance explained by the retained factor(s), factor loadings for each item, and potential cross-loadings. As such, it is unclear whether the FCR-7 demonstrated a sufficiently strong internal structure in this Spanish adaptation. Per COSMIN guidelines, the absence of these key statistics results in an indeterminate rating for structural validity and lowers the methodological quality.

**2. Internal Consistency (mp2)**

**Property Rating**: ?
**Data**: Cronbach's alpha value reported for the Spanish FCR7 was 0.834, above COSMIN of ≥ 0.70.
**Methodological Quality**: Very Good
**Quality of Evidence**: High
**Justification**: COSMIN requires at least low-quality evidence for sufficient structural validity to interpret internal consistency. Due to insufficient structural validity, internal consistency could not be determined and was rated as indeterminate.

**3. Reliability (Test-Retest) (mp3)**

**Property Rating:** +
**Data:** ICCs per item ranged from 0.75 to 0.88 (Table 2), sample = 25, test interval = 14–21 days.
**Methodological Quality:** Adequate
**Quality of Evidence:** Moderate
**Justification:** While ICC values were strong, the use of item-level ICCs instead of a total score ICC and a small sample (n = 25) limits methodological quality.

**4. Measurement Error (mp4)**

**Property Rating:** ?
**Data:** Not reported
**Methodological Quality:** Inadequate
**Quality of Evidence:** Very Low
**Justification:** No SDC, SEM, or MIC data were provided.

**5. Hypothesis Testing for Construct Validity (mp5)**

**Property Rating:** +
**Data:** FCR7 scores correlated with SCI-12 subscales (emotion and appearance), as expected.
**Methodological Quality:** Adequate
**Quality of Evidence:** Low
**Justification:** Only one convergent relationship was examined with a related QoL measure (SCI-12); sample size adequate, but COSMIN recommends ≥3 predefined hypotheses for stronger support.

**6. Cross-Cultural Validity (mp6)**

**Property Rating:** ?
**Data:** No formal DIF or MG-CFA performed.
**Methodological Quality:** Inadequate
**Quality of Evidence:** Very Low
**Justification:** While a translation process was conducted, no statistical testing for measurement invariance across groups was performed.

**7. Criterion Validity (mp7)**

**Property Rating:** ?
**Data:** Criterion validity was assessed against the Skin Cancer Index-12 (SCI-12), a quality of life (QoL) measure. However, QoL is conceptually distinct from fear of cancer recurrence (FCR). Furthermore, no data were provided on ROC, AUC, sensitivity, specificity, or correlations with a gold standard or a clearly related construct.
**Methodological Quality:** Inadequate
**Quality of Evidence:** Very Low
**Justification:** According to COSMIN guidelines, criterion validity should be assessed using a clear gold standard, or at minimum, a highly related construct that demonstrates strong correlations (typically >0.70). In the absence of a true gold standard for FCR, a proxy may be used, but only if it is conceptually close. The SCI-12, a general QoL instrument, does not closely reflect the specific construct of FCR, thereby limiting its validity as a comparator. Additionally, no quantitative evidence of diagnostic accuracy (e.g.,

ROC curve, AUC, sensitivity/specificity) or correlation values with the comparator was reported, preventing proper evaluation. Thus, methodological quality is rated as inadequate, and the quality of evidence is very low.

**8. Responsiveness (mp8)**

**Property Rating:** ?
**Data:** Not assessed
**Methodological Quality:** Inadequate
**Quality of Evidence:** Very Low
**Justification:** Responsiveness was not evaluated in the study.

**Overall Summary and Quality Determination**

Iglesias-Puzas et al. [47] conducted a validation of the FCR7 in Spanish for melanoma patients. Content validity was assessed as sufficient, internal consistency was acceptable, but structural validity was not sufficiently supported due to lack of confirmatory factor analysis. Criterion validity was evaluated against a quality of life measure (SCI-12), which is not conceptually close to FCR and no ROC, sensitivity/specificity, or correlation with a gold standard was provided. Construct validity, test–retest reliability, responsiveness, and measurement error were not assessed. Given the limited scope and low methodological quality in key domains, the validation evidence is very low, and the FCR7 in this context cannot be recommended for clinical or research use without further validation.

**Category Determination Based on COSMIN Criteria:**

**Category B**: Although the Spanish version of the FCR7 demonstrated sufficient reliability, hypothesis testing, and responsiveness, structural validity was rated as insufficient, and internal consistency was rated as indeterminate. According to COSMIN criteria, internal consistency can only be interpreted when structural validity is at least low-quality and sufficient. Therefore, the PROM does not meet the requirements for Category A. No high-quality evidence of serious flaws was identified that would warrant a Category C classification, thus this PROMs is classified as B.

**Conclusion:** The FCR7-Spanish shows promise for use in Spanish-speaking non-metastatic melanoma populations and demonstrates adequate performance across several measurement properties. However, due to the insufficient structural validity and resulting indeterminate rating for internal consistency, further psychometric validation is needed before this instrument can be recommended for routine clinical or research use.

**Nandakumar et al., 2022 [48]**

**Reference**: Nandakumar, R., Krishnan, V., Vidhubala, E., Surendran, P., & Sagar, R. (2022). Fear of cancer recurrence in breast cancer survivors: Validation of the FCR7-Tamil version. *Indian Journal of Cancer*, 59(1), 321–325.

**Content Validity Assessment: Sufficient**

The FCR7 by Nandakumar et al. [48] was translated into Tamil following international guidelines using a dual forward and backward translation protocol. Two bilingual experts conducted independent forward translations, followed by reconciliation. Two additional experts back-translated the reconciled version

into English to identify ambiguities. A panel of subject matter experts reviewed the final version for content accuracy, finding alignment between item content and intended FCR constructs (e.g., anxiety, cognition, behavioral response). Face validity was assessed with 10 breast cancer survivors who confirmed item clarity, contextual relevance, and natural language. Feedback confirmed semantic and conceptual equivalence with the original scale. The total sample included 106 breast cancer survivors.

Permission from original author(s) of the scale: Yes
Original scale authors involved in study team: No

**Measurement Property Assessment**

**1. Structural Validity (mp1)**
**Property Rating**: ?
**Data**: The study did not perform a confirmatory or exploratory factor analysis to establish the structural validity of the Tamil version of the FCR7.
**Methodological Quality**: Inadequate
**Quality of Evidence**: Very Low
**Justification**: According to COSMIN standards, the structural validity of a translated version should be re-evaluated in the new language and cultural context to confirm that the factor structure remains valid. The absence of such analysis limits the ability to confirm whether the scale retains its intended unidimensionality or measurement structure in Tamil breast cancer survivors.

**2. Internal Consistency (mp2)**
**Property Rating**: ?
**Data**: Cronbach's $\alpha = 0.864$ for the translated Tamil version of the FCR7. However, COSMIN prohibits interpretation of internal consistency when structural validity is unknown or insufficient.
**Methodological Quality**: Inadequate
**Quality of Evidence**: Very Low
**Justification**: While alpha exceeds 0.70, COSMIN requires at least low-quality evidence for sufficient structural validity to interpret internal consistency. Due to insufficient structural validity, internal consistency could not be determined and was rated as indeterminate.

**3. Reliability (mp3)**
**Property Rating**: +
**Data**: ICC = 0.910 based on a 15-day test–retest interval among 32 breast cancer survivors
**Methodological Quality**: Adequate
**Quality of Evidence**: Moderate
**Justification**: ICC exceeds the 0.70 threshold, indicating sufficient test–retest reliability. The subsample size (n = 32) falls below the COSMIN threshold of 50, resulting in a downgrade.

**4. Measurement Error (mp4)**
**Property Rating**: ?
**Data**: No SEM, SDC, MIC, or LoA were reported.
**Methodological Quality**: Inadequate
**Quality of Evidence**: Very Low
**Justification**: No information provided to assess measurement error.

**5. Hypothesis Testing for Construct Validity (mp5)**

**Property Rating**: +
**Data**: Spearman correlations with: FACT-B: r = –0.259, p = 0.01; IES-R: r = 0.270, p = 0.01
Both values exceeded the critical threshold of r = 0.197 for n = 106 at the 0.05 level.
**Methodological Quality**: Very Good
**Quality of Evidence**: High
**Justification**: Two theoretically supported hypotheses were tested and confirmed. Statistical significance, sample size, and construct selection support the rating.

**6. Cross-Cultural Validity (mp6)**
**Property Rating**: ?
**Data**: No DIF or multi-group CFA was conducted.
**Methodological Quality**: Inadequate
**Quality of Evidence**: Very Low
**Justification**: While a formal translation process was followed, there was no statistical testing of item equivalence across groups.

**7. Criterion Validity (mp7)**
**Property Rating**: +
**Data**: FCR7-Tamil was significantly correlated with: FACT-B: r = –0.259, p = 0.01; IES-R: r = 0.270, p = 0.01, although only IES is considered a closely related concept to FCR.
**Methodological Quality**: Adequate
**Quality of Evidence**: Moderate
**Justification**: COSMIN allows conceptually related tools when no gold standard exists; evidence supports a sufficient rating. Criterion validity is supported, though full assessment through ROC analysis could strengthen this evidence.

**8. Responsiveness (mp8)**
**Property Rating**: ?
**Data**: Not assessed
**Methodological Quality**: Inadequate
**Quality of Evidence**: Very Low
**Justification**: No change scores or longitudinal data were reported.

**Overall Summary and Quality Determination**

Nandakumar et al. [48] developed and validated a Tamil version of the FCR7 among 106 breast cancer survivors. Translation procedures followed international guidelines and included both expert and patient input. Cronbach's alpha (0.864) and test–retest ICC (0.910) suggest strong reliability, though internal consistency cannot be formally rated due to the absence of structural validity data. Construct and criterion validity were supported through correlations with FACT-B and IES-R, although no ROC or AUC were reported. No analysis of structural validity, measurement error, cross-cultural validity, or responsiveness was conducted.

**COSMIN Category Determination**

**Category B:** The PROM shows promising reliability and validity, but structural validity was not assessed, and internal consistency cannot be interpreted according to COSMIN rules. It is therefore classified as Category B , requiring further validation.

**Conclusion**

The FCR7-Tamil shows strong reliability and evidence of construct and criterion validity in Tamil-speaking breast cancer survivors. Further psychometric work is needed to evaluate its structural validity and responsiveness before broad clinical implementation.

**Decat Bergerot et al., 2023 FCR4/FCR7 [31]**

**Reference:** Bergerot, C. D., De Oliveira, M. A. R., Faria, C. A. P., Kowalski, L. P., Brandão, T., & Hovey, E. (2023). Cultural adaptation and psychometric properties of the Brazilian versions of the Fear of Cancer Recurrence 4/7 and Fear of Cancer Recurrence Inventory short form. Supportive Care in Cancer, 31(5), 1–11. https://doi.org/10.1007/s00520-023-07743-7

**Content Validity Assessment: Sufficient**

The authors reached out to the authors of the original FCR4/7 scales [43] to obtain permission to translate, culturally adapt and validate the Portuguese version. The FCR4 and FCR7 were translated into Brazilian Portuguese following Beaton et al.'s [58] cross-cultural adaptation guidelines, including forward translation by two independent bilingual translators, synthesis, back translation by two other translators, expert committee review, and pretesting. Ten patients participated in cognitive debriefing interviews, confirming clarity and cultural appropriateness of the adapted versions. No items required modification following pretesting. The sample population included 100 localized breast cancer and 100 metastatic heterogeneous cancer patients.

Permission from original author(s) of the scale: Yes
Original scale authors involved in study team: Yes

**Measurement Property Assessment:**

**1. Structural Validity (mp1)**
**Property Rating**: +
**Data**: CFA results supported a unidimensional structure for both FCR4 and FCR7, and all items from both have factor loading ≥ 0.40 (sufficient): FCR4: $\chi^2 = 1.921$, df = 2, p = 0.383; $\chi^2/df = 0.960$; CFI = 1.00; RMSEA = 0.0001 (90% CI: 0.000–0.14); SRMR = 0.0001; FCR7: CFA supported a unidimensional structure for the FCR7. Fit indices reported: CFI = 0.971 (≥ 0.95, sufficient), RMSEA = 0.099 (above threshold), RMR = 0.036 (sufficient, < 0.08).
**Methodological Quality**: Adequate
**Quality of Evidence**: Moderate
**Justification**: COSMIN permits a "+" rating if at least one of the model fit indices meets the threshold. FCR4 meets all fit indices. For FCR7, although RMSEA exceeded the cutoff (>0.06), CFI and SRMR met COSMIN thresholds (CFI ≥ 0.95, SRMR ≤ 0.08), supporting a sufficient rating. Evidence downgraded to moderate due to mixed model fit in FCR7. However, no variance explained were reported for both FCR4 and FCR7, and COSMIN still prefers this be shown when possible, especially under classical test theory (CTT). Thus, the methodological quality was downgraded to adequate.

**2. Internal Consistency (mp2)**
**Property Rating**: +
**Data**: FCR4: Cronbach's $\alpha = 0.879$ (95% CI: 0.855–0.904); FCR7: Cronbach's $\alpha = 0.894$ (95% CI: 0.874–0.914).
**Methodological Quality**: Very Good

**Quality of Evidence**: High
**Justification**: Internal consistency exceeded the 0.70 threshold for both versions, and structural validity was rated sufficient, allowing for full interpretation.

**3. Reliability (mp3)**

**Property Rating**: ?
**Data**: Test–retest reliability was not assessed.
**Methodological Quality**: Inadequate
**Quality of Evidence**: Very Low
**Justification**: No ICCs or repeated measures were reported; Lack of test-retest data leads to a very low rating for this property.

**4. Measurement Error (mp4)**

**Property Rating:** ?
**Data:** No SEM, SDC, MIC, or LoA reported
**Methodological Quality:** Inadequate
**Quality of Evidence:** Very Low
**Justification:** No measurement error data, resulting in a very low rating.

**5. Hypothesis Testing for Construct Validity (mp5)**

**Property Rating:** ?
**Data:** The study attempted group comparisons by cancer stage, but the models failed to converge. No predefined hypotheses were tested.
**Methodological Quality:** Inadequate
**Quality of Evidence:** Very Low
**Justification:** No correlation with other validated measures was reported. No a priori hypotheses were stated. Therefore, this property is unassessed.

**6. Cross-Cultural Validity (mp6)**

**Property Rating:** ?
**Data:** The FCR4 and FCR7 were administered to two cancer groups (localized vs metastatic) across 5 Brazilian states. The authors state that model convergence failed for group comparisons and no DIF or MG-CFA was conducted.
**Methodological Quality:** Inadequate
**Quality of Evidence:** Very Low
**Justification:** Although multiple regions and cancer stages were included, no statistical analysis (e.g., DIF or MG-CFA) was performed to test equivalence across groups. COSMIN requires such tests for cross-cultural validity. The authors have put forth that the examination of the psychometric criteria by subgroups was not feasible, likely due to the relatively small subgroup sample sizes (n =100).

**7. Criterion Validity (mp7)**

**Property Rating:** ?
**Data:** Criterion validity was not assessed. No data were provided on ROC, AUC, sensitivity, specificity, or correlations with a gold standard or a clearly related construct.

**Methodological Quality:** Inadequate
**Quality of Evidence:** Very Low
**Justification:** According to COSMIN guidelines, criterion validity should be assessed using a clear gold standard, or at minimum, a highly related construct that demonstrates strong correlations (typically >0.70). In the absence of a true gold standard for FCR, a proxy may be used, but only if it is conceptually close. Thus, methodological quality is rated as inadequate, and the quality of evidence is very low.

**8. Responsiveness (mp8)**

**Property Rating:** ?
**Data:** Not assessed
**Methodological Quality:** Inadequate
**Quality of Evidence:** Very Low
**Justification:** Responsiveness was not evaluated in the study.

**Overall Summary and Quality Determination**

Bergerot et al. [31] culturally adapted and evaluated the Brazilian Portuguese FCR4 and FCR7 among 200 cancer survivors. Content validity was supported through a standardized translation process and cognitive debriefing. Structural validity was rated sufficient for both tools, though no variance explained was reported. Internal consistency was strong for both versions. However, many properties remain indeterminate for test–retest reliability, construct validity, criterion validity, measurement error, cross-cultural validity, and responsiveness, as they were untested.

**COSMIN Category Determination**

**Category A:** The FCR4 and FCR7 meet COSMIN's criteria for Category A: sufficient content validity, at least low-quality evidence for sufficient structural validity, and high internal consistency. While further research on cross-cultural validity, test–retest reliability, and responsiveness is warranted, both versions are recommended for clinical and research use in Brazilian Portuguese-speaking adult cancer populations with localized breast cancer and any type of metastatic cancer.

**Conclusion**

The Brazilian Portuguese FCR4/7 version appears to be reliable and valid for initial use in Brazilian Portuguese-speaking adult cancer populations with localized breast cancer and any type of metastatic cancer. While content, structural, and internal consistency support their use, further research is needed to confirm their stability, responsiveness, and measurement equivalence across subgroups.

### 3.3 Concerns About Recurrence Questionnaire (CARQ) CARQ-4 [49]): Validation of the New PROM

**Thewes et al., 2015 / CARQ-4 [49]**

**Reference:** Thewes, B., Zachariae, R., Christensen, S., Nielsen, T., & Butow, P. (2015). The Concerns About Recurrence Questionnaire: Validation of a brief measure of fear of cancer recurrence amongst Danish and Australian breast cancer survivors. Journal of Cancer Survivorship, 9(1), 68-79. https://doi.org/10.1007/s11764-014-0383-1

**Content Validity Assessment: Sufficient**
The CARQ-4 was developed through expert adaptation of items from existing measures, feedback from breast cancer survivors, and psychometric testing across two international samples. The process ensured construct relevance and linguistic appropriateness for breast cancer survivors in both Australia and Denmark. The adaptation involved item generation, expert review, and forward-backward translation methods. Thus, content validity is assumed to be sufficient, based on expert input and the adaptation process, which included translation and pretesting for cultural relevance.

Permission from original author(s) of the scale: New PROM developed by first author
Original scale authors involved in study team: Yes

**Measurement Property Assessment**

**1. Structural Validity (mp1)**

**Property Rating:** +
**Data:** EFA in the Australian sample showed unidimensionality (CARQ-4 explained 72% variance); CFA in the Danish sample showed good fit (CFI = 0.99; NFI = 0.95; RMSEA = 0.12).
**Methodological Quality:** Very Good
**Quality of Evidence:** High
**Justification:** Meets COSMIN criteria with strong model fit across samples. Item 5 (CARQ-5) was excluded due to poor factor loadings.

**2. Internal Consistency (mp2)**

**Property Rating:** +
**Data:** Cronbach's alpha values were $\alpha$ = 0.87 (Australia) and $\alpha$ = 0.88 (Denmark) for the CARQ-4, indicating high internal consistency across both populations. Internal consistency for the CARQ-3 was $\alpha$ = 0.90 (Australia) and $\alpha$ = 0.91 (Denmark). The structural validity of the CARQ-4 was supported through EFA and CFA, justifying the internal consistency rating.
**Methodological Quality:** Very Good
**Quality of Evidence:** High
**Justification:** COSMIN criteria for sufficient internal consistency ($\alpha \geq 0.70$ and confirmed unidimensional structure) were met in both samples.

**3. Reliability (mp3)**

**Property Rating:** +
**Data:** Test–retest reliability over approximately 14 days (range 7–21 in Australia, 6–22 in Denmark) showed strong correlations. For CARQ-4: r = 0.74 (Australia), r = 0.83 (Denmark). For CARQ-3: r = 0.70 (Australia), r = 0.83 (Denmark). Monte Carlo simulated estimates also confirmed reliability (CARQ-4 Australia: 0.76).
**Methodological Quality:** Very Good
**Quality of Evidence:** High
**Justification:** Sufficient test–retest stability was observed in both samples with appropriate intervals, and reported R-values exceed COSMIN's minimum threshold of ≥0.70.

**4. Measurement Error (mp4)**

**Property Rating:** ?
**Data:** Measurement error was not explicitly reported.
**Methodological Quality:** Inadequate
**Quality of Evidence:** Very Low
**Justification:** Lack of measurement error data leads to an indeterminate rating for this property.

**5. Hypothesis Testing for Construct Validity (mp5)**

**Property Rating:** +
**Data:** Construct validity was evaluated in the Australian sample (N = 218). For the CARQ-4, strong correlations were found with FCRI-Total (r = 0.78) and FCRI-Severity (r = 0.78). Moderate correlations were observed with anxiety (DASS: r = 0.47), health anxiety (WI-7: r = 0.35), and generalized anxiety (GAD-Q-IV: r = 0.50). The CARQ-3 demonstrated comparable results: r = 0.76 (FCRI-Total), r = 0.73 (FCRI-Severity), r = 0.46 (DASS), r = 0.34 (WI-7), and r = 0.49 (GAD-Q-IV).
**Methodological Quality:** Very Good
**Quality of Evidence:** High
**Justification:** At least three predefined hypotheses were confirmed with directionally appropriate and statistically significant correlations using well-established comparator PROMs.

**6. Cross-Cultural Validity (mp6)**

**Property Rating:** –
**Data:** The CARQ-4 was translated into Danish and administered in both Danish and Australian samples. However, Rasch analysis indicated DIF by age and language, suggesting cultural differences.
**Methodological Quality:** Very Good
**Quality of Evidence:** Moderate
**Justification:** DIF observed across language versions indicates insufficient cross-cultural validity. No score equating or correction strategies were implemented, and COSMIN requires evidence of measurement invariance or DIF testing and resolution for cross-cultural validity to be rated sufficient.

**7. Criterion Validity (mp7)**

**Property Rating:** +
**Data:** ROC analysis, Against FCRI-Severity (cut-off ≥13): CARQ-4 AUC = 0.90; sensitivity = 85%, specificity = 81% at cut-off ≥12.
**Methodological Quality:** Very Good
**Quality of Evidence:** High
**Justification:** The high AUC value provides strong support for criterion validity.

**8. Responsiveness (mp8)**

**Property Rating:** ?
**Data:** Responsiveness was not assessed in this study.
**Methodological Quality:** Inadequate
**Quality of Evidence:** Very Low
**Justification:** No pre-post intervention or longitudinal change data provided leads to an indeterminate rating.

**Overall Summary and Quality Determination**

The CARQ-4, developed by Thewes et al. [49] was developed and validated as a brief, unidimensional measure of fear of cancer recurrence in two distinct samples of breast cancer survivors from Australia (n = 218) and Denmark (n = 2001). Content validity was supported through expert review and pre-testing, though cognitive interviews were not conducted. Structural validity was rated sufficient based on EFA results in the Australian sample (72% variance explained) and CFA in the Danish sample (CFI = 0.99, RMSEA = 0.12). Internal consistency was high ($\alpha$ = 0.87–0.88), and test–retest reliability met COSMIN criteria (r = 0.74–0.83 across countries and versions). Construct validity was confirmed through strong correlations with the FCRI, DASS, and GAD-Q-IV. Criterion validity was supported by ROC analysis against the FCRI-Severity scale (AUC = 0.90), with sensitivity = 85% and specificity = 81% at a cut-off of ≥12. Cross-cultural validity was rated insufficient due to significant differential item functioning between language versions. Measurement error and responsiveness were not assessed.

**COSMIN Category Determination**

**Category A:** The CARQ-4 qualifies as a Category A PROM for breast cancer survivors, meeting COSMIN criteria for sufficient content validity, structural validity, internal consistency, test–retest reliability, and criterion validity, with at least moderate-quality evidence. Although cross-cultural validity was insufficient due to differential item functioning across languages, and measurement error and responsiveness were not assessed, the English version demonstrates strong psychometric properties for clinical and research use. The Danish version, however, does not meet COSMIN standards for use due to cross-cultural limitations.

**Conclusion**
The CARQ-4 by Thewes et al. [49]is a brief, psychometrically robust screening tool for assessing fear of cancer recurrence in breast cancer survivors. The English version is recommended for both clinical and research screening applications. Further research is warranted to address responsiveness and cross-cultural validity, particularly for use in non-English populations.

**3.4 FCR-1 (Rudy et al., 2020) [50] - Validation of a New PROM, and Adaptations in New Languages and Populations**
**Rudy et al., 2020 / FCR-1 [50]**

**Reference:** Rudy, L., Maheu, C., Körner, A., Lebel, S., & Gélinas, C. (2020). The FCR-1: Initial validation of a single-item measure of fear of cancer recurrence. *Psycho-Oncology, 29*(5), 788–795. https://doi.org/10.1002/pon.5350

**Content Validity Assessment: Sufficient**
Rudy et al. [50] developed the one-item FCR (FCR-1) through iterative feedback from breast cancer survivors, clinicians, and researchers, ensuring clarity and relevance in assessing FCR. Modeled after the Edmonton Symptom Assessment System (ESAS), the FCR-1 was presented as either a 0 to 10 scale or 0 to 100 percent scale, depending on patient preference of reporting, to capture subjective FCR. The wording was finalized after review by two cancer patients, a nurse, and two psychotherapists with expertise in FCR, ensuring clarity and clinical relevance. The process meets COSMIN standards for relevance and comprehensibility of content. Thus, content validity is rated as sufficient, supported by robust expert input and modeled after an established PROM (ESAS) that affirms the scale's clinical relevance.

Permission from original author(s) of the scale: New PROM developed by first author
Original scale authors involved in study team: Yes

**Measurement Property Assessment**

**1. Structural Validity (mp1)**

**Rating:** Not Applicable (N/A)
**Justification:** Structural validity cannot be evaluated for single-item instruments, as dimensionality assessments (e.g., factor analysis) require multiple items. Per COSMIN guidance, this domain is considered not applicable for single-item PROMs.

**2. Internal Consistency (mp2)**

**Rating:** N/A
**Justification:** Internal consistency is not applicable for single-item PROMs, as reliability coefficients such as Cronbach's alpha require multiple items to assess inter-item correlations. This is consistent with COSMIN recommendations.
**Justification:** N/A

**3. Reliability (mp3)**

**Property Rating:** +
**Data:** For single-item instruments, COSMIN accepts criterion validity as evidence of reliability when a gold standard is used. Given that the FCRI-SF served as the reference standard and demonstrated strong ROC performance, this counts as indirect evidence of reliability.
**Conclusion:** Sufficient reliability (via criterion validity) per COSMIN criteria for single-item tools.
**Methodological Quality:** Very Good
**Quality of Evidence:** High
**Justification:** Reliability, assessed through criterion validity for single-item instrument, is supported by a well-executed ROC analysis using a validated external comparator (FCRI-SF). The AUC indicates excellent diagnostic accuracy. See mp7 for specific values obtained for criterion validity.

**4. Measurement Error (mp4)**

**Property Rating:** ?
**Data:** No data reported.
**Methodological Quality:** Inadequate
**Quality of Evidence:** Very Low
**Justification:** Indeterminate.

**5. Hypothesis Testing for Construct Validity (mp5)**

**Rating:** +
**Data:** A priori hypotheses were stated and confirmed. Moderate correlations were observed between the FCR-1 [50] and the FCRI [5] ($r = 0.395$, $p = .010$); Mishel Uncertainty in Illness Scale [61] ($r = 0.493$, $p = .001$); and the Reassurance Questionnaire ($r = 0.325$, $p = .044$), as predicted. No significant correlations were found with unrelated clinical and demographic variables, supporting discriminant validity.

**Methodological Quality:** Very Good
**Quality of Evidence:** High
**Justification:** Construct validity is considered sufficient and well-supported based on clearly defined hypotheses, appropriate comparator measures with established psychometric properties, and confirmation of predicted patterns of correlation.

**6. Cross-Cultural Validity (mp6)**

**Rating:** ?
**Justification:** Group comparisons by demographic and treatment factors were conducted, though no formal full measurement invariance analyses (e.g., DIF, measurement invariance testing) were conducted to evaluate equivalence with the original version.
**Methodological Quality:** Inadequate
**Quality of Evidence:** Very Low
**Justification:** Cross-cultural validity was not formally assessed. While group comparisons were conducted, no DIF or invariance testing was conducted. This does not meet COSMIN standards for evaluating cross-cultural equivalence.

**7. Criterion Validity (mp7)**

**Rating:** +
**Data:** ROC analysis was conducted using the FCRI-Severity (cut-off ≥22) as an external reference. The FCR-1 showed an AUC of 0.853 (95% CI: 0.71–1.00, $p < .001$), with sensitivity = 70%, specificity = 89.5%, PPV = 77.8%, and NPV = 85.0% at an optimal cut-off of 45.0 on the 0 to 100 percentage scale or 4.5 on the 0 to 10 scale.
**Methodological Quality:** Very Good
**Quality of Evidence:** High
**Justification:** Criterion validity is sufficient, supported by strong ROC performance using a theoretically and empirically aligned external reference measure.

**8. Responsiveness (mp8)**

**Rating:** +
**Data:** Responsiveness was evaluated across six timepoints during an intervention using repeated-measures ANOVA and paired sample t-tests. Significant changes were observed in FCR-1 scores between sessions 1 and 6 ($t = 4.529$, $p < .001$, $d = 0.79$), as well as between sessions 4 and 5 ($d = 0.36$) and 5 and 6 ($d = 0.30$).
**Methodological Quality:** Very Good
**Quality of Evidence:** High
**Justification:** Responsiveness is well-supported. The change in FCR-1 scores was measured across six timepoints using a robust RCT design and confirmed through hypothesis-driven analysis with clinically meaningful effect sizes.

**Overall Summary and Quality Determination**
The FCR-1 is a single-item screening tool for FCR, developed by Rudy et al. [50] and adapted from the Edmonton Symptom Assessment System. Content validity was supported through expert consultation and input from cancer survivors. Although administered primarily using a 0–100 scale, participants could opt for a 0–10 response format—a methodological detail later clarified through personal communication

with the FORT-RCT team [54].

Construct validity was confirmed through expected correlations with the FCRI [5] (r = 0.395) and illness uncertainty [61] (r = 0.493). Criterion validity was demonstrated using ROC analysis against the FCRI-Severity subscale [28] (AUC = 0.85). Responsiveness was also supported, with FCR-1 scores significantly declining over the course of the FORT intervention (Cohen's $d$ = 0.79). As a single-item PROM, structural validity and internal consistency were not applicable. Test–retest reliability, measurement error, and cross-cultural validity were not assessed.

Despite these limitations, the FCR-1 received a COSMIN Category A rating based on strong evidence for content, construct, criterion validity, and responsiveness, and is recommended for use as a brief screening measure in clinical contexts.

**Category Determination Based on COSMIN Criteria:**

**Category A:** The FCR-1 meets COSMIN criteria for Category A for single-item PROMs: it demonstrates sufficient content validity, and sufficient evidence of either criterion validity or responsiveness. As such, it is recommended for use as a brief screening tool for fear of cancer recurrence.

**Conclusion**
The FCR-1 [50] is recommended as a brief, psychometrically sound measure for screening fear of cancer recurrence in clinical settings. It shows strong evidence for validity and responsiveness, making it useful for identifying patients with elevated FCR who may benefit from further assessment or intervention. Future studies should evaluate its test–retest reliability and cross-cultural equivalence to strengthen the evidence base for broader use across settings and populations.

**Smith et al., 2023 / FCR-1r [51]**

**Reference:** Smith, A., Gao, M., Tran, M., Ftanou, M., Jegathees, S., Wu, V., Jefford, M., Lynch, F., Dhillon, H. M., Shaw, J., McDowell, L., White, A., Halloran, C., Wiesenfeld, D., & Bamgboje-Ayodele, A. (2023). Evaluation of the validity and screening performance of a revised single-item fear of cancer recurrence screening measure (FCR-1r). *Psycho-oncology*, *32*(6), 961–971. https://doi.org/10.1002/pon.6139

**Content Validity Assessment: Sufficient with low quality evidence**
The FCR-1r was developed as a single-item PROM modeled on the ESAS format and refined through iterative consultation with 10 stakeholders, including cancer survivors and clinicians [51]. A short explanatory phrase was added to define "fear of recurrence or progression," intended to clarify the item for routine use in oncology settings. This process meets COSMIN's definition of sufficient content validity for single-item tools—emphasizing relevance, comprehensiveness, and comprehensibility—provided there is evidence of patient input and cognitive testing. However, the methodological quality is rated as doubtful due to the missing detail on whether participants with high vs. low FCR were equally represented and due to the lack of a saturation process for item development.
Moreover, although the FCR-1r was described as a "revision" of the original FCR-1 [50], the developers of the FCR-1 were neither contacted nor involved in the adaptation process. The item's wording and scale format were changed without input from the original authors, and the revised version was not tested against the original FCR-1 within the same sample. These omissions represent a serious flaw from a COSMIN perspective on content validity, particularly for adapted instruments, which should adhere to

established best practices from ISPOR, WHO, and COSMIN—each of which recommends contacting the original developer to preserve conceptual equivalence.

Permission from original author(s) of the scale: Not reported
Original scale authors involved in study team: No

**Measurement Property Assessment**

**1. Structural Validity (mp1)**

**Rating:** Not Applicable (N/A)
**Justification:** Structural validity cannot be evaluated for single-item instruments, as dimensionality assessments (e.g., factor analysis) require multiple items. Per COSMIN guidance, this domain is considered not applicable for single-item PROMs.
**Conclusion:** For single-item instruments, COSMIN does not require evidence of structural validity; instead, sufficient content (face) validity and any evidence of reliability (e.g., internal consistency, criterion validity) are acceptable for Category A assignment.

**2. Internal Consistency (mp2)**

**Rating:** N/A
**Justification:** Internal consistency is not applicable for single-item PROMs, as reliability coefficients such as Cronbach's alpha require multiple items to assess inter-item correlations. This is consistent with COSMIN recommendations.
**Justification:** N/A
**Conclusion:** For single-item instruments, COSMIN expects any form of evidence of reliability (e.g., internal consistency, criterion validity), but not internal consistency as non-relevant, to be done with sufficient content validity to be acceptable for Category A assignment.

**3. Reliability (mp3)**

**Property Rating:** +
**Data:** For single-item instruments, COSMIN accepts criterion validity as evidence of reliability when a gold standard is used. Given that the FCRI-SF served as the reference standard and demonstrated strong ROC performance, this counts as indirect evidence of reliability.
**Conclusion:** Sufficient reliability (via criterion validity) per COSMIN criteria for single-item tools.
**Methodological Quality:** Very Good
**Quality of Evidence:** Low
**Justification:** Reliability, assessed through criterion validity for single-item instrument, is supported by a well-executed ROC analysis using a validated external comparator (FCRI-SF). The AUC indicates excellent diagnostic accuracy. See mp7 for specific values obtained for criterion validity. While the analytic approach was robust, the methodological quality is downgraded due to the inclusion of non-purpose-collected data. The addition of a serious flaw in content validity is downgraded to very low quality of evidence.

**4. Measurement Error (mp4)**

**Property Rating:** ?
**Data:** No data reported.
**Methodological Quality:** Inadequate
**Quality of Evidence:** Very Low
**Justification:** Indeterminate.

**5. Hypothesis Testing for Construct Validity (mp5)**

**Property Rating**: +
**Data**: A priori hypotheses were stated. FCR-1r significantly correlated with FoP-Q-SF ($r = 0.67$), anxiety ($r = 0.54$), depression ($r = 0.52$), and emotional distress ($r = 0.57$), all $p < .001$. Discriminant hypotheses were also supported.
**Methodological Quality**: Adequate (due to partial reliance on data collected during routine care in Study 2, not specifically designed for psychometric validation)
**Quality of Evidence**: Moderate
**Justification**: Construct validity is supported. While hypotheses were clearly stated and confirmed, part of the data came from routine clinical collection, limiting methodological rigor, and leading to a COSMIN downgrade.

**6. Cross-Cultural Validity (mp6)**

**Property Rating**: ?
**Data**: No cross-cultural adaptation or measurement invariance testing was conducted.
**Methodological Quality**: Inadequate
**Quality of Evidence**: Very Low
**Justification**: Cross-cultural validity not evaluated.

**7. Criterion Validity (mp7)**

**Rating:** +
**Data:** ROC analysis was conducted using the FCRI-SF cut-off of ≥22 as the reference standard for clinical FCR . Based on this, the FCR-1r achieved an AUC = 0.91 (95% CI: 0.85–0.97, $p < .0001$), indicating excellent discrimination. A cut-off score of ≥5/10 on the FCR-1r yielded optimal classification metrics, with sensitivity and negative predictive value (NPV) above 90%, and specificity close to 80%.
**Methodological Quality:** Adequate (due to partial reliance on routine care data not collected solely for this purpose).
**Quality of Evidence:** Moderate
**Justification:** Criterion validity is supported by a well-executed ROC analysis using a validated external comparator (FCRI-SF) [28]. The AUC indicates excellent diagnostic accuracy. While the analytic approach was robust, the methodological quality is downgraded due to the inclusion of non-purpose-collected data.

**8. Responsiveness (mp8)**

**Rating:** ?
**Data:** Responsiveness of the FCR-1r was not formally evaluated in Smith et al. [51]. The tool was assessed using cross-sectional data in Study 1 and data collected during routine care in Study 2. No longitudinal

data, pre–post intervention comparisons, or change metrics (e.g., effect sizes such as Cohen's d) were reported.
**Methodological Quality:** Inadequate
**Quality of Evidence:** Very Low
**Justification:** Responsiveness has not yet been assessed. Future research is needed to determine the FCR-1r's sensitivity to change over time, which would confirm its utility for monitoring FCR in clinical or intervention contexts.

**Overall Summary and Quality Determination**
The FCR-1r, developed by Smith et al. [51], is a single-item screening measure for FCR, modeled after the ESAS symptom format. The tool underwent iterative development involving stakeholder input and was evaluated across two data sources: a cross-sectional validation study and routine clinical care data. It demonstrated sufficient content validity, strong construct validity (mp5), and excellent criterion validity (mp7), including high AUC values for FCRI-SF-based classification. However, measurement error, responsiveness, and cross-cultural validity were not evaluated. Importantly, while the FCR-1r is labeled a "revision" of the original FCR-1, the developers did not involve or consult the original authors of the FCR-1 [50]. The adaptation introduced changes to the item wording and scale format (0–10 numeric scale vs. 0–100 visual analogue) without comparative validation. COSMIN and international translation guidelines (e.g., ISPOR, Beaton, WHO) recommend consultation with original scale developers when modifying or adapting PROMs to preserve conceptual fidelity.

**Category Determination Based on COSMIN Criteria:**

**Category A:** The FCR-1r is assigned to Category A based on sufficient content validity (supported by stakeholder input) but of low quality, and with sufficient criterion validity (AUC = 0.91). As per COSMIN criteria for single-item PROMs. Structural validity and internal consistency are not applicable.

**Conclusion**
The FCR-1r is a promising, brief, and psychometrically sound tool for cross-sectional screening of fear of cancer recurrence [51]. It demonstrates excellent diagnostic accuracy and alignment with clinical symptom screening formats like the ESAS. However, it has not yet demonstrated responsiveness or test–retest reliability, precluding its current use for longitudinal monitoring. Furthermore, the lack of collaboration with the original FCR-1 developers and absence of direct comparison between the original and revised versions raise conceptual and ethical concerns. Thus, the FCR-1r should be interpreted as a parallel adaptation rather than a validated revision and requires further validation before routine adoption for repeated clinical assessments.

**Lyhne et al., 2023 / FCR-1 Danish [52]**

**Reference:** Lyhne, J. D., Smith, A. B., Timm, S., Simard, S., Jensen, L. H., Frostholm, L., & Fink, P. (2023). Validity and screening capacity of the FCR-1r for fear of cancer recurrence in long-term colorectal cancer survivors. *Supportive Care in Cancer, 31*, 690. https://doi.org/10.1007/s00520-023-08159-7

**Content Validity Assessment: Sufficient**

The FCR-1r [51] was translated into Danish by a trilingual administrator and the lead investigator, then field-tested with an age-matched sample including cancer survivors. No modifications were made following testing, supporting linguistic and conceptual equivalence. The process aligns with COSMIN

and ISPOR guidelines for translation and cultural adaptation. Importantly, the original authors of the FCR-1r [51] and FCRI-SF [28] were part of the study team, enhancing conceptual fidelity. Thus, content validity is rated as sufficient.

Permission from original author(s) of the scale: Yes
Original scale authors involved in study team: Yes

**Measurement Property Assessment**

**1. Structural Validity (mp1)**

**Rating:** Not Applicable (N/A)
**Justification:** Structural validity cannot be evaluated for single-item instruments, as dimensionality assessments (e.g., factor analysis) require multiple items. Per COSMIN guidance, this domain is considered not applicable for single-item PROMs.
**Conclusion:** For single-item instruments, COSMIN does not require evidence of structural validity; instead, sufficient content (face) validity and any evidence of reliability (e.g., internal consistency, criterion validity) are acceptable for Category A assignment.

**2. Internal Consistency (mp2)**

**Rating:** N/A
**Justification:** Internal consistency is not applicable for single-item PROMs, as reliability coefficients such as Cronbach's alpha require multiple items to assess inter-item correlations. This is consistent with COSMIN recommendations.
**Justification:** N/A
**Conclusion:** For single-item instruments, COSMIN expects any form of evidence of reliability (e.g., internal consistency, criterion validity), but not internal consistency as non-relevant, to be done with sufficient content validity to be acceptable for Category A assignment.

**3. Reliability (mp3)**

**Property Rating:** +
**Data:** For single-item instruments, COSMIN accepts criterion validity as evidence of reliability when a gold standard is used. Given that the FCRI-SF [28] (cut-off ≥22) served as the reference standard and demonstrated strong ROC performance, this counts as indirect evidence of reliability.
**Conclusion:** Sufficient reliability (via criterion validity) per COSMIN criteria for single-item tools.
**Methodological Quality:** Very Good
**Quality of Evidence:** High
**Justification:** Reliability, assessed through criterion validity for single-item instrument, is supported by a well-executed ROC analysis using a validated external comparator (FCRI-SF). The AUC indicates excellent diagnostic accuracy. See mp7 for specific values obtained for criterion validity.

**4. Measurement Error (mp4)**

**Property Rating:** ?
**Data:** No data reported.
**Methodological Quality:** Inadequate

**Quality of Evidence:** Very Low
**Justification:** Indeterminate.

**5. Hypothesis Testing for Construct Validity (mp5)**

**Property Rating**: +
**Data**: A priori hypotheses for convergent and divergent validity were confirmed with FCR-1r correlated with: FCRI-SF: r = 0.71; with subscales of the Short version Symptom Checklist-90-r (SCL) [62] - SCL-anx: r = 0.38; SCL-dep: r = 0.27; SCL-distress: r = 0.37;
**Methodological Quality**: Adequate
**Quality of Evidence**: Moderate
**Justification**: Construct validity is supported. All pre-specified directional hypotheses were statistically supported. Although correlation strengths were modest, they were theoretically sound and consistent with prior literature. Methodological quality is rated as adequate due to reliance on self-reported data from generic psychological tools (SCL) rather than closely related and relevant oncological FCR gold standard FCR-specific measure beyond the FCRI-SF.

**6. Cross-Cultural Validity (mp6)**

**Property Rating:** ?
**Data:** A group comparison was conducted between younger and older Danish cancer survivors, but not for language-based or ethnic subgroup equivalence.
**Methodological Quality:** Inadequate
**Quality of Evidence:** Very Low
**Justification:** Per COSMIN, true cross-cultural validity requires item-level DIF or MG-CFA, which were not performed

**7. Criterion Validity (mp7)**

**Rating:** +
**Data:** ROC analysis using the FCRI-SF (cut-off ≥22) for the total sample yielded an AUC = 0.93 (95% CI: 0.91–0.94), with an optimal FCR-1r cut-off ≥5 giving 93.5% sensitivity and 80.4% specificity.
**Methodological Quality:** Very Good
**Quality of Evidence:** High
**Justification:** Criterion validity is supported by a well-executed ROC analysis using a validated external comparator (FCRI-SF). The AUC indicates excellent diagnostic accuracy. The FCRI-SF has itself been validated against clinical interviews, qualifying it as a suitable reference standard.

**8. Responsiveness (mp8)**

**Rating:** ?
**Data:** Responsiveness was not assessed.
**Methodological Quality:** Inadequate
**Quality of Evidence:** Very Low
**Justification:** Responsiveness (i.e., sensitivity to change over time) was not evaluated.

**Overall Summary and Quality Determination**

The FCR-1r Danish version [52] demonstrated sufficient content validity and strong criterion validity (AUC = 0.93, sensitivity = 93.5%, specificity = 80.4%). As a single-item measure, it is exempt from structural validity and internal consistency requirements. Construct validity was adequately supported through hypothesis testing with appropriate psychological correlates. While measurement error, responsiveness, and cross-cultural validity were not evaluated, these limitations are acceptable under COSMIN guidance for single-item instruments.

The methodological quality was rated Very Good for both content and criterion validity domains, and Adequate for construct validity. The quality of evidence is therefore High for the key domains that apply for single item PROMs for assessing the quality of evidence of an outcome measurement instrument.

**Category Determination Based on COSMIN Criteria**

**COSMIN Category: A:** Per COSMIN guidance, single-item PROMs may be recommended (Category A) if they demonstrate sufficient content validity and any form of reliability. The FCR-1r Danish version meets both criteria: it was rigorously translated and field-tested with involvement from original authors of the FCR-1r [51] and demonstrated excellent diagnostic accuracy against the FCRI-SF [28]. It is recommended for FCR screening in long-term colorectal cancer survivors. However, it has not yet demonstrated responsiveness or test–retest reliability, precluding its current use for longitudinal monitoring.

**Supplemental B. Top 5 PROMs Recommended for Clinical Use**

This summary highlights the five PROMs most strongly supported by COSMIN ratings for brief screening and longitudinal follow-up of FCR. Recommendations are based on Category A status, evidence of responsiveness, cross-cultural validation, and feasibility in clinical contexts.

| PROM | Key Features | Strengths | Limitations / Notes |
|---|---|---|---|
| **FCR-1** (Rudy et al., 2020) | 1 item, 0–100 scale<br>Or 0–10 scale | Ultra-brief; demonstrated responsiveness; feasible in any setting | Single-item format limits domain coverage |
| **CWS** (Custers et al., 2018; multiple adaptations) | 6 items, 4-point Likert | Multiple cultural validations; brief and easy to administer | Responsiveness not demonstrated |
| **FCR-7** (Humphris et al., 2018; Chinese, Portuguese) | 7 items, 5-point Likert | Category A in select validations; very brief and feasible | Some translations in Category B; responsiveness not established |
| **FCRI-SF** (Simard & Savard, 2009; multiple validations) | 9 items, 4-point Likert | Widely validated across languages; strong internal consistency | Responsiveness evidence limited; best for cross-sectional use |
| **FoP-Q-SF** (Mehnert et al., 2006; multiple validations) | 12 items, 5-point Likert | Strong cross-cultural validations (Chinese, Portuguese, Malay, etc.); Category A | Responsiveness data lacking |
